# Rational Design, Synthesis and Pharmacological Evaluation of Chalcones as Dual-Acting Compounds—Histamine H_3_ Receptor Ligands and MAO-B Inhibitors

**DOI:** 10.3390/ijms27020581

**Published:** 2026-01-06

**Authors:** Dorota Łażewska, Agata Doroz-Płonka, Kamil Kuder, Agata Siwek, Waldemar Wagner, Joanna Karnafał-Ziembla, Agnieszka Olejarz-Maciej, Małgorzata Wolak, Monika Głuch-Lutwin, Barbara Mordyl, Oktawia Osiecka, Michał Juszczak, Katarzyna Woźniak, Małgorzata Więcek, Gniewomir Latacz, Anna Stasiak

**Affiliations:** 1 Department of Chemical Technology and Biotechnology of Drugs, Faculty of Pharmacy, Jagiellonian University Medical College in Kraków, Medyczna 9, 30-688 Kraków, Poland; 2 Department of Pharmacobiology, Faculty of Pharmacy, Jagiellonian University Medical College in Kraków, Medyczna 9, 30-688 Kraków, Poland; 3 Radioisotope and Functional Analysis Laboratory, Center for the Development of Therapies for Civilization and Age-Related Diseases, Jagiellonian University Medical College in Kraków, Medyczna 7A, 30-688 Kraków, Poland; 4 Department of Hormone Biochemistry, Faculty of Medicine, Medical University of Łódź, Żeligowskiego 7/9, 90-752 Łódź, Poland; 5 Laboratory of Cellular Immunology, Institute of Medical Biology, Polish Academy of Sciences, Lodowa 106, 93-232 Łódź, Poland; 6 Pharmacokinetics and Preliminary Toxicological Analysis Laboratory, Center for the Development of Therapies for Civilization and Age-Related Diseases, Jagiellonian University Medical College in Kraków, Medyczna 7A, 30-688 Kraków, Poland; 7 Department of Molecular Genetics, Faculty of Biology and Environmental Protection, University of Łódź, Pomorska 141/143, 90-236 Łódź, Poland

**Keywords:** chalcones, dual-target ligands, histamine H_3_ receptor ligands, monoamine oxidase B inhibitors, SPRM kinetic studies

## Abstract

Chalcone-based derivatives were designed as dual-acting ligands targeting the histamine H_3_ receptor (H_3_R) and monoamine oxidase B (MAO-B), based on the lead compound **DL76**. Three series of compounds (**1**–**18**) were synthesised and characterised, including simple chalcones (**1**–**9**) and piperidinyl chalcones (**10**–**18**). All piperidinyl derivatives exhibited nanomolar affinity for human H_3_R (hH_3_R), with compounds **10**–**12** achieving K_i_ values ≤ 30 nM. Simple chalcones showed potent human MAO-B (hMAO-B) inhibition (IC_50_: 0.85–337 nM), especially 3,4-dichloro derivatives. Compound **15** was the most active hybrid, with a K_i_ of 46.8 nM for hH_3_R and an IC_50_ of 212.5 nM for hMAO-B. Molecular docking and 250 ns simulations revealed stabilising interactions at both binding sites and clarified structural features behind dual activity. Preliminary ADMET profiling showed low Caco-2 permeability and rapid microsomal metabolism, mainly via hydroxylation. Compound **15** exhibited micromolar cytotoxicity in SH-SY5Y and HepG2 cells, induced G2/M arrest, disrupted mitochondrial homeostasis, and was genotoxic in Peripheral Blood Mononuclear Cells (PBMCs). Additionally, for H_3_R ligands (**15**, **DL76**, pitolisant), the study reports the first use of Surface Plasmon Resonance Microscopy (SPRM) to assess their interactions with this receptor. Therefore, piperidinyl chalcones show promise as ligands with dual action on H_3_R and MAO-B, useful in the treatment of neurodegeneration and/or CNS cancers.

## 1. Introduction

The histamine H_3_ receptor (H_3_R) belongs to the family of G protein-coupled receptors and, since its discovery in the 1980s, has continued to attract the interest of scientists [1,2]. This discovery changed the perception of histamine itself. Until then, it was considered only as a factor causing allergic and inflammatory effects, as well as influencing certain physiological functions, such as gastric juice secretion. This effect was associated with histamine-mediated activation of histamine H_1_ and/or H_2_ receptors [3]. The discovery revealed that histamine can also act as a neurotransmitter, and subsequent efforts to clone this receptor led to the identification of a new member of the histamine receptor family—the histamine H_4_ receptor [4]. The H_3_R is widely distributed throughout the brain, particularly in regions associated with memory and cognition. Its blockade increases the release of histamine and other neurotransmitters, such as acetylcholine, dopamine (DA), noradrenaline or serotonin [2]. The H_3_R was cloned in 1999 by Lovenberg et al. [5]. Further research has shown that it can exist in 20 isoforms, which differ in the number of amino acids; of these, only 7 can perform pharmacological functions due to the presence of critical areas responsible for agonist binding and/or signalling [6]. In the 40 years since the discovery of this receptor, many of its ligands have been synthesised [7,8,9]. Research has primarily focused on the search for ligands that inhibit this receptor’s activity, i.e., antagonists and inverse agonists (as the H_3_R exhibits high constitutive activity [10,11]). The obtained pharmacological studies suggested the utility of H_3_R antagonists/inverse agonists in the treatment of various human disorders, e.g., Alzheimer’s Disease, ADHD, Parkinson’s Disease (PD), schizophrenia, narcolepsy or allergy [7,8,9]. So far, only one H_3_R ligand, pitolisant, has entered the market as Wakix, a drug for the treatment of narcolepsy (EMA-2016, FDA-2019) [12] and as Ozawade (EMA-2021) for excessive daytime sleepiness associated with obstructive sleep apnea [13].

In recent years, H_3_R antagonists/inverse agonists have been synthesised as multi-target, mostly dual-target ligands (DTLs) [14,15]. Compounds were designed as structures showing antagonism/inverse agonism at the H_3_R while simultaneously affecting other biological targets.

**Figure 1:**
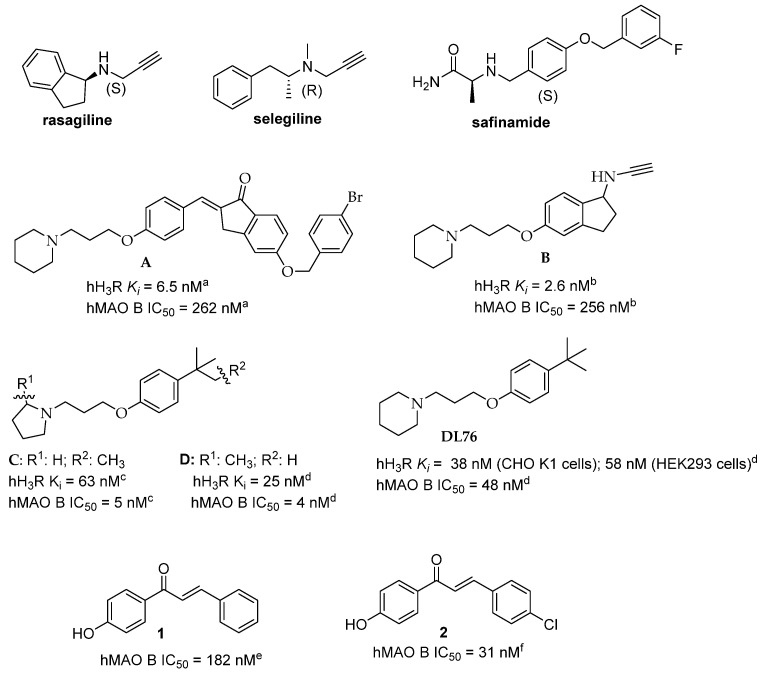
Structures of market MAO-B drugs (**top line**), dual target ligands, i.e., histamine H_3_ receptor ligands and MAO-B inhibitors (**middle lines**), and chalcones (**bottom line**). ^a^ data from Ref. [19]; ^b^ data from Ref. [20]; ^c^ data from Ref. [21]; ^d^ data from Ref. [23]; ^e^ data from Ref. [26]; ^f^ data from Ref. [27].

Among these biological targets, monoamine oxidase B (MAO-B) enzyme is important due to its crucial role in the pathogenesis of PD. MAO-B belongs to the family of MAOs that catalyse the deamination of neurotransmitters (e.g., DA) and release reactive oxygen species as by-products. MAO-B dominates in the human brain (*glia cells*) and deaminates β-phenylethylamine (PEA). PEA increases the synaptic levels of DA and blocks its reuptake into neurons. MAO-B activity increases with age and is elevated in certain diseases, such as PD. Inhibitors of MAO-B stop the activity of this enzyme and block the breakdown of DA. Moreover, they exhibit neuroprotective effects and reduce oxidative stress [16]. Three of them are currently used in medicine: selegiline, rasagiline and safinamide (Figure 1) [17]. The first two are irreversible MAO-B inhibitors that form covalent bonds with the flavin cofactor of MAO-B. Safinamide is a reversible inhibitor, mainly forming hydrogen bonds with preserved water molecules and protein residues in the active site of the MAO-B enzyme [18]. Reversible inhibition seems to be more advantageous for the drug’s safety profile because de novo protein synthesis is not required to restore enzymatic activity, thereby minimising toxic side effects.

So far, only a limited number of compounds have been reported that simultaneously exhibit affinity for H_3_R and inhibitory activity for MAO-B. Among them are indanone derivatives. The most potent compounds, **A** and **B**, are shown in Figure 1 [19,20]. They were characterised by very high affinity for human H_3_R (hH_3_R) and moderate inhibition of human MAO-B (hMAO-B). On the other hand, we obtained *tert*-butyl and *tert*-pentyl derivatives, with comparable activity for both biological targets, such as **DL76** (Figure 1), or higher inhibitory activity for hMAO-B, e.g., compounds **C** and **D** (Figure 1) [21,22,23].

Continuing our work in this field, we chose compound **DL76** as the lead structure for further modifications. Analysis of the literature directed our focus toward chalcones, which exhibit broad biological activity, acting on various biological targets, that potentially might be used in the treatment of PD. They have been reported to show MAO-B inhibition, adenosine receptor antagonism (A_2A_ and A_1_), catechol-*O*-methyltransferase inhibition, and Nrf2 activation [24]. Various structurally diverse chalcones have been described in the literature as MAO-B inhibitors, exhibiting different strengths of inhibition towards this enzyme [24,25]. However, for this study, we were interested in structurally simple chalcones, including compounds **1** and **2** (Figure 1) [26,27]. These compounds were characterised by good hMAO-B inhibitory activity with an IC_50_ below 200 nM. Moreover, we observed that the introduction of a chlorine atom into the molecule (position 4; compound **2**) resulted in a 6-fold increase in hMAO-B inhibition capacity (compound **1**). Thus, compound **2** was selected as the lead structure, and we decided to merge its core with our promising ligand **DL76**. Three series of compounds were designed based on these structures and the safinamide motif as described in Section 2.1. Compounds were synthesised and tested for both the hH_3_R and hMAO-B. Based on the results obtained, the most active DTL was selected for further preliminary ADMET studies. Furthermore, molecular docking to H_3_R and MAO-B was performed to characterise the possible interactions between these targets and the selected ligands.

## 2. Results and Discussion

### 2.1. Design of Compounds

**Scheme 1:**
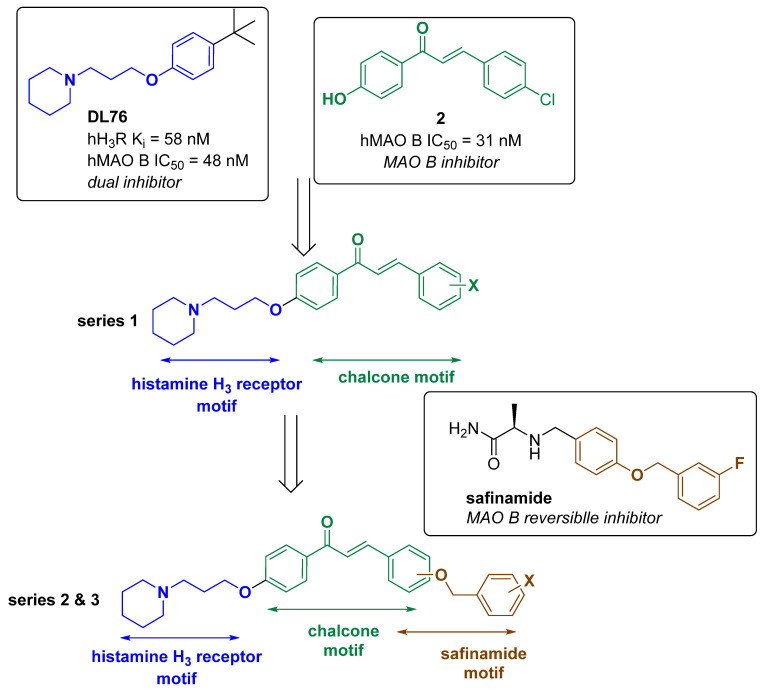
The idea of designing new dual target inhibitors—histamine H_3_ receptor ligands and MAO-B inhibitors.

**Scheme 2:**
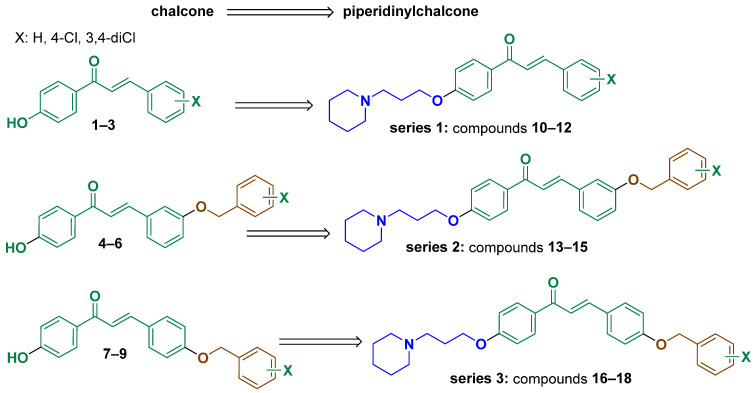
General structures of the designed three series of compounds.

As a result of our search for DTLs, we merged a motif characteristic of H_3_R, i.e., piperidinylpropoxy, with a chalcone moiety as shown in Scheme 1 and designed a series 1 (Scheme 2). Then, we decided to add a fragment derived from safinamide, and depending on the attachment, two series were obtained: series 2 and series 3 (Scheme 2). Additionally, to compare the effect of a fragment characteristic of H_3_R on MAO-B inhibition, corresponding simple chalcones (compounds **1**–**9**) were obtained for each series. This also included the resynthesis of compounds **1** and **2** for better comparison with the compounds studied in our laboratory.

### 2.2. Synthesis of Compounds

**Scheme 3:**
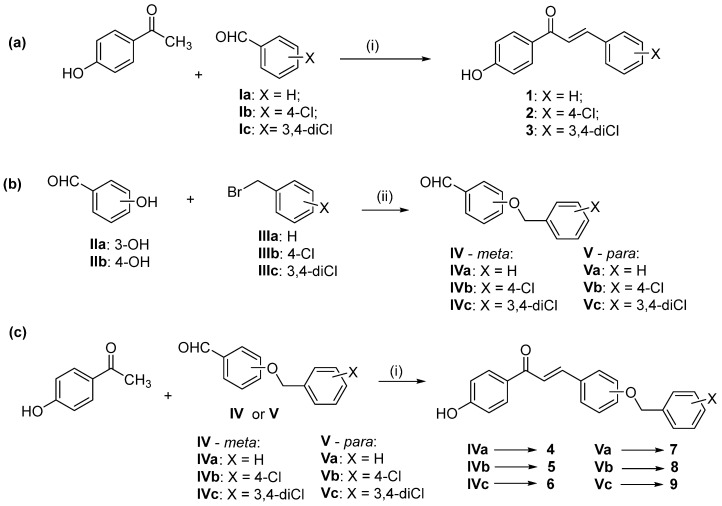
Synthesis of chalcones **1**–**9**. (i) 40% KOH, ethanol, room temperature or 50 °C, 24–48 h (yields: 6–30%); (ii) K_2_CO_3_, Ethanol, reflux 18 h, yields (30–89%).

**Scheme 4:**
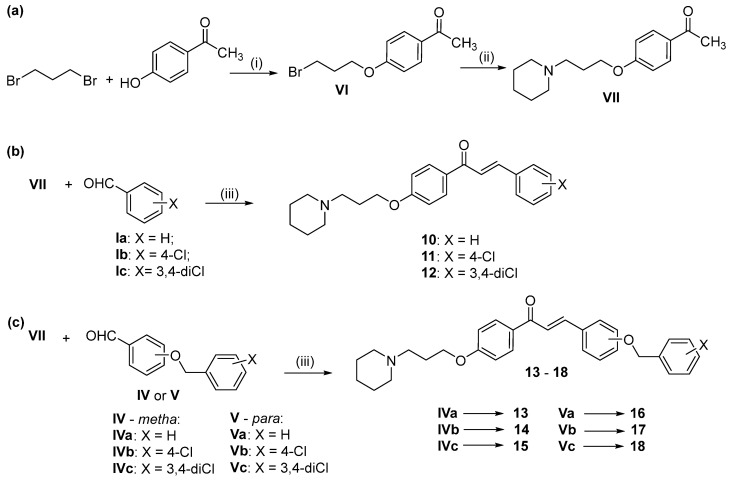
Synthesis of chalcones **10**–**18**. Reagents and conditions: (i) K_2_CO_3_, acetone, 48 h; reflux (32%); (ii) piperidine, K_2_CO_3_, KI, CH_3_CN, 24 h, reflux (29%); (iii) 15% KOH, C_2_H_5_OH, 0.5–4 h, room temperature (8–32%).

Compounds were synthesised as shown in Scheme 3 and Scheme 4. Chalcones **2**–**18** were obtained according to Claisen-Schmidt condensation from proper aldehydes **I**–**V** and 4-hydroxyacetophenone (chalcones **2**–**9**) or acyl derivative of piperidine **VII** (chalcones **10**–**18**). Non-commercially available aldehydes were prepared by *O*-alkylation of 3- (**IIa**) or 4-hydroxybenzaldehyde (**IIb**) with proper benzyl chlorides (IIIa, IIIb) in basic conditions. Compounds **VI** and **VII** were synthesised as described previously by Honkisz-Orzechowska et al. [28] or by slight modification. Final compounds (**2**–**18**) were purified by crystallisation from ethanol or by flash chromatography. All chalcones (except **11**) during synthesis were converted into hydrogen chloride. The final products were obtained with yields ranging from 6 to 32%. Differences in yield may be due to inadequately optimised reaction conditions that limited the progress of aldol condensation, such as insufficient reaction time or conducting the reaction at room temperature. Moreover, the purification step, particularly crystallisation, likely had a significant impact on the final yield due to the relatively high solubility of the compounds in ethanol.

The purity and identity of the compounds were confirmed by NMR spectroscopy (^1^H and ^13^C) and mass spectrometry. ^1^H NMR data proved that all chalcones are E isomers, which are more thermodynamically stable than the Z isomers. An α,β-unsaturated bond appeared as two doublets at about 7.6 and 7.9 ppm with coupling constant values of 15–16 Hz.

### 2.3. Preliminary In Vitro Pharmacological Studies of Compounds

#### 2.3.1. Human Histamine H_3_ Receptor Affinity

The affinity to hH_3_R was evaluated only for compounds **10**–**18** as compounds **1**–**9** lack the essential structural elements required for interaction with H_3_R, i.e. a moiety of basic character [15]. Compounds **10**–**18** were tested in a radioligand binding assay as described previously [28]. Results are shown in Table 1. All compounds had hH_3_R affinity with K_i_ values below 170 nM. The best affinity had compounds **10**–**12** (series 1) with K_i_ ≤ 30 nM, whereas the weakest had compounds **16**–**18** (89 nM ≤ K_i_ ≤ 195 nM) (series 3). There is no correlation between the introduction of chlorine/chlorines into the phenyl ring and the hH_3_R affinity. The highest hH_3_R affinity was shown by compounds **10** and **11** with a K_i_ of 17 nM. In the safinamide derivatives (**13**–**18**), the compounds had lower affinity for hH_3_R (K_i_ > 45 nM) compared with compounds **10**–**13**. There is a clear correlation between the attachment of the benzyloxy substituent, *meta* (**12**–**15**) or *para* (**16**–**18**), and hH_3_R affinity. Compounds with *meta* substitution (**12**–**15**) showed higher affinity than the corresponding analogues with *para* attachment (**16**–**18**). In this group, no visible relationship can be observed between the increase in affinity and the introduction of a chlorine atom/chlorine atoms into the benzyloxy substituent. Compound **15** had the highest affinity for hH_3_R, with a K_i_ value of 46.8 nM.

#### 2.3.2. Functional Characterisation in cAMP Accumulation Assay of Selected Compounds

The two most promising compounds from the tested series, i.e., compounds **12** and **15**, were selected for a test to confirm their activity profile toward the H_3_R. The intrinsic activity was evaluated in a 3′,5′-cyclic adenosine monophosphate (cAMP) accumulation assay in CHO-K1 cells stably transfected with the H_3_R. Compounds were tested both in agonist and antagonist modes. Neither compound showed activity in agonist mode (E_max_: 0% and 3%, respectively), whereas both tested in antagonist mode inhibited the activity of R-α-methyl histamine (H_3_R agonist). This effect, in a concentration-dependent manner, led to an increase in cAMP levels in the cells. The equilibrium dissociation constant (K_b_ ± SD) of compound **12** was 3.94 ± 0.85 nM, whereas for compound **15**, K_b_ was 34.20 ± 2.78 nM (Supplementary data).

#### 2.3.3. Surface Plasmon Resonance Microscopy Kinetic Studies of Selected Compounds

**Figure 2:**
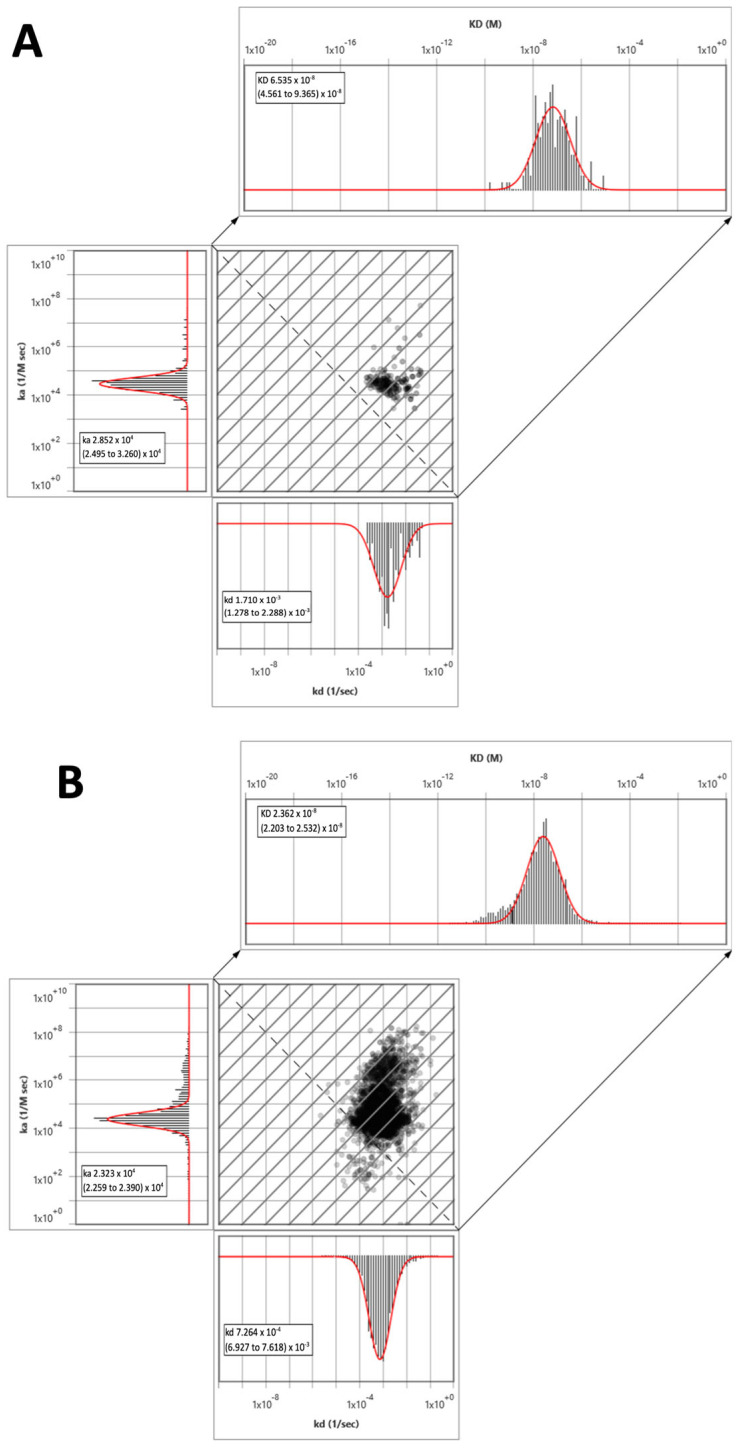
The Gaussian distributions (red curves) of the respective kinetic parameters (k_a_, k_d_, K_D_) for **DL76** (**A**) and pitolisant (**B**).

Kinetic characteristics can act as key factors distinguishing and predicting a drug’s effectiveness and safety. Traditionally, drug candidates were chosen for their strong ligand–protein binding affinity, but high affinity alone does not ensure clinical success. Research indicates that drug efficacy often correlates more closely with the dissociation rate than with equilibrium binding. Binding kinetics—how fast a drug binds and how long it stays bound—are now seen as equally important. The kinetics of binding can be investigated using the SPRM (Surface Plasmon Resonance Microscopy) method [29]. SPRM combines high-resolution optical microscopy with Surface Plasmon Resonance (SPR) technology [30]. It can directly measure both equilibrium binding affinity and kinetics of ligand–membrane protein interactions, such as ligand–GPCR binding. Additionally, SPRM is a label-free method, eliminating the need for radioactive tracers in experimental assays. Here, we report the first use of SPRM technology to examine H_3_R ligands, highlighting a novel approach in this field. Previously described studies have allowed the determination of binding kinetics parameters using fluorescence-based methods, such as calcium mobilisation [31], fluorescence polarisation [32] and NanoBRET assays [33]. For our studies, compound **15** was selected as the dual active ligand, while pitolisant (an approved H_3_R antagonist/inverse agonist) and **DL76** (our dual ligand) were included as reference compounds. Experiments were conducted on CHO-K1 cells overexpressing hH_3_R. Stock solutions (10 mM) of the tested compounds were prepared in DMSO, with final assay concentrations ranging from 0.5 nM to 10 µM. Despite repeated trials, no kinetic binding data were obtained for compound **15**. Analysis of the experimental workflow suggested that the lack of measurable response may be connected to the binding of compound **15** to bovine serum albumin (BSA), which was an essential component of the experimental conditions. Our assumptions were confirmed by LC-MS spectrum analysis and literature reports. Singh et al. [34] studied interactions of (*E*)-1-(2,4-dichlorophenyl)-3-(2-hydroxy-3-methoxyphenyl)prop-2-en-1-one with BSA. The study showed that this chalcone can bind to BSA without disturbing its natural structure or function. The interaction was found to be stable, as confirmed by NMR, high-resolution mass spectrometry, Fourier-transform infrared spectroscopy, UV spectroscopy, single-crystal X-ray diffraction, and computer analyses. For pitolisant and **DL76**, kinetic and equilibrium binding parameters were successfully determined (Table 2). Histograms of the Gaussian distributions (red curves) for the respective kinetic parameters (k_a_, k_d_, K_D_) of the tested compounds are shown in Figure 2. The equilibrium constant (K_D_) values obtained from the ligand binding assay and SPRM kinetics tests are comparable. For **DL76**, they are as follows: 39.2 ± 13.6 nM vs. 43.6 ± 12.6 nM, whereas for pitolisant: 15.0 ± 4.3 nM vs. 15.3 ± 4.7 nM, respectively. The residence time (RT), which reflects the duration for which a compound remains bound to its target, is comparable for **DL76** and pitolisant, with values of 16 min and 15.8 min, respectively. These results indicate comparable average interaction times of the compounds with the H_3_R.

In the comparative kinetics study of non-equilibrium Ca^2+^ mobilisation assays by Riddy et al. [31], pitolisant was classified among H_3_R antagonists as a “rapidly dissociating” ligand, with dissociation half-lives markedly shorter than those of the slowly dissociating class of compounds. Our SPRM measurements, however, yielded a dissociation rate constant k_off_ of 1.2 × 10^−3^ s^−1^, corresponding to a RT of ≈15.8 min, which is significantly longer than the values commonly attributed to the “fast-off” group. Our results are consistent with those reported by Mocking et al. [33], who reported an RT of approximately 17 min. The extended RT observed under various experimental conditions suggests that system-specific factors (e.g., surface immobilisation, low flow rates, receptor construct, rebinding) may affect dissociation relative to other assay formats. This discrepancy highlights the importance of assay context when comparing residence times across studies.

#### 2.3.4. Human MAO-B Inhibitory Activity

Pharmacological activity towards human MAO-B (hMAO-B) was evaluated in a spectrometric assay using Amplex Red, as previously described [21]. First, all compounds were tested at a concentration of 1 μM to assess their ability to inhibit MAO-B activity. Compounds that demonstrated more than 50% inhibition in two independent assays were further tested at different concentrations to determine IC_50_ values. Results are presented in Table 1 and show that simple chalcones **2**–**9** have hMAO-B inhibitory activity with IC_50_ below 340 nM. The most potent compounds had a 3,4-dichloro substituent in the phenyl ring, i.e., compounds **3**, **6** and **9**. The IC_50_ value of compound **3** was even below 1 nM (IC_50_ = 0.85 nM). Inhibitory activity of compounds **1** and **2** evaluated in our laboratory was higher than previously described by others (e.g., for **2**: data from [27]—IC_50_ = 31 nM vs. IC_50_ = 2.67 nM). Therefore, we repeated the experiments that confirmed our results. In the piperidinyl series (**10**–**18**), compounds showed weaker activity than the corresponding chalcones **2**–**9**. In this group, the inhibitory activity was higher in compounds with a safinamide motif (**13**–**18**). The same tendency was observed in all tested compounds (**1**–**18**), with the inhibitory activity increasing in the following order: unsubstituted ring < monochlorine substituted < dichlorine substituted. Among derivatives with the piperidinyl moiety (**10**–**18)**, the most potent were compounds with a *meta* substitution of a benzyloxy moiety (**13**–**15**). In this group, the compound **15** exhibited the highest inhibitory activity, with an IC_50_ of 212.5 nM; however, this activity was more than ten times weaker than that of simple chalcone **6** (IC_50_ = 22.4 nM).

#### 2.3.5. Reversibility of Human MAO-B

**Figure 3:**
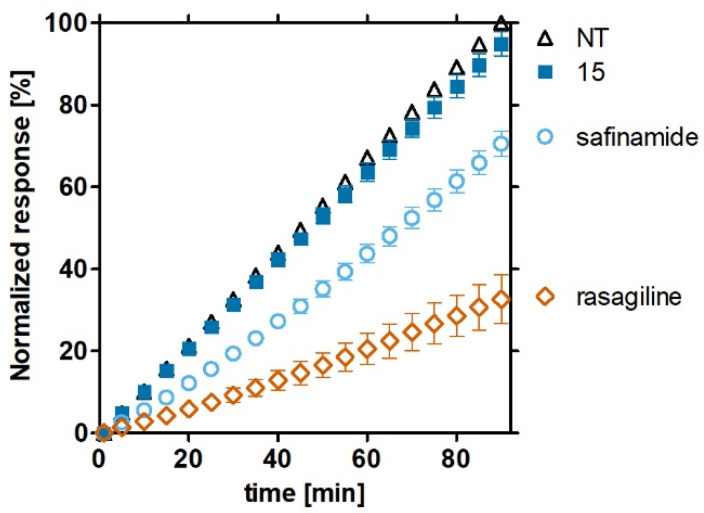
Reversibility of hMAO-B inhibition of compound **15**, safinamide and rasagiline measured using the rapid dilution method described by Copeland [35]. NT-non-treated, signal without inhibitors, only DMSO as solvent. References: rasagiline (irreversible inhibitor), safinamide (reversible inhibitor). Data represent mean values from 3 independent experiments (each in duplicates) with SEM.

Reversibility tests were conducted to confirm the binding mode with the enzyme. The most active dual ligand, compound **15**, was selected for this test. The test was performed using the rapid dilution method described by Copeland [35]. Safinamide (a reversible inhibitor) and rasagiline (an irreversible inhibitor) were used as reference substances. The enzyme was incubated with the tested inhibitors at a concentration corresponding to 10-fold the IC_50_ value for 30 min, then the samples were diluted using a high concentration of substrate (p-tyramine) in assay buffer. To observe restoration of MAO-B activity after the dilution fluorescence measurements were taken every 5 min for 1 h. The results are shown in Figure 3. Compound **15** showed reversible inhibition, i.e., the activity of the enzyme after the dilution in excess of the enzyme’s substrate was similar to that of the non-inhibited enzyme. The curves indirectly represent the amount of product produced by MAO-B after dilution and show the enzyme’s ability to restore its activity after inhibition. Curves of compound **15** and safinamide are comparable. The activity of hMAO-B was restored after dilution of safinamide and compound **15**, whereas rasagiline (an irreversible inhibitor), following the dilution of the inhibitor, was still able to inhibit most of the enzyme molecules, leading to a small amount of product released.

#### 2.3.6. Modality of Reversible Inhibition of Human MAO-B

**Figure 4:**
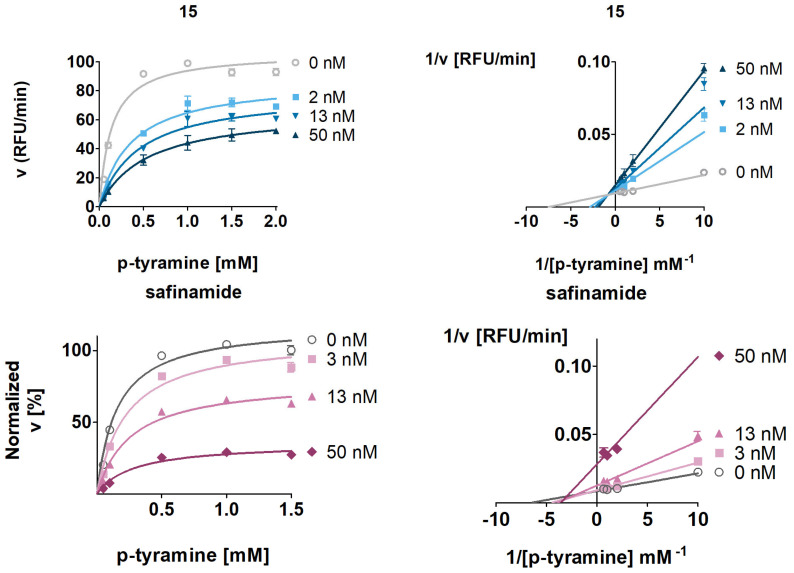
Michaelis–Menten (**left**) and Lineweaver–Burk (**right**) plots illustrating the mode of inhibition of the MAO-B enzyme by compound **15** and safinamide in the presence of p-tyramine as the substrate. In the Lineweaver–Burk plot, the point at which the lines intersect indicates the type of inhibition: competitive (on the *y*-axis) or mixed-competitive (to the left of the *y*-axis and above the *x*-axis).

Compound **15** was chosen to investigate its mode of inhibition. Three concentrations of the inhibitor (corresponding to its IC_20_, IC_50_, and IC_80_ values) were tested in conjunction with five substrate concentrations. As a substrate, p-tyramine was used at concentrations of 0.05, 0.1, 0.5, 1.0, 1.5 and 2.0 mM. Michaelis–Menten curves were fitted to the experimental data to obtain K_M_ and V_max_ values (Table 3). The data were then transformed into a double-reciprocal (Lineweaver–Burk) plot to illustrate the mode of inhibition more clearly (Figure 4).

The inhibition modality was proposed based on the analysis of changes in K_M_ and V_max_, as well as on fitting the experimental data to multiple inhibition models. The inhibitor represents either competitive or a variant of mixed inhibition, very close to competitive. A competitive inhibitor binds exclusively to the free enzyme, preventing substrate binding. Increasing the substrate concentration decreases inhibition due to the competitive nature of the interaction. The mixed mode of inhibition is a broader term that includes cases where the inhibitor binds to both the free enzyme and the enzyme–substrate complex. In the case of compound **15**, the mixed mode would be very close to competitive inhibition (sometimes referred to by some authors as mixed-competitive), meaning that the inhibitor shows affinity for both the free enzyme and the enzyme–substrate complex, but with a higher affinity for the free enzyme.

#### 2.3.7. Human MAO-A Inhibitory Activity

Piperidinyl chalcones (compounds **12**, **14**, **15**, **17** and **18**) with inhibitory activity for hMAO-B were further tested to evaluate their selectivity activity for human MAO-A (hMAO-A). MAO-A and MAO-B are isoenzymes that differ in location, substrate preference and significance in the therapy of different diseases. Selective isoforms are considered safer for treatment [36]. Compounds were screened at a concentration of 10 μM in two independent experiments. The results (mean values) are shown in Table 1. None of them showed inhibition of hMAO-A higher than 25%, and they were not further tested.

### 2.4. In Silico Docking Studies

#### 2.4.1. Docking Studies to Histamine H_3_ Receptor

**Figure 5:**
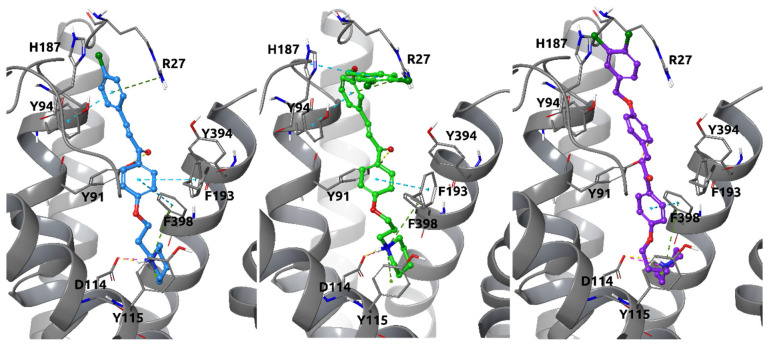
Predicted binding mode of compounds: **11** (blue), **15** (green) and **18** (violet) within the H_3_R binding site. Hydrogen bonds are shown with yellow dashed lines, salt bridges with magenta lines, cation–π interactions with green lines, and π–π interactions with blue lines. The protein is shown in grey cartoon representation, with interacting amino acid residues depicted as sticks; the ligands are displayed in ball-and-stick representation.

Published in 2022, the H_3_R structure in complex with a H_3_R antagonist (PF-03654746; PDB ID: 7F61 [37]), enabled validation of previously proposed ligand-receptor interactions and offered deeper insights into the receptor’s functional mechanisms. Based on these findings, compounds **11**, **15** and **18** were selected for molecular docking studies to investigate how their structural differences might relate to the observed biological activity. The compounds were characterised by favourable MMGB–SA (Molecular Mechanics–Generalized Born Surface Area) binding free energies (ΔG_bind = −95.51, −99.69, and −78.51 kcal/mol, respectively) and moderate yet comparable ligand efficiencies derived from docking scores (glide LE = −0.294, −0.281, and −0.274, respectively). All ligands occupied the H_3_R binding pocket in a similar manner, preserving the key antagonist/inverse agonist interaction, namely salt bridge and/or hydrogen bond formation between protonated amine nitrogen and ASP114^3.32^ (superscripts denote Ballesteros–Weinstein numbering, as presented in GPCRdb [38]). Additional stabilisation of the basic part of the molecules was conducted through cation–π interactions with caging Y115^3.33^ and F398^7.39^. On top of that, compounds **11** and **15** formed a donor interaction via Y91^2.61,^ indicating a robust polar interaction network as well as π–cation contacts with R27^1.39^, located near the extracellular face of the receptor, suggesting a possible role in initial ligand recognition or orientation. In contrast, the slightly different calculated orientation of compound **18** in the binding pocket did not allow for explicit hydrogen bond donor or acceptor interactions, suggesting reduced stabilisation through polar contacts (Figure 5).

Aromatic and hydrophobic interactions were broadly conserved across all three ligands, primarily involving Y91^2.61^, Y94^2.64^, W110^3.28^, Y115^3.33^, Y189(EL2), F193(EL2), Y374^6.51^, Y394^7.35^, F398^7.39^, and W402^7.43^, consistent with the π-rich character of the orthosteric site. However, compound **15** itself established additional contact with Y194(EL2), expanding its aromatic interface relative to compounds **11** and **18**. Notably, compound **18** did not exhibit interaction with Y94^2.64^. This absence may be attributed to the *para*-substitution of the proximal 3,4-dichlorobenzyl moiety in compound **18**, which orients the aromatic ring more distally from the core binding pocket. In comparison, the *meta*-substitution pattern observed in compound **15** appears to promote a more optimal spatial alignment with Y94^2.64^, thereby enabling favourable π–π stacking or edge-to-face aromatic interactions. This structural difference may lead to weaker hydrophobic stabilisation in compound **18**, which could partly explain its distinct interaction profile and slightly lower H_3_R affinity.

The stability of the calculated poses was further evaluated using 250 ns molecular dynamics (MD) simulations. All data used for the analysis, as described in the paragraphs below (for both H_3_R and MAO-B), are provided in the Supplementary data.

**Figure 6:**
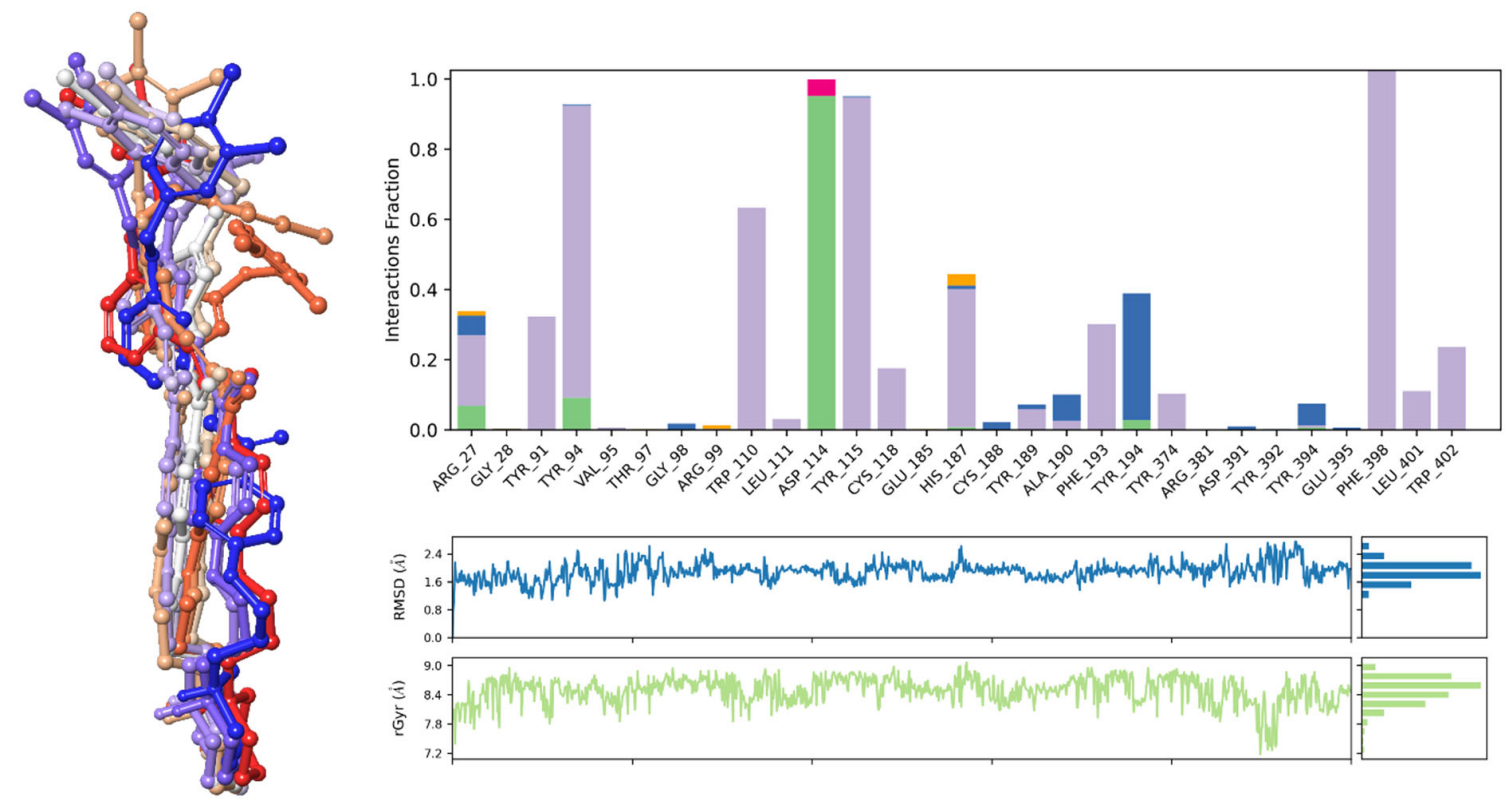
(**Left panel**): Orientation of compound **15** in the H_3_R binding pocket during the 250 ns MD simulation. Different colours represent distinct frames: 0 ns is shown in blue, transitioning through the violet spectrum (dark to light: 25–100 ns) to grey (125 ns) and vice versa through the orange spectrum (light to dark: 150–225 ns) to red (250 ns). (**Right panel**, top-to-bottom): Protein-ligand contacts histogram (green for hydrogen bonds, violet for hydrophobic contacts, blue for water bridges; *X*-axis represents interaction fraction (1.0 = 100% simulation time), and *Y*-axis represents particular interacting amino acids; Time evolution of Root Mean Square Deviation (RMSD) for ligand (blue) and radius of Gyration (rGyr, green) for specific frames relative to the reference frame at 0 ns.

In the recorded trajectory of the **15**-H_3_R complex, the ligand maintained a generally stable binding pose, evidenced by stable interactions and favourable trends in calculated binding energy. Throughout the trajectory, D114^3.32^ remained a persistent polar interaction partner, present in over 95% of simulation frames, with additional stabilisation contributed by cation–π interactions involving Y115^3.33^ and F398^7.39^. An initial hydrogen bond donor interaction from Y91^2.61^ was also observed; however, the role of this residue shifted toward supporting the ligand’s first aromatic ring via π–π stacking and aromatic hydrogen bonding between the distal, extracellular-facing substituted aromatic ring of compound **15** and residues R27 and H187 (EL2) were transiently observed, suggesting a dynamic but non-essential contribution from these residues. At the same time, its surface exposure and flexibility may enhance binding entropy (Figure 6).

The binding free energy (expressed by ΔG Bind values) remained consistently favourable across trajectory snapshots, averaging approximately −155.5 kcal/mol during equilibrated frames, with only minor variation observed in early frames before system relaxation. Detailed Prime energy values for each simulated complex, including the corresponding MM–GBSA ligand efficiencies, are available in the Supplementary data. Hydrophobic interactions constituted the primary energetic contribution, complemented by favourable hydrogen bonding and packing interactions, which together support the thermodynamic feasibility of high-affinity binding under membrane-mimicking conditions. Root Mean Square Deviation (RMSD) analysis revealed moderate ligand fluctuation relative to the starting pose, followed by convergence, indicating no significant displacement from the orthosteric pocket. Residues forming the aromatic cage—Y94^2.64^, W110^3.28^, Y115^3.33^, and Y374^6.51^—maintained stable contact distances throughout the simulation, underscoring the role of π–π and hydrophobic interactions in anchoring the ligand. Across the three simulated ligand–receptor complexes, several shared and divergent interaction patterns were observed, highlighting the structural determinants of ligand stability and binding efficiency in the H_3_R orthosteric pocket. All three ligands maintained stable polar anchoring via D114^3.32^ and hydrophobic interactions with conserved aromatic residues. However, differences emerged in how each compound engaged the extracellular vestibule through its substituted aromatic extensions. Similar to compound **15**, compound **11** occupied a well-defined cleft between TM2 and TM7, forming stable π–π interactions with Y91^2.61^ and Y94^2.64^, as well as with F398^7.39^ and W402^7.43^. This conformation contributed to consistently calculated binding-free energy profiles and low RMSD fluctuations, indicating a high degree of structural and energetic stability. In contrast, in compound **18** the *para*-substituted 3,4-dichlorobenzyl group of compound **18**, promoted the position of the distal ring farther from TM2, limiting interactions with Y94^2.64^ and redirecting engagement toward TM7, where the contacts were observed to be more transient. This repositioning corresponded with increased RMSD variability and more fluctuant ΔG Bind values across frames, indicating a less constrained binding mode. The highest-energy state corresponds to reduced π-stacking and the loss of polar contacts. RMSD analysis revealed greater structural deviation and delayed convergence compared to compounds **11** and **15**, consistent with a more flexible or less tightly bound ligand conformation. Thus, while all three ligands exploit a shared pharmacophoric core, the geometry and dynamics of the distal fragment strongly influence binding stability and entropic behaviour, and might contribute to differences in receptor interactions.

Molecular dynamics simulations of compounds **11**, **15**, and **18** were evaluated relative to the reference histamine H_3_ receptor antagonist PF03654746 co-resolved within the H_3_R complex [37]. The analysis of the native ligand simulation revealed a rigid, thermodynamically favourable ligand pose anchored by a stable ionic interaction with D114^3.32^, π–cation engagement with Y115^3.33^ and C118^3.36^, and aromatic stacking with Y91^2.61^ and F398^7.39^. RMSD remained at low values, and the ligand maintained minimal internal strain across the trajectory, confirming its suitability as a reference conformation for antagonist binding. All three ligands in question retained the key ionic anchor with D114^3.32^ and engaged conserved aromatic cage residues (Y91^2.61^_,_ Y115^3.33^, F398^7.39^) through π–π or cation–π interactions, sharing the core pharmacophoric anchor with the PF03654746 ligand, but differed in distal orientation, receptor engagement patterns, and dynamic stability. Compound **15** most closely reproduced the reference pose, exhibiting stable binding energy and extended polar interactions at the extracellular face. Compound **11** showed a similarly stable, though more compact, pose with high ligand efficiency. In contrast, compound **18**, due to its *para*-substituted dichlorobenzyl group, deviated from the optimal alignment, losing interaction with Y94^2.64^ and displaying increased RMSD fluctuations and less stable binding, consistent with a more flexible and less tightly bound profile.

Last but not least, H_3_R inactive state interaction in the D^3.49^-R^3.50^-F^3.51^ motif, with the salt bridge and/or H-bond formed between D131^3.49^ and R132^3.50^ and the postulated H_3_R inactive state 3–7 lock between D114^3.32^ and W402^7.43^ [39], was maintained for the majority of the simulation time, which may give additional confirmation of the presumed stabilisation of the inactive state of the receptor by the tested ligands.

#### 2.4.2. Docking Studies to Human MAO-B

**Figure 7:**
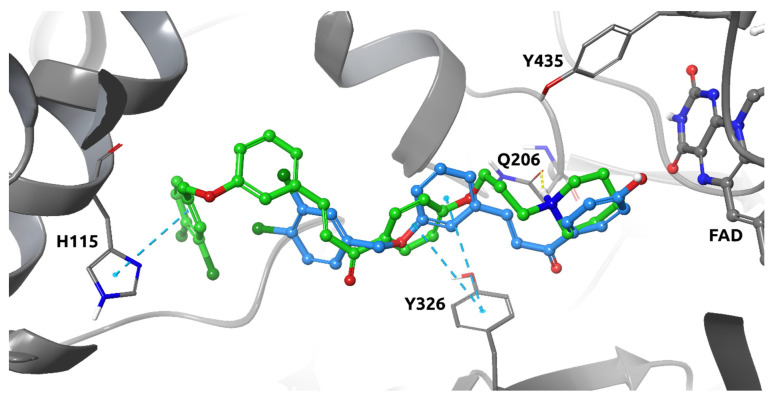
Predicted binding mode of compound **15** (green) and compound **6** (blue) within the MAO-B active site. Hydrogen bonds are shown with yellow dashed lines and π–π interactions with blue lines. The protein is presented in a grey cartoon, with interacting amino acid residues depicted as sticks; the ligands are displayed in ball-and-stick representation.

An additional in silico analysis with a possible explanation for the hMAO-B inhibition of compound **15** has also been performed, using the well-described 2V5Z structure [18]. The active site comprises a characteristic aromatic cage, formed by Y398 and Y435, which stabilises ligands through π–π stacking, adjacent to a polar cavity lined with Q206 and structured water molecules that facilitate hydrogen bonding. The flavin adenine dinucleotide (FAD) cofactor, located at the bottom of the pocket, constitutes the redox centre and contributes to the pocket’s conformation and polarity. Throughout the recorded trajectory, compound **15** formed consistent interactions with key aromatic cage residues identified in the 2V5Z structure, including π–π stacking with Y398 and Y435 and hydrogen bonding to the polar cavity Q206. The distal moiety of compound **15** occupied the substrate cavity and engaged the FAD cofactor region transiently (Figure 7). Calculated binding free energy averaged approximately ΔG Bind of −232.5 kcal/mol (min −263.8, max −203.8) with low frame-to-frame fluctuation, indicative of relatively stable binding. RMSD analysis showed modest deviations, further confirming structural stability during the simulation.

For comparative purposes, compound **6**, a simple chalcone precursor of compound **15**, which displays a high hMAO-B inhibition with an IC_50_ of 20.6 nM, has also been used. Compound **6** lacks the piperidinylpropoxy motif and, consequently, features an unsubstituted 4-hydroxyphenyl group, introducing the potential for additional hydrogen bonding with the polar cavity and surrounding water molecules. This modification shifts the balance between hydrophobic and polar interactions within the binding site, accounting for the observed differences in potency and dynamic behaviour between the two compounds. Compound **6** exhibited notable differences in binding behaviour within the MAO-B active site. While both ligands maintained core interactions, compound **6** displayed stronger and more persistent engagement of the distal pocket and FAD region, exhibiting more sustained π–π stacking with aromatic cage residues and water-mediated bridges.

**Figure 8:**
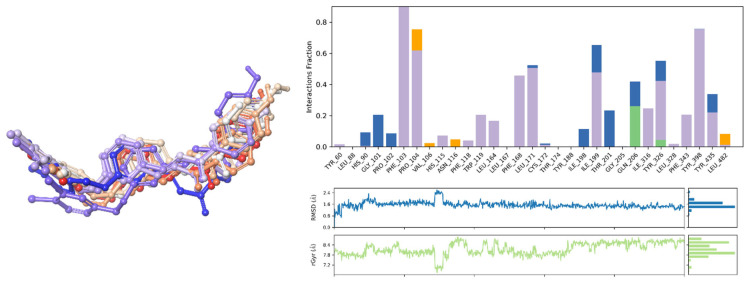
Visualisation of the **15**-MAO-B complex dynamics simulations using the same approach as for the H_3_R receptor. Protein–ligand contacts histogram (green for hydrogen bonds, violet for hydrophobic contacts, blue for water bridges). *X*-axis represents interaction fraction (1.0 = 100% simulation time), and *Y*-axis represents particular interacting amino acids. Time evolution of RMSD for ligand (blue) and radius of Gyration (rGyr, green) for specific frames relative to the reference frame at 0 ns.

Under the simulation conditions, both ligands stabilised quickly, with the protein RMSD levelling off at approximately ~1.5 Å and the ligand RMSD remaining low across the recorded trajectories, reflecting stable complex formation. Notably, compound **6** exhibited a lower average ligand RMSD and a narrower fluctuation range compared to compound **15** (Figure 8), suggesting tighter anchoring and reduced conformational mobility. Root-mean-square fluctuation (RMSF) analysis confirmed that both ligands maintained the rigidity of the aromatic cage, but compound **6** induced slightly lower flexibility in adjacent loop regions. Consistent with its hydroxyl-substituted aromatic ring, halogen contacts were diminished in compound **6**, and the ligand torsion profiles revealed a more restricted conformation with limited rotational flexibility compared to compound **15**. However, the calculated binding free energy of compound **6** was less negative than that of compound **15**, averaging ΔG Bind of –187.6 kcal/mol (min = −199.9, max = −174.9). Together, these findings demonstrate that the structural specificity of compound **6** enhances its complementarity and enthalpic stabilisation within the MAO-B active site, accounting for its improved binding affinity and inhibitory activity.

To understand the structural determinants of MAO-B inhibition for compounds **6** and **15**, their molecular dynamics trajectories were evaluated in direct comparison with the native binding mode of the co-crystallised ligand Safinamide (SAF) of the 2V5Z structure. Both ligands preserved the core SAF pharmacophore, including π–π stacking with Y398 and Y435 and polar interactions with Q206 near the FAD cofactor. These conserved contacts were maintained throughout 250 ns molecular dynamics simulations, indicating stable anchoring of the ligands within the substrate cavity. Compound **15** mimicked the SAF pose while adding pharmacophoric bulk without compromising anchoring; however, it extended beyond the substrate cavity via its piperidinylpropoxy moiety, maintaining favourable binding energy and interaction coverage. This extension introduced moderate conformational flexibility, reflected in slightly elevated ligand RMSD and broader torsional distributions, yet the average MM–GBSA binding energy remained strongly favourable (−232.5 kcal/mol), exceeding that of SAF. In contrast, compound **6** adopted a more compact, deeply inserted pose with improved polar complementarity and lower RMSD. Although π–π stacking with Y398 and Y435 remained consistent, the absence of halogen substitution reduced hydrophobic packing, and torsional profiles showed a more conformationally restricted ligand.

### 2.5. Preliminary In Vitro ADMET Studies of Selected Compounds

Two compounds, **12** and **15**, the most potent dual-target ligands, were selected for preliminary ADMET (Absorption, Distribution, Metabolism, Excretion, Toxicity) studies. Both compounds were evaluated in complementary in vitro assays to characterise their permeability, microsomal stability, CYP inhibition potential, and cellular toxicity.

#### 2.5.1. Permeability Evaluation

First, an absorption study was performed using the Parallel Artificial Membrane Permeability Assay (PAMPA), which is used to determine the ability of molecules to passively diffuse through biological membranes [40]. Both compounds precipitated under used assay conditions (100 μM solution of compounds; PBS, pH 7.4, 20% methanol, 5 h), leaving no detectable analyte in acceptor compartments as well as no (compound **15**) or trace amount (compound **12**) in donor ones. Membrane adsorption was also considered, preventing a reliable determination of *P_e_* values. These results indicate poor solubility and/or instability in the PAMPA setup, suggesting limited passive diffusion potential. Therefore, the PAMPA test appeared to be insufficient for determining the permeability parameter of these compounds; thus, an assay with Caco-2 cells was used. The Caco-2 cell line, originating from human colorectal carcinoma, is widely employed as an in vitro model to predict intestinal drug absorption [41]. The results of the studies are summarised in Table 4 and expressed as the apparent permeability coefficient (P_app_), which indicates the potential of a drug candidate to be absorbed across the human intestinal epithelium, and as the efflux ratio (ER), which provides insight into the mechanisms of drug transport across biological membranes. Caffeine was used as the well-permeable reference (P_app_(A→B) = 30.16 ± 2.94; P_app_(B→A) = 34.80 ± 10.88 × 10^−6^ cm/s). Both tested compounds (**12** and **15**) showed very low permeability with ERs below unity, indicating no active efflux involvement. Compound **12** exhibited P_app_(A→B) = 0.75 ± 0.05 and P_app_(B→A) = 0.52 ± 0.03 × 10^−6^ cm/s (ER ≈ 0.70), while compound **15** showed P_app_(A→B) = 0.14 ± 0.02 and P_app_(B→A) = 0.06 ± 0.03 × 10^−6^ cm/s (ER ≈ 0.43).

#### 2.5.2. Metabolic Stability

The metabolic stability studies in vitro were conducted using rat liver microsomes (RLMs) for compounds **12** and **15**. Results are shown in Table 5. Incubation with RLMs (2 h) resulted in the appearance of new chromatographic peaks absent in controls lacking microsomes, confirming the metabolic transformation of both compounds. UPLC-MS analyses showed the formation of the main metabolite as a product of hydroxylation (*m*/*z* + 16). For both compounds, the retention times of the product and the starting substance were very similar, and in the case of compound **15**, they coincided exactly. Hydroxylation most likely occurs in the piperidine ring or propyl chain. Such sites of metabolism are suggested by the MetaSite 6.0.1 in silico tool (Supplementary data).

For compound **15** in mouse liver microsomes (MLMs), pharmacokinetic parameters were determined experimentally, i.e., intrinsic clearance (Cl_int_) and half-life (t_0.5_). The results were compared to the unstable drug Verapamil and are summarised in Table 6.

Compound **15** displayed weak metabolic stability, similar to the reference unstable drug Verapamil. The calculated parameters t_0.5_ and Cl_int_ for compound **15** were 18.99 min and 287.75 mL/min/kg, respectively, while for Verapamil, they were 22.60 min and 239.50 mL/min/kg.

**Figure 9:**
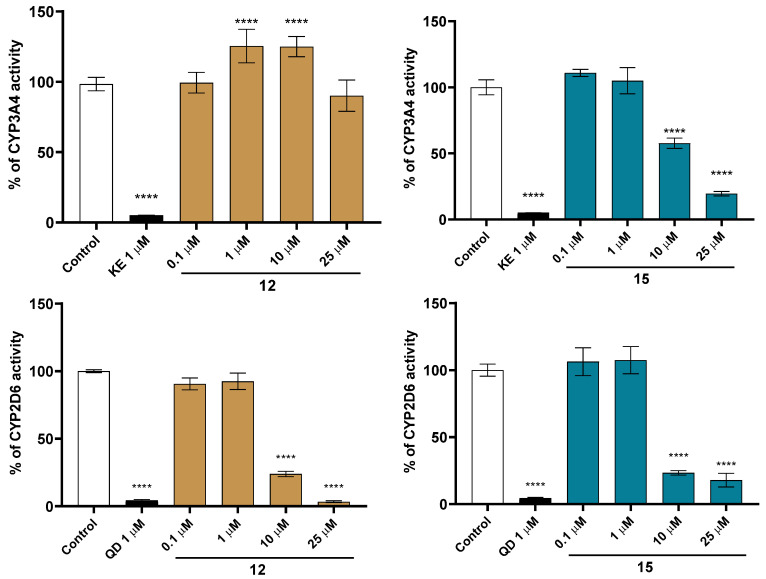
The influence of compounds **12** and **15** on CYP3A4 (**left**) and CYP2D6 (**right**) at the concentration range 0.1–25 µM. Statistical significance (**** *p* < 0.0001) was analysed by Graph Pad Prism 8.0.1 software using One-way ANOVA and Bonferroni’s Multiple Comparison Post-Test. KE = reference inhibitor ketoconazole, QD = reference inhibitor quinidine.

Compounds **12** and **15** were also tested for their influence on two major cytochrome (CYP) P450 isoforms (CYP3A4 and CYP2D6) at concentrations ranging from 0.1 to 25 µM. The results are shown in Figure 9. Each compound exhibited a slightly different behaviour: compound **12** caused a slight activation of CYP3A4 at concentrations of 1 µM and 10 µM, whereas compound **15** inhibited CYP3A4 activity at 10 µM. In the case of CYP2D6, both compounds inhibited enzyme activity by approximately 70% at a concentration of 10 µM. The selective reference inhibitors ketoconazole and quinidine almost completely inhibited their respective CYP isoforms at a concentration of 1 µM (Figure 9).

#### 2.5.3. Preliminary Cell Toxicity

**Figure 10:**
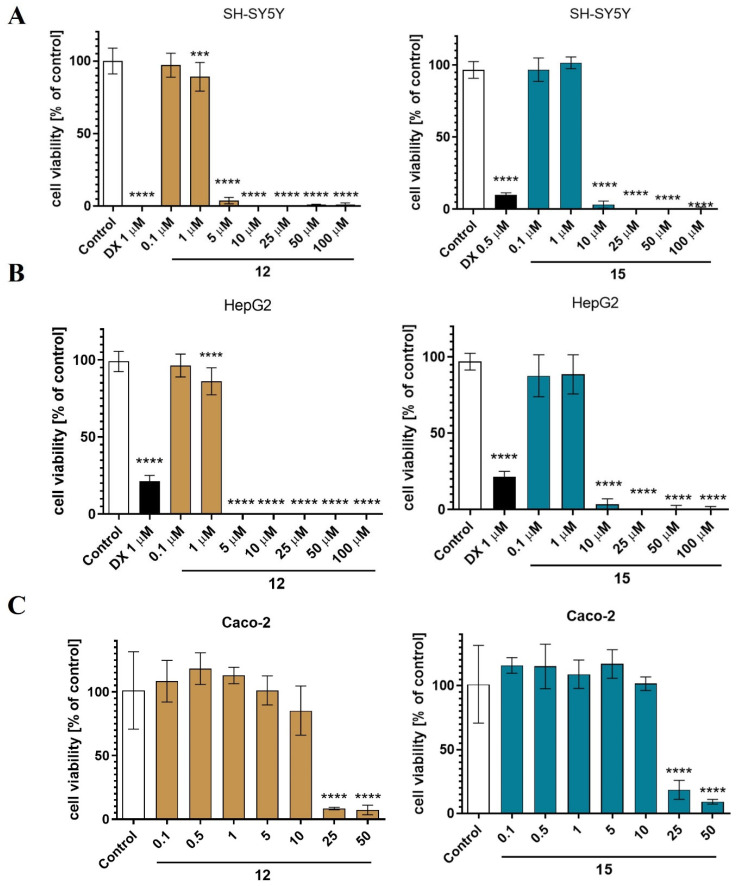
The effects of compounds **12** and **15** on the viability of SH-SY5Y (**A**), HepG2 (**B**), and Caco-2 (**C**) cells were assessed after incubation for 48 h (Caco-2) or 72 h (SH-SY5Y and HepG2) at concentrations ranging from 0.1 to 100 µM. Statistical significance (*** *p* < 0.001, **** *p* < 0.0001) was determined using GraphPad Prism 8.0.1 software with one-way ANOVA followed by Bonferroni’s multiple comparisons post hoc test. DX denotes the reference cytostatic drug doxorubicin.

The toxicity of compounds **12** and **15** was evaluated in three cell lines: SH-SY5Y (human neuroblastoma cell line), HepG2 (human liver cancer cell line) and Caco2 (human colorectal cancer cell line). Both compounds exhibited hepatotoxicity and neurotoxicity. Neurotoxicity was observed at 5 µM for compound **12** and at 10 µM for compound **15** (Figure 10A). Compound **12** induced complete death of HepG2 cells at a concentration of 5 µM, whereas compound **15** caused the same effect only at 25 µM (Figure 10B). The compounds showed the lowest toxicity in the Caco-2 cell line; although some toxicity was observed at 25 µM, it did not result in complete cell death (Figure 10C). Furthermore, microscopy images of neuroblastoma and HepG2 cells revealed compound precipitation at concentrations of 25–100 µM for compound 12 and at 50 and 100 µM for compound **15** (Supplementary data).

### 2.6. Activity Profile of Compound ***15***—In Vitro Studies

#### 2.6.1. Effect of Compound **15** on Human Peripheral Blood Mononuclear Cell (PBMC) Viability

**Figure 11:**
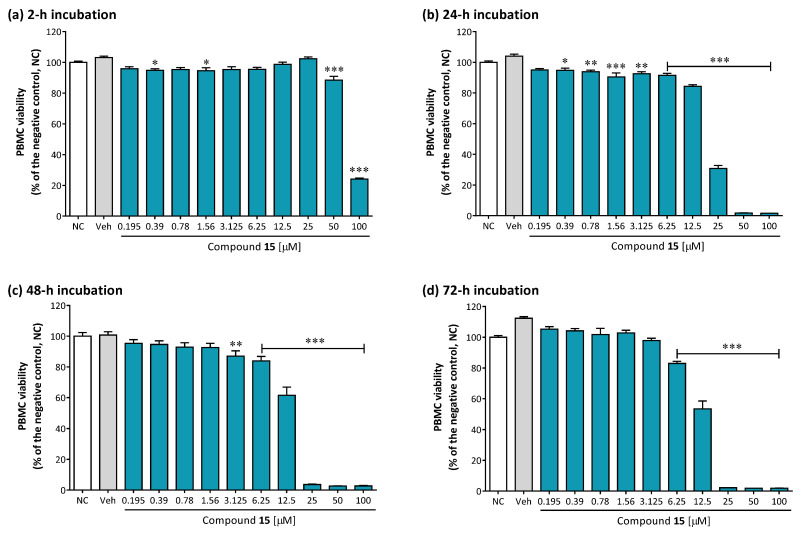
Effect of Compound **15** on PBMC viability at different incubation times: 2 h (**a**), 24 h (**b**), 48 h (**c**) and 72 h (**d**). The viability of individual samples was calculated relative to the negative control (NC, untreated cells). The positive control consisted of PBMCs incubated with 0.1% DMSO (Veh). Bars represent the means with SEM from 4 independent experiments and are expressed as a percentage of untreated control cells. One-way ANOVA and Dunnett’s multiple comparisons test: * *p* < 0.05, ** *p* < 0.01 and *** *p* < 0.001 vs. Negative control (NC).

In the subsequent step, we examined the effect of compound **15** on the viability of human PBMCs. The results are shown in Figure 11 and Table 7 (left column).

The cytotoxic effect of compound **15** on PBMCs depends on its concentration and incubation time. According to the data obtained, a 2 h incubation at concentrations ranging from 0.195 to 25 µM did not induce cytotoxicity (Figure 11a). For the two highest concentrations (50 and 100 µM), a statistically significant reduction in PBMCs compared to the negative control (NC) was observed (One-way ANOVA and Dunnett’s multiple comparisons test, *p* < 0.001), although at 50 µM, it did not exceed 12% (Figure 11a). At longer incubation times (24 h, 48 h, and 72 h), a significant decrease in PBMC viability compared to the control was observed at 6.25 μM and above, as shown by One-way ANOVA and Dunnett’s multiple comparisons test (*p* < 0.001). Notably, the highest concentrations of compound **15** (50 and 100 μM) reduced PBMC number by over 98% (Figure 11b–d). Additionally, during 48 h and 72 h incubation, treating cells with 25 μM of compound **15** produced a similar reduction.

In summary, the resazurin reduction assay on PBMCs demonstrated the toxicity of compound **15** within the micromolar range, as indicated by the calculated IC_50_ values (Table 7, left column). The IC_50_ values for compound **15** against PBMCs range from 11.97 to 80.30 μM and decrease with incubation time.

#### 2.6.2. Effect of Compound **15** on the Viability and Proliferation of the Human Neuroblastoma Cell Line (SH-SY5Y)

**Figure 12:**
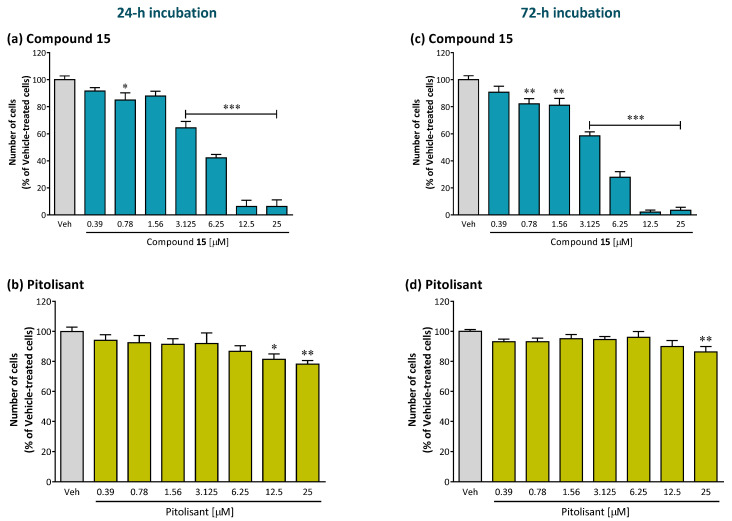
The effects of Compound **15** (**a**,**c**) and pitolisant (**b**,**d**), used as a reference drug, on the growth rate of SH-SY5Y after 24 h (**a**,**b**) and 72 h (**c**,**d**) incubation. The growth rate for individual samples was presented relative to the vehicle-treated cells (0.1% DMSO). Cell numbers were determined using image cytometry and were calculated as the sum of Hoechst-33342-stained cell nuclei counted in 16 microscopic fields per well (4 replicates per treatment) and expressed as a percentage of the vehicle-treated control (Veh). Bars represent the means with SEM of 3 independent experiments, each consisting of 6 replicates per treatment group. One-way ANOVA with post hoc Dunnett’s test: * *p* < 0.05; ** *p* < 0.01; *** *p* < 0.001 versus Veh (vehicle-treated cells).

The SH-SY5Y cells were also cultured with compound **15** at seven different concentrations ranging from 0.39 to 25 μM for 24 and 72 h. Pitolisant, the H_3_R antagonist/inverse agonist, was used as a reference drug. The results showed that compound **15**, only at the lowest concentration used (0.39 μM), did not affect cell viability (Figure 12a,c). The study revealed a significant decrease at *p* < 0.001 in cell number starting from a concentration of 3.125 μM. The most notable reduction in neuroblastoma viability (over 90% compared to control cells) at both incubation times was observed at concentrations of 12.5 and 25 μM. The IC_50_ values for compound **15** against SH-SY5Y cells, depending on incubation time, are 4.46 and 3.44 μM (Table 7, right column).

Pitolisant—unlike compound **15**—showed only a minor effect on neuroblastoma cells, causing a maximum decrease in their viability of about 20% at the two highest concentrations: 12.5 and 25 μM (Figure 12b,d). The viability of SH-SY5Y cells, assessed by IC_50_ values after 24 h and 72 h treatment with pitolisant, is 425 and 64.97 μM, respectively.

**Figure 13:**
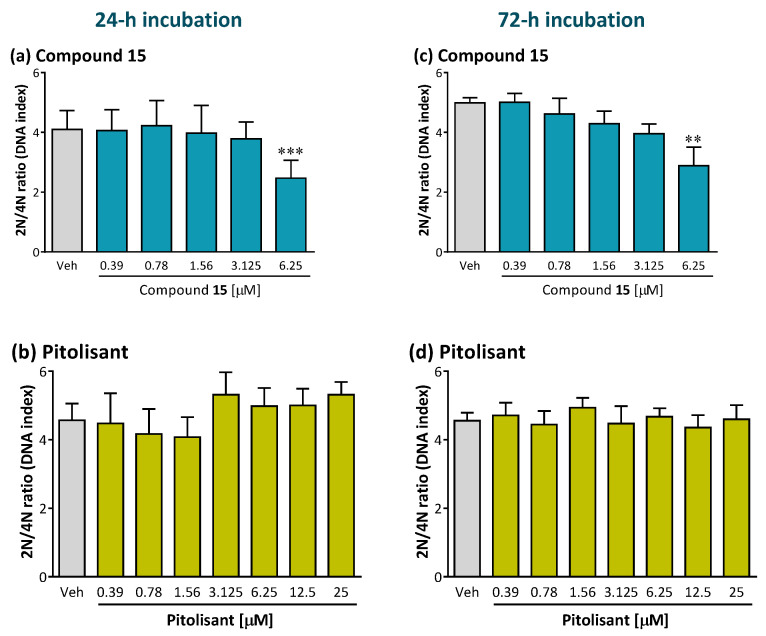
The effects of compound **15** (**a**,**c**) and pitolisant (**b**,**d**), used as a reference drug, on the neuroblastoma SH-SY5Y 2N/4N DNA ratio. SH-SY5Y cells were treated with either vehicle (0.1% DMSO) or increasing concentrations of compound **15** for 24 and 72 h. Cellular DNA content was determined using image cytometry, based on Hoechst-33342 fluorescence intensity of individual cellular nuclei according to the method described elsewhere [42]. The analysis was performed with the Cell Cycle Bioapplication V3, and the results were expressed as a DNA 2N/4N ratio. This involved analysing the fluorescence of nuclei in 500 cells across four replicates per experiment. Each value represents the mean ± SEM from at least 3 independent experiments. One-way ANOVA and Dunnett’s multiple comparisons test: ** *p* < 0.01, or *** *p* < 0.001 versus control counterparts (Veh).

Besides cell viability, the growth patterns of SH-SY5Y cells in the presence of compound **15** were also examined. As in previous experiments, neuroblastoma cells were treated with increasing concentrations of the tested compound (0.39–6.25 µM) for 24 or 72 h. Using image cytometry, the DNA content index was calculated as the 2N/4N DNA ratio to analyse cell cycle distribution. In this study, pitolisant was also utilised as a reference compound (concentration range: 0.39–25 µM). Figure 13a,c demonstrate that incubation with compound **15** at a concentration of 6.25 µM caused cell cycle arrest in the G2/M (4N) phase, as evidenced by a statistically significant decrease in the 2N/4N DNA ratio compared to cells treated with the vehicle. The inhibitory effect of compound **15** on cell cycle progression directly reduced the number of SH-SY5Y cells, as shown in complementary experiments (Figure 13a,c). Pitolisant, at the concentrations used for 24 h and 72 h, did not affect the cell cycle (Figure 13b,d).

**Figure 14:**
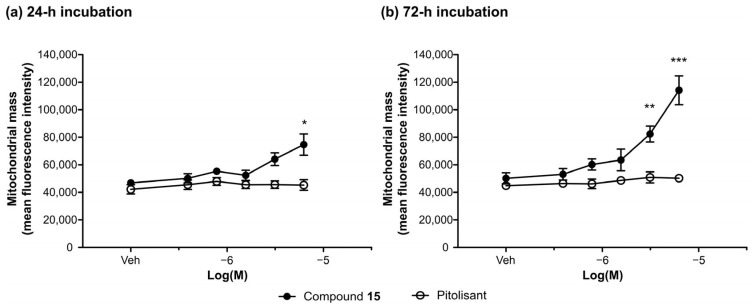
Compound **15** disrupts mitochondrial homeostasis in SH-SY5Y cells. SH-SY5Y cells were incubated with increasing concentrations of compound **15** or pitolisant for 24 and 72 h, followed by a short (30 min) incubation in the presence of fluorescent probe MitoTracker Orange CMTMRos that accumulates in active mitochondria. After acquisition of images via the ArrayScan VTI HCS Reader, single cells were analysed with Cell Health Profiling Bioapplication V3 software (500 cells/well), and mean total fluorescence intensity was calculated for each well (4 replicates per experiment). Each point indicates the mean ± SEM from at least 3 independent experiments. One-way ANOVA and Dunnett’s multiple comparisons test: * *p* < 0.05, ** *p* < 0.01, or *** *p* < 0.001 versus control counterparts (Veh).

Figure 14 shows changes in mitochondrial mass in SH-SY5Y cells (measured as mean total fluorescence intensity) incubated with compound **15** or pitolisant at concentrations of 0.39, 0.78, 1.56, 3.125, and 6.25 µM for 24 and 72 h. Treatment of SH-SY5Y cells with compound **15** for either 24 or 72 h led to an increase in mitochondrial biogenesis, probably as an adaptive response to metabolic demands and cellular stress. These effects were significant and noticeable at 3.125–6.25 µM of compound **15** compared to untreated cells. No changes in mitochondrial mass were observed following incubation with the same concentrations of pitolisant.

#### 2.6.3. The Genotoxic Potential of Compound **15**—Results from the Comet Assay

**Figure 15:**
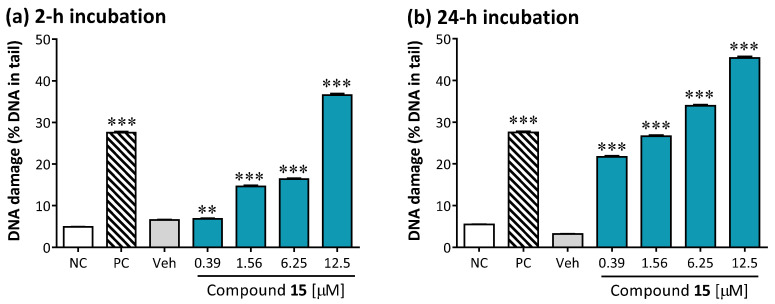
DNA damage in human peripheral blood mononuclear cells (PBMCs) incubated for 2 h (**a**) and 24 h (**b**) at 37 °C with Compound **15** (0.39–12.5 µM), analysed by the alkaline comet assay. The negative controls (NC) consisted of PBMCs incubated only with cell culture medium at 37 °C. The positive controls (PC) were PBMCs incubated with 20 µM H_2_O_2_ for 15 min on ice. The bars represent the mean results ± SEM (*n* = 100). One-way ANOVA and Dunnett’s multiple comparisons test: ** *p* < 0.01 and *** *p* < 0.001 vs. Negative control (NC).

**Figure 16:**
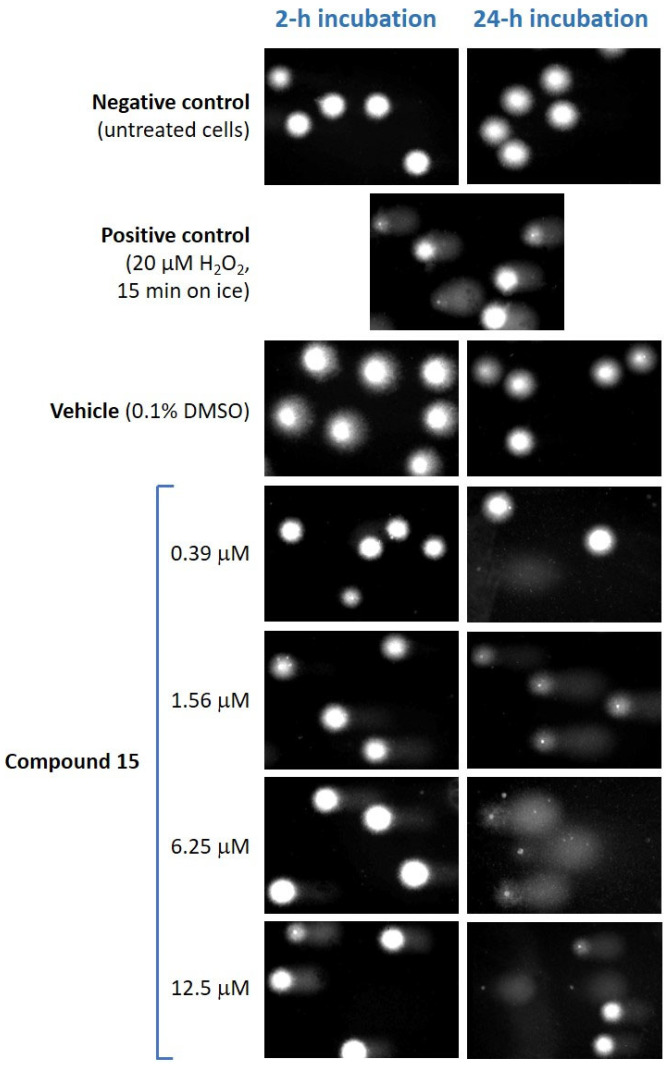
Representative images of the comet effect observed during the comet assay after incubation of PBMCs with compound **15** at concentrations of 0.39, 1.56, 6.25, and 12.5 µM for 2 or 24 h at 37 °C. Negative controls consisted of cells incubated in culture medium only for 2 h at 37 °C. The positive controls were cells incubated with 20 µM H_2_O_2_ for 15 min on ice.

The genotoxic potential of compound **15** was assessed using human PBMCs through the comet assay (Figure 15). This technique detects DNA damage at the single-cell level following exposure to genotoxic agents. It acts as a key indicator of pathogenicity, demonstrating antiproliferative and, consequently, anticancer effects. Generally, the extent of DNA damage in PBMCs treated with compound **15** correlates with its concentration and incubation duration. Four concentrations of compound **15** were chosen to evaluate DNA damage: 0.39, 1.56, 6.25, and 12.5 μM. The compound induces statistically significant DNA damage in PBMCs compared to untreated cells at all tested concentrations (One-way ANOVA and Dunnett’s multiple comparisons test, *p* < 0.01 or *p* < 0.001), even after just 2 h of incubation (Figure 15a). At the highest concentration of 12.5 μM, compound **15** results in DNA damage levels of 36.52 ± 3.96% and 45.37 ± 3.79% after 2 and 24 h of incubation, respectively. The DNA damage caused by compound **15** at 12.5 μM over 2 h and at 6.25 and 12.5 μM over 24 h exceeds that of the positive control, which is 20 μM H_2_O_2_ for 15 min on ice (Figure 15). These findings suggest that compound **15** exhibits genotoxic activity against PBMCs at all tested concentrations. Figure 16 presents example images from the comet assay.

These results, along with the antiproliferative effect observed at higher micromolar concentrations (Figure 10), suggest promising avenues for future research into the therapeutic potential of compound **15** for central nervous system cancers, using cancer cell lines and animal models.

## 3. Materials and Methods

### 3.1. Chemistry

Reagents and solvents were purchased from relevant companies (Chemat Gdańsk, Poland; Sigma Aldrich: Darmstadt, Germany) and used without purification. Purity and identity of synthesised compounds were confirmed by performing thin-layer chromatography (TLC) on Merck silica gel 60F_254_ aluminium sheets (UV light detection and/or stained with Dragendorff’s reagent), and measuring NMR (^1^H and ^13^C) spectra in DMSO-d_6_ on spectrometers (Mercury 300 MHz PFG spectrometer: Varian, Palo Alto, CA, USA; or FT-NMR 500 MHz spectrometer: Jeol Ltd., Akishima, Tokyo, Japan). Mass spectra (LC/MS) were performed on a Waters TQ Detector (Waters Corporation, Milford, CT, USA) mass spectrometer. Retention times (t_R_) are presented in minutes. The UPLC/MS purity of all final compounds was determined (%). All compounds (except **3** and **12**) had purity above 97%.

#### 3.1.1. Synthesis of Starting Compounds

##### Synthesis of Benzyloxyaldehydes—General Method

To a 3- or 4-hydroxybenzaldehyde (12.5 mmol; 1.52 g) in 30 mL of ethanol, potassium carbonate (6.25 mmol; 0.86 g) was added, followed by a proper benzyl chloride (12.5 mmol). The mixture was refluxed for 18 h. The precipitate was then filtered, washed with ethanol, and evaporated. The residue was purified by extraction (DCM: 1% NaOH; DCM: NaCl saturated) or Flash Chromatography (FC) (solvent: DCM).

*3-(benzyloxy)benzaldehyde (***IVa***).* Synthesis from benzyl chloride (1.58 g). Purified by extraction. Yield: 33% (white oily solid).

*3-((4-chlorobenzyl)oxy)benzaldehyde (***IVb***).* Synthesis from 4-chlorobenzyl chloride (2.01 g). Purified by extraction. Yield: 80%.

*3-((3,4-dichlorobenzyl)oxy)benzaldehyde (***IVc***).* Synthesis from 3,4-dichlorobenzyl chloride (2.44 g). Purified by FC. Yield: 21%.

*4-(benzyloxy)benzaldehyde (***Va***).* Synthesis from benzyl chloride (1.58 g). Purified by FC. Yield: 33%.

*4-((4-chlorobenzyl)oxy)benzaldehyde (***Vb***).* Synthesis from 4-chlorobenzylchloride (2.01 g). Purified by extraction. Yield: 89%.

*4-((3,4-dichlorobenzyl)oxy)benzaldehyde (***Vc***).* Synthesis from 3,4-dichlorobenzyl chloride (2.44 g). Purified by extraction. Yield: 80%.

##### Synthesis of 1-(4-(3-Bromo propoxy)phenyl)ethan-1-one (**VI**)

Synthesised from 4-hydroxyacetophenone (0.1 mol; 13.62 g) and 1,3-dibromopropane (0.11 mol; 11.2 mL) in acetone as described by Honkisz-Orzechowska et al. [27].

##### Synthesis of 1-(4-(3-Piperidin-1-yl)propoxy)phenyl)ethan-1-one (**VII**) (CAS256952-65-5)

Synthesised from 1-(4-(3-bromo propoxy)phenyl)ethan-1-one (**VI**) (0.6 mol; 11.49 g), and piperidine (0.6 mol; 4.40 mL) in acetonitrile as described by Honkisz-Orzechowska et al. [27].

#### 3.1.2. General Method of Synthesis of Chalcones

Synthesised from 4-hydroxyacetophenone (1 equivalent) and an appropriate aldehyde (1 equivalent) in absolute ethanol with 15% potassium hydroxide solution as described by Honkisz-Orzechowska et al. [27] or with 40% potassium hydroxide solution. Products were purified by crystallisation from ethanol or by FC (eluent: DCM: methanol; 9:1) and then converted into hydrogen chloride.

*(E)-1-(4-hydroxyphenyl)-3-phenylprop-2-en-1-one (***1***)* CAS2657-25-2. Synthesis from benzaldehyde (5 mmol, 0.53 g) with 40% KOH (4 mL). Purified by crystallisation from ethanol. Yield 34% (380 mg). C_15_H_12_O_2_ (MW 224.25). ^1^H NMR (500 MHz, DMSO-d_6_) δ: 10.40 (br. s., 1H), 8.04 (d, *J* = 8.59 Hz, 2H), 7.88 (d, *J* = 15.47 Hz, 1H), 7.83 (dd, *J* = 1.86, 7.31 Hz, 2H), 7.64 (d, *J* = 15.76 Hz, 1H), 7.36–7.46 (m, 3H), 6.86 (d, *J* = 8.59 Hz, 2H). **UPLC-MS** (*m*/*z*): 225.21 ([M + H]^+^). Purity: 98.85%, t_R_ = 6.93 min.

*(E)-3-(4-chlorophenyl)-1-(4-hydroxyphenyl)prop-2-en-1-one (***2***)* CAS19152-38-6. Synthesis from 4-chlorobenzaldehyde (5 mmol, 0.70 g) with 40% KOH (4 mL). Purified by crystallisation from ethanol. Yield 10% (130 mg). C_15_H_11_ClO_2_ (MW 258.70). **^1^H NMR** (500 MHz, DMSO-d_6_) δ: 10.42 (br. s., 1H), 8.04 (d, *J* = 8.88 Hz, 2H), 7.84–7.94 (m, 3H), 7.63 (d, *J* = 15.76 Hz, 1H), 7.48 (d, *J* = 8.31 Hz, 2H), 6.86 (d, *J* = 8.88 Hz, 2H). **UPLC-MS** (*m*/*z*): 258.70 ([M]^+^). Purity: 99.72%, t_R_ = 7.61 min.

*(E)-3-(3,4-dichlorophenyl)-1-(4-hydroxyphenyl)prop-2-en-1-one (***3***)* CAS 204974-31-2. Synthesis from 3,4-dichlorobenzaldehyde (2.5 mmol, 0.44 g) with 40% KOH (2 mL). Purified by crystallisation from ethanol. Yield 6% (45 mg). C_15_H_10_Cl_2_O_2_ (MW 293.14). **^1^H NMR** (500 MHz, DMSO-d_6_) δ: 10.44 (s, 1H), 8.24 (d, *J* = 1.72 Hz, 1H), 8.07 (d, *J* = 8.60 Hz, 2H), 7.99 (d, *J* = 15.47 Hz, 1H), 7.82 (dd, *J* = 2.01, 8.31 Hz, 1H), 7.65–7.70 (m, 1H), 7.60 (d, *J* = 15.76 Hz, 1H), 6.86 (d, *J* = 8.59 Hz, 2H). **UPLC-MS** (*m*/*z*): 293.03 ([M + H]^+^). Purity: 93.88%, *t_R_* = 8.22 min.

*(E)-3-(3-(benzyloxy)phenyl)-1-(4-hydroxyphenyl)prop-2-en-1-one (***4***).* Synthesis from 3-(benzyloxy)benzaldehyde (5 mmol, 1.06 g) with 40% KOH (4 mL). Purified by crystallisation from ethanol. Yield 24% (390 mg). C_22_H_18_O_3_ (MW 330.38). **^1^H NMR** (500 MHz, DMSO-d_6_) δ: 10.40 (br. s., 1H), 8.05 (d, *J* = 8.88 Hz, 2H), 7.89 (d, *J* = 15.47 Hz, 1H), 7.61 (d, *J* = 15.76 Hz, 1H), 7.55 (s, 1H), 7.46 (d, *J* = 7.45 Hz, 2H), 7.36–7.41 (m, 3H), 7.32 (d, *J* = 7.45 Hz, 2H), 7.05 (dd, *J* = 1.72, 8.02 Hz, 1H), 6.86 (d, *J* = 8.59 Hz, 2H), 5.14 (s, 2H). **UPLC-MS** (*m*/*z*): 331.18 ([M + H]^+^). Purity: 98.22%; *t_R_* = 8.36 min.

*(E)-3-(3-((4-chlorobenzyl)oxy)phenyl)-1-(4-hydroxyphenyl)prop-2-en-1-one (***5***).* Synthesis from 3-((4-chlorobenzyl)oxy)benzaldehyde (5 mmol, 1.23 g) with 40% KOH (4 mL). Purified by crystallisation from ethanol. Yield 18% (330 mg). C_22_H_17_ClO_3_ (MW 364.83). **^1^H NMR** (500 MHz, DMSO-d_6_) δ: 10.41 (br. s., 1H), 8.05 (d, *J* = 8.60 Hz, 2H), 7.89 (d, *J* = 15.76 Hz, 1H), 7.61 (d, *J* = 15.47 Hz, 1H), 7.54 (s, 1H), 7.46–7.51 (m, 2H), 7.41–7.46 (m, 2H), 7.35–7.40 (m, 1H), 7.30–7.35 (m, 1H), 7.04 (dd, *J* = 2.01, 8.02 Hz, 1H), 6.86 (d, *J* = 8.88 Hz, 2H), 5.14 (s, 2H). **UPLC-MS** (*m*/*z*): 365.12 ([M + H]^+^). Purity: 99.11%; *t_R_* = 8.84 min.

*(E)-3-(3-((3,4-dichlorobenzyl)oxy)phenyl)-1-(4-hydroxyphenyl)prop-2-en-1-one (***6***).* Synthesis from 3-((3,4-dichlorobenzyl)oxy)benzaldehyde (6.4 mmol, 1.80 g) with 40% KOH (5 mL). Purified by FC. Yield 13% (340 mg). Yellow solid. C_22_H_16_Cl_2_O_3_ (MW 399.27). **^1^H NMR** (500 MHz, DMSO-d_6_) δ: 10.41 (br. s., 1H), 8.05 (d, *J* = 8.88 Hz, 2H), 7.89 (d, *J* = 15.76 Hz, 1H), 7.74 (d, *J* = 1.72 Hz, 1H), 7.58–7.68 (m, 2H), 7.55 (s, 1H), 7.45 (dd, *J* = 1.72, 8.31 Hz, 1H), 7.37–7.42 (m, 1H), 7.29–7.37 (m, 1H), 7.05 (dd, *J* = 1.86, 7.88 Hz, 1H), 6.86 (d, *J* = 8.60 Hz, 2H), 5.16 (s, 2H). **UPLC-MS** (*m*/*z*) ([M]^+^): 399.09. Purity: 97.87%; *t_R_* = 9.38 min.

*(E)-3-(4-(benzyloxy)phenyl)-1-(4-hydroxyphenyl)prop-2-en-1-one (***7***).* Synthesis from 4-(benzyloxy)benzaldehyde (2.5 mmol, 0.53 g) with 40% KOH (2 mL). Purified by crystallisation from ethanol. Yield 28% (230 mg). C_22_H_18_O_3_ (MW 330.38). **^1^H NMR** (500 MHz, DMSO-d_6_) δ: 10.35 (br s, 1H), 8.02 (d, *J* = 8.59 Hz, 2H), 7.79 (d, *J* = 8.88 Hz, 2H), 7.71–7.77 (m, 1H), 7.58–7.66 (m, 1H), 7.41–7.47 (m, 2H), 7.37 (t, *J* = 7.45 Hz, 2H), 7.27–7.33 (m, 1H), 7.05 (d, *J* = 8.88 Hz, 2H), 6.85 (d, *J* = 8.88 Hz, 2H), 5.14 (s, 2H). **UPLC-MS** (*m*/*z*): 330.92 ([M]^+^). Purity: 100%; *t_R_* = 8.25 min.

*(E)-3-(4-((4-chlorobenzyl)oxy)phenyl)-1-(4-hydroxyphenyl)prop-2-en-1-one (***8***).* Synthesis from 4-((4-chlorobenzyl)oxy)benzaldehyde (2.5 mmol, 0.62 g) with 40% KOH (2 mL). Purified by crystallisation from ethanol. Yield 29% (260 mg). C_22_H_17_ClO_3_ (MW 364.83). **^1^H NMR** (500 MHz, DMSO-d_6_) δ: 10.35 (br. s., 1H), 8.02 (d, *J* = 8.59 Hz, 2H), 7.79 (d, *J* = 8.88 Hz, 2H), 7.71–7.77 (m, 1H), 7.57–7.65 (m, 1H), 7.39–7.49 (m, 4H), 7.04 (d, *J* = 8.59 Hz, 2H), 6.85 (d, *J* = 8.88 Hz, 2H), 5.14 (s, 2H). **UPLC-MS** (*m*/*z*): 364.87 ([M]^+^). Purity: 98.82%; *t_R_* = 8.77 min.

*(E)-3-(4-((3,4-dichlorobenzyl)oxy)phenyl)-1-(4-hydroxyphenyl)prop-2-en-1-one (***9***).* Synthesis from 4-((3,4-dichlorobenzyl)oxy)benzaldehyde (2.5 mmol, 0.71 g) with 40% KOH (2 mL). Purified by FC. Yield 30% (300 mg). C_22_H_16_Cl_2_O_3_ (MW 399.27). **^1^H NMR** (500 MHz, DMSO-d_6_) δ: 10.35 (br. s., 1H), 8.02 (d, *J* = 8.88 Hz, 2H), 7.80 (d, *J* = 8.88 Hz, 2H), 7.73–7.77 (m, 1H), 7.71 (d, *J* = 1.72 Hz, 1H), 7.57–7.67 (m, 2H), 7.43 (dd, *J* = 1.72, 8.31 Hz, 1H), 7.05 (d, *J* = 8.60 Hz, 2H), 6.85 (d, *J* = 8.60 Hz, 2H).

*(E)-3-(phenyl)-1-(4-(3-(piperidin-1-yl)propoxy)phenyl)prop-2-en-1-one hydrogen chloride (***10***).* Synthesis from benzaldehyde (2 mmol, 0.212 g) with 15% KOH (1 mL). Purified by crystallisation (ethanol). Yield 11% (75 mg). C_23_H_27_NO_2_ × HCl (MW 385.93). **^1^H NMR** (500 MHz, DMSO-d_6_) δ: 8.13 (d, *J* = 9.2 Hz, 2H), 7.91 (d, *J* = 15.5 Hz, 1H), 7.84 (dd, *J* = 2.3, 6.9 Hz, 2H), 7.67 (d, *J* = 15.5 Hz, 1H), 7.48–7.36 (m, 3H), 7.05 (d, *J* = 8.6 Hz, 2H), 4.16–4.03 (m, 2H), 3.33 (br. s., 2H), 2.60 (br. s., 4H), 1.97 (br. s., 2H), 1.55 (br. s., 4H), 1.39 (br. s., 2H). **UPLC-MS** (*m*/*z*): 350.30 ([M + H]^+^). Purity: 100%; *t_R_* = 5.11 min.

*(E)-3-(4-chlorophenyl)-1-(4-(3-(piperidin-1-yl)propoxy)phenyl)prop-2-en-1-one hydrogen oxalate (***11***).* Synthesis from 4-chlorobenzaldehyde (2 mmol, 0.281 g) with 15% KOH (1 mL). Purified by crystallisation (ethyl acetate). Yield 9% (78 mg). C_23_H_26_ClNO_2_ × HCl (MW 420.37). **^1^H NMR** (300 MHz, DMSO-d_6_) δ: 8.16 (d, *J* = 8.8 Hz, 1H), 8.02–7.85 (m, 2H), 7.76 (d, *J* = 15.82Hz, 1H), 7.46 (m, 1H), 7.29 (t, *J* = 8.8 Hz, 1H), 7.18–6.91 (m, 3H), 6.78 (d, *J* = 12.9 Hz, 1H), 4.30–3.99 (m, 2H), 3.27–2.78 (m, 6H), 2.23-2.00 (m, 2H), 1.70 (br s, 4H), 1.50 (br. s., 2H). **^13^C NMR** (126 MHz, DMSO-d_6_) δ: 187.8, 165.3, 162.9, 142.3, 135.5, 134.3, 131.6, 131.1, 131.0, 129.5, 123.2, 115.0, 66.0, 53.8, 52.6, 23.9, 23.1, 22.0. **UPLC-MS** (*m*/*z*): 384.06 ([M + H]^+^). Purity: 99.51%; *t_R_* = 5.65 min.

*(E)-3-(3,4-dichlorophenyl)-1-(4-(3-(piperidin-1-yl)propoxy)phenyl)prop-2-en-1-one hydrogen chloride (***12***).* Synthesis from 3,4-dichlorobenzaldehyde (2 mmol, 0.35 g) with 15% KOH (1 mL). Purified by crystallisation from ethanol. Yield 26% (0.235 g). C_23_H_25_Cl_2_O_2_ × HCl (MW 454.82). **^1^H NMR** (300 MHz, DMSO-d_6_) δ: 10.35 (br. s., 1H), 8.27 (d, *J* = 1.76 Hz, 1H), 8.19 (d, *J* = 8.79 Hz, 2H), 8.05 (d, *J* = 15.82 Hz, 1H), 7.86 (dd, *J* = 1.76, 8.79 Hz, 1H), 7.70 (d, *J* = 8.21 Hz, 1H), 7.65 (d, *J* = 15.24 Hz, 1H), 7.08 (d, *J* = 8.79 Hz, 2H), 4.18 (t, *J* = 6.15 Hz, 2H), 3.38–3.54 (m, 2H), 3.15 (d, *J* = 4.69 Hz, 2H), 2.88 (d, *J* = 7.62 Hz, 2H), 2.12–2.32 (m, 2H), 1.61–1.89 (m, 5H), 1.39 (br. s., 1H). **^13^C NMR** (126 MHz, DMSO-d_6_) δ: 187.6, 163.0, 141.0, 136.2, 133.1, 132.3, 131.7, 131.5, 130.9, 130.6, 129.7, 124.5, 115.0, 66.1, 53.8, 52.5, 23.7, 22.8, 21.9. **UPLC-MS** (*m*/*z*): 418.22 ([M]^+^). Purity: 94.80%; *t_R_* = 6.37 min.

*(E)-3-(3-(benzyloxy)phenyl)-1-(4-(3-(piperidin-1-yl)propoxy)phenyl)prop-2-en-1-one hydrogen chloride (***13***).* Synthesis from 3-(benzyloxy)benzaldehyde (1 mmol, 0.22 g) with 15% KOH (0.5 mL). Purified by FC. Yield 8% (40 mg). C_30_H_33_NO_3_ × HCl (MW 492.05). **^1^H NMR** (500 MHz, DMSO-d_6_) δ: 10.40 (br. s., 1 H), 8.16 (d, *J* = 8.6 Hz, 2H), 7.93 (d, *J* = 15.8 Hz, 1H), 7.64 (d, *J* = 15.5 Hz, 1H), 7.56 (br. s., 1H), 7.46 (d, *J* = 7.2 Hz, 2H), 7.42–7.23 (m, 5H), 7.06 (d, *J* = 8.6 Hz, 3H), 5.15 (s, 2H), 4.15 (t, *J* = 5.7 Hz, 2H), 3.41 (d, *J* = 11.2 Hz, 2H), 3.14 (d, *J* = 4.9 Hz, 2H), 2.93–2.72 (m, 2H), 2.29–2.07 (m, 2H), 1.75 (br. s., 4H), 1.67 (d, *J* = 12.9 Hz, 1H), 1.34 (d, *J* = 7.2 Hz, 1H). **^13^C NMR** (126 MHz, DMSO-d_6_) δ: 187.9, 162.8, 159.3, 143.7, 137.5, 136.8, 131.5, 131.1, 130.5, 129.0, 128.5, 128.4, 122.8, 122.5, 117.7, 115.0, 114.7, 69.9, 66.0, 53.8, 52.5, 23.7, 22.9, 21.9. **UPLC-MS** (*m*/*z*): 456.41 ([M + H]^+^). Purity: 100%; *t_R_* = 6.46 min.

*(E)-3-(3-((4-chlorobenzyl)oxy)phenyl)-1-(4-(3-(piperidin-1-yl)propoxy)phenyl)prop-2-en-1-one hydrogen chloride (***14***).* Synthesis from 3-((4-chlorobenzyl)oxy)benzaldehyde (0.5 mmol, 0.13 g) with 15% KOH (0.25 mL). Purified by FC. Yield 23% (60 mg). C_30_H_32_NO_3_Cl × HCl (MW 526.48). **^1^H NMR** (500 MHz, DMSO-d_6_) δ: 10.35 (br. s., 1H), 8.15 (d, *J* = 8.59 Hz, 2H), 7.93 (d, *J* = 15.47 Hz, 1H), 7.64 (d, *J* = 15.75 Hz, 1H), 7.56 (s, 1H), 7.42–7.52 (m, 4H), 7.40 (d, *J* = 7.73 Hz, 1H), 7.31–7.37 (m, 1H), 7.06 (d, *J* = 8.59 Hz, 3H), 5.15 (s, 2H), 4.15 (t, *J* = 5.87 Hz, 2H), 3.41 (d, *J* = 11.74 Hz, 2H), 3.09–3.19 (m, 2H), 2.76–2.91 (m, 2H), 2.12–2.28 (m, 2H), 1.75 (br. s., 4H), 1.66 (d, *J* = 13.17 Hz, 1H), 1.35 (dd, *J* = 7.45, 13.17 Hz, 1H). **^13^C NMR** (126 MHz, DMSO-d_6_) δ: 187.8, 162.8, 159.1, 143.6, 136.8, 136.5, 133.0, 131.6, 131.1, 130.5, 130.3, 129.0, 122.8, 122.6, 117.7, 115.0, 114.8, 69.1, 66.0, 53.8, 52.6, 23.7, 22.9, 21.9. **UPLC-MS** (*m*/*z*): 490.37 ([M]^+^). Purity: 100%; *t_R_* = 6.87 min.

*(E)-3-(3-((3,4-dichlorobenzyl)oxy)phenyl)-1-(4-(3-(piperidin-1-yl)propoxy)phenyl)prop-2-en-1-one hydrogen chloride (***15***).* Synthesis from 3-((3,4-dichlorobenzyl)oxy)benzaldehyde (1 mmol, 0.28 g) with 15% KOH (0.5 mL). Purified by FC. Yield 23% (130 mg). C_30_H_31_NO_3_Cl_2_ × HCl (MW 560.94). **^1^H NMR** (500 MHz, DMSO-d_6_) δ: 10.55 (br. s., 1H), 8.16 (d, *J* = 8.59 Hz, 2H), 7.94 (d, *J* = 15.75 Hz, 1H), 7.74 (d, *J* = 1.72 Hz, 1H), 7.65 (d, *J* = 2.29 Hz, 1H), 7.61–7.64 (m, 1H), 7.57 (s, 1H), 7.45 (dd, *J* = 2.00, 8.31 Hz, 1H), 7.39–7.43 (m, 1H), 7.34 (t, *J* = 7.88 Hz, 1H), 7.02–7.11 (m, 3H), 5.17 (s, 2H), 4.15 (t, *J* = 6.01 Hz, 2H), 3.41 (d, *J* = 11.74 Hz, 2H), 3.06–3.20 (m, 2H), 2.75–2.93 (m, 2H), 2.13–2.28 (m, 2H), 1.70–1.92 (m, 4H), 1.66 (d, *J* = 12.89 Hz, 1H), 1.28–1.42 (m, 1H). **^13^C NMR** (126 MHz, DMSO-d_6_) δ: 187.8, 162.8, 158.9, 143.6, 138.7, 136.8, 131.7, 131.6, 131.3, 131.1, 131.0, 130.6, 130.2, 128.6, 122.9, 122.8, 117.6, 115.0, 114.8, 68.3, 65.9, 53.8, 52.6, 23.8, 23.0, 21.9. **HR-MS (ESI-QTOF)** calcd for C_30_H_31_NO_3_Cl_2_ [M + H]^+^: 524.1761; found: 524.1724. **UPLC-MS** (*m*/*z*): Purity: 95.82%; *t_R_* = 7.98 min.

*(E)-3-(4-(benzyloxy)phenyl)-1-(4-(3-(piperidin-1-yl)propoxy)phenyl)prop-2-en-1-one hydrogen chloride (***16***).* Synthesis from 4-(benzyloxy)benzaldehyde (1 mmol, 0.21 g) with 15% KOH (0.5 mL). Purified by crystallisation from ethanol. Yield 22% (110 mg). C_30_H_33_NO_3_ x HCl (MW 492.05). **^1^H NMR** (500 MHz, DMSO-d_6_) δ: 10.65 (br. s., 1H), 8.13 (d, *J* = 8.59 Hz, 2H), 7.75–7.84 (m, 3H), 7.61–7.68 (m, 1H), 7.40–7.46 (m, 2H), 7.37 (t, *J* = 7.45 Hz, 2H), 7.27–7.33 (m, 1H), 7.01–7.11 (m, 4H), 5.15 (s, 2H), 4.15 (t, *J* = 6.01 Hz, 2H), 3.40 (d, *J* = 10.88 Hz, 2H), 3.12 (br. s., 2H), 2.83 (d, *J* = 10.31 Hz, 2H), 2.10–2.25 (m, 2H), 1.71–1.89 (m, 4H), 1.66 (d, *J* = 12.89 Hz, 1H), 1.27–1.43 (m, 1H). **^13^C NMR** (126 MHz, DMSO-d_6_) δ: 187.7, 162.6, 160.8, 143.7, 137.3, 131.4, 131.2, 129.0, 128.5, 128.3, 128.2, 120.1, 115.7, 115.0, 69.9, 66.0, 53.8, 52.5, 23.7, 22.8, 22.0. **UPLC-MS** (*m*/*z*): 456.40 ([M + H]^+^). Purity: 100%; *t_R_* = 6.32 min.

*(E)-3-(4-((4-chlorobenzyl)oxy)phenyl)-1-(4-(3-(piperidin-1-yl)propoxy)phenyl)prop-2-en-1-one hydrogen chloride (***17***).* Synthesis from 4-((4-chlorobenzyl)oxy)benzaldehyde (2 mmol, 0.49 g) with 15% KOH (1 mL). Purified by FC. Yield 32% (340 mg). C_30_H_32_NO_3_Cl x HCl (MW 526.49). **^1^H NMR** (500 MHz, DMSO-d_6_) δ: 10.81 (br. s., 1H), 8.16 (d, *J* = 8.59 Hz, 2H), 7.94 (d, *J* = 15.47 Hz, 1H), 7.64 (d, *J* = 15.47 Hz, 1H), 7.57 (br. s., 1H), 7.46–7.52 (m, 2H), 7.37–7.45 (m, 3H), 7.33 (t, *J* = 7.88 Hz, 1H), 7.06 (d, *J* = 8.59 Hz, 3H), 5.15 (s, 2H), 4.15 (t, *J* = 5.87 Hz, 2H), 3.38–3.47 (m, 2H), 3.12 (br. s., 2H), 2.83 (d, *J* = 10.31 Hz, 2H), 2.14–2.28 (m, 2H), 1.59–1.91 (m, 5H), 1.25–1.43 (m, 1H). **^13^C NMR** (126 MHz, DMSO-d_6_) δ: 187.8, 162.8, 159.1, 143.6, 136.8, 136.5, 133.0, 131.6, 131.1, 130.5, 130.2, 129.0, 122.8, 115.0, 114.7, 69.1, 66.1, 53.8, 52.5, 23.6, 22.8, 22.0. **LC-MS** (*m*/*z*): 490.35 [M + H]+; C_30_H_32_NO_3_Cl (calculated MW: 490.21). Purity (UPLC-MS): 100%; *t*_R_ = 6.81. **UPLC-MS** (*m*/*z*): 490.04 ([M]^+^). Purity: 97.08%; *t_R_* = 6.81 min.

*(E)-3-(4-((3,4-dichlorobenzyl)oxy)phenyl)-1-(4-(3-(piperidin-1-yl)propoxy)phenyl)prop-2-en-1-one hydrogen chloride (***18***).* Synthesis from 4-((3,4-dichlorobenzyl)oxy)benzaldehyde (1 mmol, 0.28 g) with 15% KOH (0.5 mL). Purified by FC. Yield 32% (340 mg). C_30_H_31_NO_3_Cl_2_ x HCl (MW 560.94). **^1^H NMR** (500 MHz, DMSO-d_6_) δ: 10.54 (br. s., 1H), 8.12 (d, *J* = 8.59 Hz, 2H), 7.76–7.87 (m, 3H), 7.71 (d, *J* = 0.86 Hz, 1H), 7.59–7.67 (m, 2H), 7.42 (d, *J* = 7.16 Hz, 1H), 7.05 (dd, *J* = 6.59, 8.02 Hz, 4H), 5.16 (s, 2H), 4.14 (t, *J* = 5.73 Hz, 2H), 3.40 (d, *J* = 10.31 Hz, 2H), 3.13 (br. s., 2H), 2.83 (br. s., 2H), 2.20 (dd, *J* = 5.87, 9.02 Hz, 2H), 1.59–1.86 (m, 5H), 1.27–1.41 (m, 1H). **^13^C NMR** (126 MHz, DMSO-d_6_) δ: 187.7, 162.6, 160.4, 143.6, 138.5, 131.7, 131.4, 131.3, 131.3, 131.3, 131.0, 130.1, 128.5, 120.3, 115.8, 115.0, 68.3, 66.0, 53.8, 52.5, 23.7, 22.9, 21.9. **UPLC-MS** (*m*/*z*): 524.24 ([M]^+^). Purity: 97.99%; *t_R_* = 7.97 min.

### 3.2. In Vitro Biological Studies

#### 3.2.1. Radioligand Binding Assay to Human Histamine H_3_ Receptor

The affinity for the hH_3_R was assessed using a radioligand binding assay in CHO K1 cells, employing N^α^-methylhistamine as the radioligand, following the procedure described by Łażewska et al. [20]. 10 mM stock solutions of the tested compounds were prepared in DMSO. Serial dilutions of compounds were prepared in a 96-well microplate using assay buffers with an automated pipetting system, epMotion 5070 (Eppendorf, Hamburg, Germany). Each compound was tested in 8 concentrations from 10^−5^ to 10^−12^ M (final concentration). All assays were performed in duplicate. 50 µL working solution of the tested compounds, 50 µL [^3^H]-N^α^-methylhistamine (spec. act. 82.9 Ci/mmol, final concentration 1.0 nM) and 150 µL diluted membranes (15 µg protein per well) prepared in assay buffer (50 mM Tris, pH 7.4, 5 mM MgCl_2_) were transferred to polypropylene 96-well microplate using 96-well pipetting station, Rainin Liquidator (Mettler Toledo Inc., Greifensee, Switzerland ). (R)(-)-α-methylhistamine (100 μM) was used to define nonspecific binding. The microplate was covered with sealing tape, mixed, and incubated for 60 min at 27 °C. The reaction was terminated by rapid filtration through a GF/B filter mate presoaked with 0.5% polyethyleneimine for 30 min. Five rapid washes with 300 µL 50 mM Tris buffer (4 °C, pH 7.4) were performed using a 96-well FilterMate harvester (PerkinElmer, Shelton, CT, USA). The filter mates were dried at 37 °C in a forced air fan incubator, and then the solid scintillator MeltiLex was melted on the filter mates at 90 °C for 4 min. Radioactivity was counted in a MicroBeta2 scintillation counter (PerkinElmer, Shelton, CT, USA). Data were fitted to a one-site curve-fitting equation with Prism 5 (GraphPad Software 8.0), and K_i_ values were estimated from the Cheng−Prusoff equation [43].

#### 3.2.2. Functional Assays for Histamine H_3_ Receptor

Stock solutions (10 mM) of the reference and tested compounds (**12** and **15**) were prepared in DMSO. Serial dilutions were performed in 96-well assay plates using the assay buffer (5 mM HEPES, 0.5 mM IBMX, pH 7.4), resulting in 8 concentrations for each compound. The adenylyl cyclase activity mediated by the hH_3_R was measured in cryopreserved CHO-K1 cells stably transfected with the receptor (Revvity, Waltham, MA, USA). After thawing, cells were resuspended in stimulation to a final density of 1.7 × 10^5^ cells/mL. For each assay condition, 6 µL of the cell suspension was combined with 3 µL of the compound dilution in white, opaque 384-well plates. In antagonist mode, α-methylhistamine (100 nM) was used as the reference agonist and was added simultaneously with the test compound. The plates were incubated for 1 h at room temperature. After incubation, intracellular cAMP levels were determined using the homogeneous TR-FRET LANCE Ultra cAMP assay kit (Revvity, Waltham, MA, USA). Six microliters each of Eu-cAMP tracer and ULight-anti-cAMP solutions were added per well, mixed, and incubated for an additional 1 h. TR-FRET signals were measured using an EnVision multimode plate reader (PerkinElmer, Shelton, CT, USA). Concentration–response data were fitted by nonlinear regression using GraphPad Prism 8.0 software.

#### 3.2.3. Surface Plasmon Resonance Microscopy (SPRM) Kinetic Studies

*Cell culture conditions.* Cells were cultured according to the manufacturer’s protocol provided by Revvity (Waltham, MA, USA). CHO-K1 cells overexpressing the hH_3_R were cultured in Ham’s F12 Nutrient Mix (ThermoFisher, Waltham, MA, USA) containing 10% foetal bovine serum (ThermoFisher, Waltham, MA, USA), 500 units/mL penicillin, and 500 μg/mL streptomycin (Sigma Aldrich, Darmstadt, Germany) at 37 °C in a humidified incubator (5% CO_2_). Geneticin (400 µg/mL) was used as a selective antibiotic for receptor expression selection.

*Preparation of solutions of test and reference compounds.* 10 mM stock solutions of the tested compounds were prepared in DMSO. Serial dilutions were prepared in glass tubes using buffer (Phosphate-Buffered Saline with 0.01% BSA). The final concentrations of the test and reference compounds ranged from 10 µM to 0.5 nM, specifically: 10 µM, 5 µM, 1 µM, 500 nM, 100 nM, 50 nM, 10 nM, 5 nM, 1 nM and 0.5 nM.

*SPRM Analysis.* CHO-K1 cell line transfected with hH_3_R was seeded at a density of 20,000 cells on a sterile gold SPRm chip, which was pre-coated with 100 µg/mL Poly-D-Lysine as an adhesion factor. After 48 h of incubation, the cells were washed with DPBS and subsequently fixed with 4% paraformaldehyde (PFA) for 10 min. After PFA removal and subsequent washing with DPBS, cells were incubated with a 3% BSA solution for 1h to reduce nonspecific binding. Following the application of a running buffer (PBS with 0.01% BSA), the chip was mounted onto the SPRm device (Biosensing Instruments Inc., Tempe, AZ, USA). The tested compounds were then applied at six to seven increasing concentrations for analysis. Data were analysed using the Biosensing Instruments SPRm Data Analysis Program. The association rate constant (k_a_), dissociation rate constant (k_d_), and equilibrium dissociation constant (K_D_) were obtained from affinity analyses of ROI sensograms.

#### 3.2.4. Human MAO-B and MAO-A Inhibitory Activity

The compounds were evaluated by the spectrophotometric method described earlier [20]. The enzymes were purchased from Sigma-Aldrich (Steinheim, Germany). Screening was carried out at a concentration of 1 μM at the MAO-B and 10 μM at the MAO-A. The assay was carried out in a 96-well plate. 2 µL of an appropriate concentration of tested compounds in DMSO were added to wells that contained 98 µL of enzyme dilution (0.52 U/well) in phosphate buffer (50 mM, pH 7.4). After the 30 min of preincubation at room temperature, 50 µL of the solution of 800 μM 10-acetyl-3,7-dihydroxyphenoxazine (Cayman Chemical, Ann Arbor, MI, USA) and 4 U/mL horse radish peroxidase (HRP) was added, and the enzymatic reaction was started by the addition of 50 µL of 800 µM *p*-tyramine solution. The signal was measured after 1 h (excitation at 570 nm and emission at 585 nm) using Spark^®^ multimode microplate reader (Tecan; Männedorf, Switzerland). The compounds that showed more than 50% inhibition were subjected to further testing to determine the IC_50_ value. Data are expressed as mean values ± SEM of at least two independent experiments.

#### 3.2.5. Human MAO-B Reversibility Studies

Enzyme was incubated with inhibitors (compound **15**, safinamide and rasagiline) in a concentration equivalent to 10 × IC_50_ for 30 min. The mixture was diluted 100× in the buffer containing MAO-B substrate (p-tyramine) and detection reagent (horseradish peroxidase and 10-Acetyl-3,7-dihydroxyphenoxazine). Fluorescence signal was measured every 5 min for 1 h. Baseline (fluorescence signal of reaction mixture without p-tyramine) was subtracted from all samples.

#### 3.2.6. Human MAO-B Kinetic Studies

To determine the inhibitory mode of human MAO-B inhibition, substrate-dependent kinetic experiments were performed. The data allowed the generation of progress curves and Lineweaver–Burk plots. Measurements of the enzyme’s initial catalytic rates were conducted using six different p-tyramine concentrations (0.05–2.0 mM), both without inhibitor and with compound **15** added at three concentrations corresponding to its IC_20_, IC_50_, and IC_80_ values. These experiments followed the assay conditions for hMAO-B described earlier.

### 3.3. Molecular Modelling Studies of the Histamine H_3_ Receptor and MAO-B

For docking purposes, Schrodinger 2022-4 [44] was used. Ligand structures in their E isomeric forms were built using Maestro 2D sketcher, and their 3D conformations were obtained using the LigPrep module (OPLS4, target pH = 7.4 ± 0.5). Protein structures of the histamine H_3_ receptor in its inactive form, and MAO-B complexed with an inhibitor, were obtained from the PDB database (ID codes: 7F61 and 2V5Z, respectively). In the original 7F61 structure publication [36], a cholesterol molecule was resolved at an allosteric site, where it was suggested to contribute to receptor inactive-state stabilisation; accordingly, the cholesterol molecule was retained during modelling to account for its potential modulatory role in receptor conformation and ligand binding. An initial relaxation protocol into a local energy minimum using Brownian motion simulation (in time of 100 ps) was performed with Desmond [45]. To validate the methods used, the native ligands were redocked with high confidence (glide RMSD to input of 0.181 and 0.207, respectively). Tested ligands were then docked using Glide XP (7F61) and Induced Fit (2V5Z, since no poses for compound **15** were returned in standard XP protocol) protocols with grid box centred on co-resolved ligand [46,47], followed by ligand energy calculation using Prime MM–GBSA [48]. Binding pose metadynamics on the top 5 scoring complexes was then performed (10 trials per pose) so as to predict the correct pose. Dynamics simulations (time 250 ns, T = 300 K) were run in Desmond [44]. For each run, 1000 frames were produced. The obtained trajectories were then analysed visually, as well as using the Simulation Interaction Analysis tool of Desmond/Maestro. Membrane orientations of the 7F61 protein complexes were computed with the PPM 3.0 server [49], applying the TIP3P solvent model [50] and embedding them in a POPC membrane. For the 2V5Z system, solvation was performed using the TIP3P model within an orthorhombic box extending 10 Å beyond the solute in each dimension. Per-residue RMSD and MM–GBSA ΔG Bind values were calculated using Schrödinger Maestro command line scripts. All of the figure’s components come from the Schrödinger package and were prepared using freely available graphics software.

### 3.4. Preliminary ADMET Evaluation of Compounds ***12*** and ***15***

#### 3.4.1. Permeability Evaluation

The passive permeability assessment was performed by the Pre-coated PAMPA Plate System Gentest^TM^ Corning (Tewksbury, MA, USA) according to the procedure provided by the manufacturer. In brief, the tested compounds **12** and **15** and the reference permeable caffeine were diluted first in the PBS buffer (pH 7.4). In order to increase the unsatisfying solubility of compounds **12** and **15**, 20% of methanol was added to PBS. The compounds were applied next to the PAMPA donor plate at a final concentration of 100 μM. After 5 h of incubation at RT, UPLC-MS spectrometry with internal standard was used to estimate the quantity of compounds that penetrated from donor to acceptor wells. The permeability coefficients (P_e_, cm/s) were calculated using the formulas provided by Corning. The Caco-2 cell line HTB-37^TM^ (ATCC, Manassas, VA, USA) was cultivated in Dulbecco’s Modified Eagle’s Medium (DMEM) supplemented with 10% foetal bovine serum (FBS) (both obtained from ThermoFisher Scientific, Waltham, MA, USA) until the appropriate confluence (70–80%) was achieved. Next, the cells were seeded into Corning^®^ 3413 Transwell^®^ 6.5 mm polycarbonate membrane inserts with 0.4 µm pores purchased from Sigma-Aldrich (Saint Louis, MO, USA). Cells were seeded at a 2 × 10^−4^ concentration per insert in the apical compartment, and 600 µL of media was added to the basolateral one. TEER (transepithelial electrical resistance) measurements were performed by Millicell ERS-2 Volt-Ohm Metre (Merck Millipore, Burlington, MA, USA) in order to confirm the membrane integrity, ready for the experiment. Around 20–21 days post-seeding, the monolayer was rinsed with HBSS (Hank’s balanced salt solution), and compounds **12** and **15**, as well as reference (caffeine), were tested at 10 µM concentration in HBSS. Compounds were added together with the integrity marker lucifer yellow either into the apical chambers (A-B direction) or basolateral chambers (B-A direction). The plate was placed in the orbital shaker (60 rpm) for 2 h at 37 °C. The samples were collected, and the compounds’ concentrations were analysed using the UPLC-MS method with an internal standard. The fluorescence of lucifer yellow was measured by using a Synergy H1 microplate reader (BioTek, Winooski, VT, USA) to confirm the membrane integrity. The apparent permeability P_app_ was calculated according to the following formula: P_app_ = dc/dt × V/(A × C_0_).

#### 3.4.2. Metabolic Stability Studies

The most probable metabolic pathways of compounds **12** and **15** were determined by incubation with rat liver microsomes (RLMs, Sigma-Aldrich, St. Louis, MO, USA) in potassium phosphate buffer (100 mM, pH 7.4, 37 °C). The reactions were terminated at 2 h by the addition of cold methanol and centrifuged. The UPLC-MS analyses were performed on the supernatants. The determination of metabolites from the obtained MS spectra was supported by MetaSite 6.0.1 software (Molecular Discovery Ltd., Hertfordshire, UK). The PK in vitro parameters of 15 were estimated in mouse liver microsomes (MLMs) obtained from Sigma-Aldrich. The reaction mixtures consisted of potassium phosphate buffer (100 mM, pH 7.4), mouse liver microsomes (0.5 mg/mL), tested compound (1 µM) and NADPH (1 mM). The five independent reactions were terminated at 0, 5, 15, 30 and 45 min by the addition of cold methanol containing IS and centrifuged. The UPLC-MS analyses were performed next on the supernatants. Verapamil was used as the reference unstable drug. The t_1/2_ value and intrinsic clearance CL_int_ were calculated using the protocols and formulas proposed by Obach [51]. The microsomal protein/g of liver weight was considered as 45 mg, and the liver weight was 88 g/kg of body weight in the mouse.

#### 3.4.3. Drug–Drug Interaction Studies

The prediction of drug–drug interactions was performed using the CYP3A4 and CYP2D6 P450-Glo^TM^ assays purchased from Promega (Madison, WI, USA). The influence of compounds on CYP’s activity was tested in white polystyrene, flat-bottom Nunc^TM^ MicroWell^TM^ 96-well microplates (Thermo Scientific, Waltham, MA, USA). The bioluminescence signal was measured with a microplate reader, Synergy H1 (BioTek, Winooski, VT, USA) in luminescence mode. Compounds **12** and **15** were tested in triplicate in two independent experiments in the range of 0.1–25 µM. The 1 µM of selective CYP inhibitor ketoconazole (3A4) and quinidine (2D6) was used as the positive control.

#### 3.4.4. Preliminary Cell Toxicity

Cytotoxicity tests were performed in hepatoma HepG2 (ATCC^®^ HB-8065 ^TM^; ATCC; Manassas, VA, USA), neuroblastoma SH-SY5Y (ATCC^®^ CRL-2266^TM^; ATCC; Manassas, VA, USA) and colorectal adenocarcinoma Caco-2 (ATCC^®^ HTB-37^TM^; ATCC; Manassas, VA, USA) cell lines. The viability of cells was assessed using the colourimetric MTS assay (CellTiter96^®^ Aqueous One Solution Cell Proliferation Assay kit, Promega, Madison, WI, USA). All cell culture media were purchased from ThermoFisher Scientific (Waltham, MA, USA). Cells were cultured under standard conditions (37 °C, 5% CO_2_) in the recommended ATCC media supplemented with 10% (HepG2, SH-SY5Y) or 20% (Caco-2) of FBS. Before the assay, HepG2 cells were seeded into 96-well plates in 1.5 × 10^4^ cells/well, whereas Caco-2 and SH-SY5Y cells were seeded in 0.5 × 10^4^ cells/well. After 24 h of incubation, the medium was replaced, and cells were treated with the tested compounds **12** and **15**. Doxorubicin was also added as a positive control. Following 72 h (HepG2, SH-SY5Y) or 48 h (Caco-2) exposure, the medium was removed, and 100 μL of fresh medium containing MTS reagent was added. After 2 h of incubation, absorbance at 490 nm was recorded using a Synergy H1 microplate reader (BioTek, Winooski, VT, USA). Wells without cells served as blanks, while 1% DMSO-treated wells represented controls for cell viability.

### 3.5. Activity Profile of Compound ***15***—In Vitro Studies

#### 3.5.1. Cell Culture

The neuroblastoma SH-SY5Y cell line was obtained from the American Type Culture Collection (ATCC; Manassas, VA, USA). The cells were cultured in DMEM/F-12 (Biowest, Nuaillé, France) supplemented with 10% foetal bovine serum (PAN-Biotech, Aidenbach, Germany), non-essential amino acids (Biowest, Nuaillé, France), and antibiotics (Life Technologies, Carlsbad, CA, USA) at 37 °C in a humidified atmosphere containing 5% CO_2_. Peripheral blood mononuclear cells (PBMCs) were isolated from a leucocyte buffy coat collected from the blood of healthy non-smoking donors at the Blood Bank in Lodz, Poland. These cells include lymphocytes, monocytes, and other white blood cells with a round nucleus. The study protocol was approved by the Committee for Research on Human Subjects at the University of Lodz, Poland (12/KEBNUŁ/I/2024-2025) on 17 December 2024.

#### 3.5.2. Cytotoxicity Assessment of Compound **15** and Its Effect on Cell Proliferation

The cytotoxicity of compound **15** was evaluated using PBMCs and SH-SY5Y cells. Additionally, in studies on SH-SY5Y cells, the effect of the compound on the cell cycle (by determining the 2N/4N DNA ratio index), as well as mitochondrial potential and mass, was checked (High Content Analysis Image Cytometry).

##### Effect of Compound **15** on Human PBMCs Viability

A portion of the leucocyte buffy coat was diluted in 1% phosphate-buffered saline (PBS), centrifuged, and cells were prepared as previously described in detail [52,53]. The cell pellet was resuspended in RPMI 1640 medium (Lonza, Basel, Switzerland), and the resazurin reduction assay was performed in the same manner as described previously by O’Brien et al. [54]. Resazurin salt powder was dissolved in sterile PBS. Cells were seeded on the 96-well plates at 5 × 10^4^ for PBMCs per well. Compound **15** was added to wells to achieve ten final concentrations within a range of 0.195–100 µM. Subsequently, the plates were incubated at 37 °C in 5% CO_2_ for 2, 24, 48, and 72 h. Afterwards, 10 µL of resazurin salt was added to each well, and the plates were incubated again under the same conditions for 2 h. Next, fluorescence was measured with a microplate reader Synergy HT (BioTek Instruments, Winooski, VT, USA) using an excitation wavelength of 530/25 nm and an emission wavelength of 590/35 nm. The viability of PBMCs was assessed after 2, 24, 48, and 72 h of incubation with compound **15**. The viability for each sample was calculated relative to the negative control (NC, untreated cells), which was set at 100%. The vehicle (Veh) consisted of PBMCs incubated with 0.1% DMSO. The results, expressed as means with SEM from four independent experiments, were given as percentages of untreated control cells. Values were plotted against all tested concentrations of compound **15** to determine the viability inhibition concentration at 50% (IC_50_) using GraphPad Prism 6.07 (GraphPad Software, Inc., San Diego, CA, USA).

##### Assessment of the Effect of Compound **15** on SH-SY5Y Cell Proliferation, Mitochondrial Membrane Potential, and Mitochondrial Mass (High Content Analysis Image Cytometry)

The effect of compound **15** on cell proliferation and the cellular mitochondrial mass was assessed using image cytometry as described previously [53,55,56]. SH-SY5Y cells were initially seeded in 96-well plates at a density of 5000 cells per well. Twenty-four hours later, the cells were treated with various concentrations of compound **15** (range: 0.39–6.25 μM) or pitolisant (concentration range: 0.39–25 μM) for 24 or 72 h. Then, after adding MitoTracker Orange CMTMRos (500 nM) (Invitrogen, Eugene, Oregon, USA), the cells were incubated for 30 min, washed with PBS, fixed with 4% formaldehyde for 20 min and stained with 1 µg/mL Hoechst 33,342 (Life Technologies, Eugene, OR, USA) for 30 min at room temperature. Images of the cells were taken in two runs using an ArrayScan VTI HCS Reader (ThermoFisher Scientific, Inc., Waltham, MA, USA). First, a series of detailed images was taken with an ArrayScan VTI HCS Reader equipped with a 10x objective, and single cells were analysed with Cell Health Profiling Bioapplication V3 software (200 cells per well).

*The mitochondrial mass* was expressed as average total fluorescence (average of 200 cells). *Cell number* was calculated as the sum of the Hoechst-33342-stained cell nuclei using Cell Cycle Bioapplication V3 software and expressed as a percentage of control. The remaining adherent cells in culture wells indicated the cytotoxic potential of the tested compound. IC_50_ values were calculated with a nonlinear fit to a sigmoidal dose—response curve (log compound vs. normalised response) via GraphPad Prism 6.07. Next, *the DNA content of single SH-SY5Y cells was examined*. A series of detailed images was captured using the ArrayScan VTI HCS Reader equipped with a 10× objective, collecting at least 500 nuclei per replicate. The DNA content of single cells was analysed based on Hoechst-33342 fluorescence intensity of their nuclei using Cell Cycle Bioapplication V3 software, and expressed as a DNA 2N/4N ratio. This index indicates the ratio of cells in the G0/G1 phase (DNA content = 2N, where N represents haploid DNA content) to those in the G2/M phase (DNA content = 4N). All experiments were conducted three times, each with four replicates. IC_50_ values were determined by fitting a nonlinear model to a sigmoidal dose–response curve (log compound versus normalised response) using GraphPad Prism 6.07. The results are presented as mean ± SEM from at least three independent experiments. Statistical significance was evaluated using one-way ANOVA followed by Dunnett’s test. * *p* < 0.05, ** *p* < 0.01, and *** *p* < 0.001 indicate significant differences compared to control counterparts.

#### 3.5.3. The Comet Assay—DNA Damage

The genotoxic potential of compound **15** was evaluated using the comet assay. The alkaline comet assay is a sensitive and straightforward method for detecting DNA damage, including single- and double-strand breaks, as well as alkaline-labile sites, in living cells. PBMCs were used in this study. Compound **15** was added to the PBM cell suspension to achieve final concentrations ranging from 0.39 to 12.5 µM. PBMCs were incubated for 2 h or 24 h at 37 °C in 5% CO_2_. The experiment included, in addition to the Negative Control (NC, untreated cells) and Vehicle (Veh, PBMCs in 0.1% DMSO), a positive control (PC), which was a cell sample incubated with 25 µM H_2_O_2_ for 15 min on ice. The comet assay was performed under alkaline conditions according to the procedure described by Tokarz et al. [57]. A freshly prepared PBMC suspension in 0.75% LMP agarose dissolved in PBS was layered onto microscope slides (Superior, Germany), which were pre-coated with 0.5% NMP agarose. Then, the cells were lysed for 1 h at 4 °C in a buffer containing 2.5 M NaCl, 0.1 M EDTA, 10 mM Tris, 1% Triton X-100, pH = 10. After lysis, electrophoretic separation was performed in the solution containing 30 mM NaOH and 1 mM EDTA under alkaline conditions (pH > 13), at an ambient temperature of 4 °C (the temperature of the running buffer did not exceed 12 °C), for 20 min at an electric field strength of 0.73 V/cm (28 mA). Then, the slides were washed in water, drained, stained with 2 µg/mL DAPI, and covered with coverslips. To prevent additional DNA damage, the procedure was conducted under dark conditions. The comets were observed at 200× magnification using an Eclipse fluorescence microscope (Nikon, Japan) connected to a COHU 4910 video camera (Cohu, Inc., San Diego, CA, USA), which was equipped with a UV-1 A filter block and linked to a personal computer-based image analysis system, Lucia-Comet v. 7.3 (Laboratory Imaging, Praha, Czech Republic). One hundred images (comets) were randomly selected from each sample. A separate nucleoid consists of the undamaged part (the head) and the damaged part of DNA (the tail). The average DNA value in the comet tail was used as an indicator of DNA damage (expressed as a percentage). The longer and brighter the tail, the more severe the DNA damage.

## 4. Conclusions

In general, structural fusion of the chalcone core with the typical piperidinylpropoxy-H_3_R ligand motif resulted in dual ligands: H_3_R antagonists and MAO-B inhibitors. All hybrid compounds (**10**–**18**) had good affinity for hH_3_R with K_i_ values < 170 nM, while only three of them showed inhibitory activity for hMAO-B in the (sub)micromolar range (200 nM < IC_50_ < 1200 nM). Structure–activity relationship analysis indicated that a 3,4-dichloro substitution in the phenyl ring strongly enhanced hMAO-B inhibition, whereas a meta-benzyloxy attachment (opposed to a *para*-benzyloxy) in the piperidinyl chalcones favoured hH_3_R affinity. Among the synthesised hybrids, compound **15** was the best DTL with a K_i_ of 46.8 nM for hH_3_R and an IC_50_ of 212.5 nM for hMAO-B. Additional pharmacological studies of this compound confirmed its antagonist profile in the cAMP assay (K_d_ = 34.2 nM) and a competitive/mixed mode of reversible hMAO-B inhibition which makes possible the use of drugs such as compound **15** in the treatment of PD. 

The preliminary ADMET properties highlighted limited permeability and rapid metabolism, compound **15** also displayed marked antiproliferative (SH-SY5Y–72 h: IC_50_ = 3.44 μM) and genotoxic effects in cell-based assays, further suggesting its value for evaluation in anticancer studies. In many types of cancer (including colorectal cancer, glioblastoma, breast cancer, lung cancer, pancreatic cancer, and liver cancer), MAO-B overexpression is observed, suggesting its connection with cancer development [58,59]. Several MAO-B inhibitors with anticancer potential have been identified. In preclinical studies, they affected cell proliferation and apoptosis. Similarly, H_3_R overexpression occurs in cancer tissues compared to healthy tissues, i.e., in breast cancer, lung cancer, glioma, prostate cancer, and ovarian cancer. Some of the described H_3_R ligands inhibited cancer cell proliferation and affected their growth [60]. Thus, such dual activity may provide a beneficial effect in the treatment of cancer. Furthermore, many chalcones have demonstrated anticancer activity against various tumour cell lines by affecting different molecular processes associated with tumour formation and development [61]. Thus, the potential of compound **15** in CNS tumours should be further investigated in detail. Currently, therapeutic strategies are available that aim to destroy or inhibit tumour cells, including those of the nervous system, while minimising damage to normal neurons and other healthy cells. An example of such a drug is vorasidenib, which selectively inhibits only the mutated isocitrate dehydrogenase enzymes present in certain gliomas (affecting tumour growth) while sparing healthy cells that have the wild-type enzyme [62].

In this study, we described, for the first time, the use of the SPRM technique to study the kinetics of H_3_R ligands. Although it was not possible to perform such a study for compound **15** (due to its interactions with BSA), the other reference ligands (**DL76** and pitolisant) were evaluated. The calculated residence times for them were similar, around 16 min, suggesting that these compounds could be classified as not rapidly dissociating.

In conclusion, our research confirmed that hybrid chalcones are a promising starting scaffold for DTLs, as H_3_R antagonists, and MAO-B inhibitors, with potential utility not only in neurodegenerative diseases but also in anticancer therapy.

## Figures and Tables

**Table 1:** Structures and pharmacological results for evaluated chalcones **1**–**18** and reference compounds.

Comp.	X	hMAO-B ^a^ IC_50_ [nM]		Comp.	X	hH_3_R ^b^ K_i_ ± SEM [nM]	hMAO-B ^a^ IC_50_ ± SEM [nM] (% Inhibition) ^c^	hMAO-A ^a^ (% Inhibition) ^d^
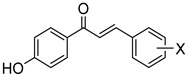				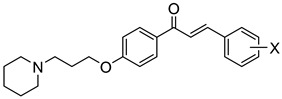				
**1**	H	96.0 ± 2.4 182 ± 5 ^f^		**10**	H	17.0 ± 2.2	(18%)	nt ^e^
**2**	4-Cl	2.67 ± 0.35 31 ± 1 ^g^		**11**	4-Cl	17.0 ± 0.5	(36%)	nt ^e^
**3**	3,4-diCl	0.85 ± 0.02		**12**	3,4-diCl	30.0 ± 0.9	500.0 ± 13.0	(3%)
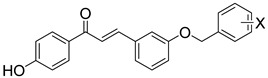				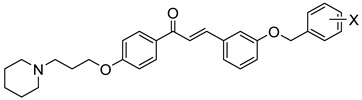				
**4**	H	85.2 ± 2.4		**13**	H	80.6 ± 9.0	(24%)	nt ^e^
**5**	4-Cl	33.4 ± 1.7		**14**	4-Cl	54.0 ± 6.0	1121.4 ± 198.7	(8%)
**6**	3,4-diCl	20.6 ± 1.3		**15**	3,4-diCl	46.8 ± 1.9	212.5 ± 12.3	(22%)
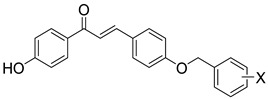				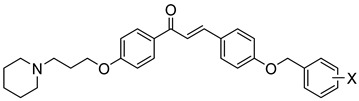				
**7**	H	287.5 ± 13.5		**16**	H	195.0 ± 13.0	(33%)	nt ^e^
**8**	4-Cl	337.5 ± 17.1		**17**	4-Cl	89.0 ± 4.0	1114.3 ± 317.0	(0%)
**9**	3,4-diCl	70.3 ± 4.5		**18**	3,4-diCl	163.0 ± 12.0	355.9 ± 19.9	(13%)
safinamide		8 ± 1						
rasagiline		25 ± 6						

^a^ Fluorometric MAO-Assay; mean value ± SEM of 2–4 independent experiments. ^b^ [^3^H] *N*^α^-Methylhistamine binding assay in CHO-K1 cells stably expressing the hH_3_R; mean value of 2 independent experiments ± SEM. ^c^ %Inhibition of hMAO-B at 1 μM; mean values of 2 independent experiments. ^d^ %Inhibition of hMAO-A at 10 μM; mean values of 2 independent experiments. ^e^ Not tested. ^f^ Data from Ref. [26]. ^g^ Data from Ref. [27].

**Table 2:** Affinity and kinetic values of **DL-76** and pitolisant binding to the hH_3_R ^1^.

Compound	Affinity	Kinetics			
	K_D_^a^ ± SEM [nM]	K_D_^b^ ± SEM [nM]	k_a_^c^ ± SEM [×10^4^ M^−1^ s ^−1^]	k_d_^d^ ± SEM [×10^−3^ s ^−1^]	RT ^e^ ± SEM [min]
**DL76**	39.2 ± 13.6	43.6 ± 12.6	2.7 ± 0.6	1.2 ± 0.3	16.0 ± 3.6
Pitolisant	15.0 ± 4.3	15.3 ± 4.7	7.9 ± 2.3	1.2 ± 0.2	15.8 ± 3.0

^1^ Mean values of 4–5 independence experiments. ^a^ Equilibrium constant from equilibrium binding experiment; ^b^ K_D_ = k_d_/k_a_; ^c^ association rate constant; ^d^ dissociation rate constant; ^e^ residence time (RT = 1/k_a_).

**Table 3:** Influence of different inhibition modalities on steady-state kinetic parameters and characteristic Lineweaver–Burk plot patterns of safinamide and compound **15**.

Parameter	Safinamide	Compound 15
Affinity	Free enzyme > enzyme–substrate complex	Only free enzyme or free enzyme > enzyme–substrate complex
app. K_M_	↑ curvilinearly with ↑ [I]	↑ curvilinearly with ↑ [I]
app. V_max_	↓ curvilinearly with ↑ [I]	↓ curvilinearly with ↑ [I]
app. V_max/_app. K_M_	↓ curvilinearly with ↑ [I]	↓ curvilinearly with ↑ [I]
Lines on LB plot	Lines intersect to the left of the *y*-axis and above the *x*-axis	Lines intersect to the left of the *y*-axis and above the *x*-axis (almost on *y*-axis)
Mode of inhibition from kinetic values and LB plot	Mixed mode	Competitive/Mixed mode

[I]—concentration of the inhibitor, K_M_—Michaelis constant, V_max_—maximum velocity obtained at infinite substrate concentration, LB—Lineweaver-Burk double-reciprocal plot. ↑—increase, ↓—decrease.

**Table 4:** Results from the Caco-2 permeability assay.

Compound	Apparent Permeability (P_app_)		Efflux Ratio (ER) ^a^
	P_app_ (A − B) ×10^−6^ cm/s ± SD	P_app_ (B − A) ×10^−6^ cm/s ± SD	
**12**	0.75 ± 0.05	0.52 ± 0.03	0.70
**15**	0.14 ± 0.02	0.06 ± 0.03	0.43
Caffeine	30.16 ± 2.94	34.80 ± 10.88	1.08

^a^ Efflux ratio calculation: ER = P_app_ (B − A)/P_app_ (A − B). An efflux ratio less than 2 suggests passive diffusion is the primary mode of transport. An efflux ratio greater than 2 indicates active transport, likely involving efflux pumps.

**Table 5:** In vitro metabolic stability and the most probable metabolic pathways of compounds **12** and **15** after incubation for 2 h with rat liver microsomes.

Substrate		Metabolite		
Molecular Mass (*m*/*z*)	Retention Time (min)	Molecular Mass (*m*/*z*)	Retention Time (min)	Metabolic Pathway
**12** (418.36)	7.25	434.14	7.27	hydroxylation
**15** (524.19)	8.22	540.22	8.22	hydroxylation

**Table 6:** In vitro Metabolic stability of compound **15** and Verapamil (reference unstable drug) after incubation for 2 h in mouse liver microsomes.

Compound	Cl_int_^a^ (mL/min/kg)	t_0.5_^b^ (min)	Metabolic Stability
**15**	285.75	18.99	weak/unstable
Verapamil	239.5	22.60	unstable

^a^ Intrinsic clearance; ^b^ half-life.

**Table 7:** IC_50_ values obtained for compound **15** against PBMCs and SH-SY5Y (neuroblastoma) cells.

Incubation Time	IC_50_ (μM)	
	PBM Cells	SH-SY5Y Cells
2 h	80.30	(-)
24 h	20.99	4.46
48 h	14.25	(-)
72 h	11.97	3.44

IC_50_ values were determined after 2–72 h of incubation of PBMCs or SH-SY5Y cells with compound **15** at concentrations ranging from 0.195 to 100 μM (4 experiments) and 0.39 to 25 μM (6 experiments), respectively. (-)—not analysed.

## Data Availability

The original contributions presented in this study are included in the article/supplementary material. Further inquiries can be directed to the corresponding author(s).

## Supplementary Materials

The supporting data can be downloaded at: https://www.mdpi.com/article/10.3390/ijms27020581/s1.

## Abbreviations

The following abbreviations are used in this manuscript:

ADHD	Attention Deficit Hyperactivity Disorder
ADMET	Absorption, Distribution, Metabolism, Excretion, Toxicity
BBB	blood–brain barrier
BSA	bovine serum albumin
Caco-2	human colorectal cancer cell line
Cl_int_	Clearance
cAMP	3′,5′-cyclic adenosine monophosphate
CYP	cytochrome
DA	dopamine
DCM	dichloromethane
DTLs	dual-target ligands
DX	doxorubicin
ER	efflux ratio
FAD	flavin adenine dinucleotide
FC	flash chromatography
HepG2	human liver cancer cell line
H_3_R	histamine H_3_ receptor
hH_3_R	human histamine H_3_ receptor
hMAO-A	human monoamine oxidase A
hMAO-B	human monoamine oxidase B
KE	ketoconazole
MAO-B	monoamine oxidase B
MLMs	mouse liver microsomes
MM-GBSA	Molecular Mechanics–Generalized Born Surface Area
NC	negative controls; untreated control
PAMPA	Parallel Artificial Membrane Permeability Assay
P_app_	permeability coefficient
PBMCs	peripheral blood mononuclear cells
PBS	phosphate-buffered saline
PC	positive control
PD	Parkinson’s Disease
PEA	β-phenylethylamine
PFA	paraformaldehyde
RLMs	rat liver microsomes
RMSD	Root Mean Square Deviation
ROS	reactive oxygen species
RT	residence time
SAF	Safinamide
SH-SY5Y	human neuroblastoma cell line
SPRM	Surface Plasmon Resonance Microscopy
QD	quinidine
