# Supplementary material for: Rational Design, Synthesis and Pharmacological Evaluation of Chalcones as Dual-Acting Compounds—Histamine H3 Receptor Ligands and MAO-B Inhibitors
Source: Int J Mol Sci. 2026 Jan 6;27(2):581. doi: 10.3390/ijms27020581 (PMC12840679; doi:10.3390/ijms27020581)
Supplement: Supplementary file 1 [file ijms-27-00581-s001.zip › ijms-4056178-supplementary/Supplementary data/Supplementary S1.pdf]

Rational design, synthesis and pharmacological evaluation of chalcones as dual-acting compounds – histamine H_3 receptor ligands and MAO B inhibitors

Dorota Łażewska[1*], Agata Doroz-Płonka[1], Kamil Kuder[1], Agata Siwek[2,3], Waldemar Wagner[4,5], Joanna Karnafał-Ziembla[6], Agnieszka Olejarz-Maciej[1], Małgorzata Wolak[2], Monika Głuch-Lutwin[2], Barbara Mordyl[2], Oktawia Osiecka[2], Michał Juszczak[7], Katarzyna Woźniak[7], Małgorzata Więcek[1], Gniewomir Latacz[1,6], Anna Stasiak[4*]

[1]Department of Chemical Technology and Biotechnology of Drugs, Faculty of Pharmacy, Jagiellonian University Medical College in Kraków, Medyczna 9, 30-688 Kraków, Poland
[2]Department of Pharmacobiology, Faculty of Pharmacy, Jagiellonian University Medical College in Kraków, Medyczna 9, 30-688 Kraków, Poland
[3]Radioisotope and Functional Analysis Laboratory, Center for the Development of Therapies for Civilization and Age-Related Diseases, Jagiellonian University Medical College in Kraków, Medyczna 7A, 30-688 Kraków, Poland
[4]Department of Hormone Biochemistry, Faculty of Medicine, Medical University of Lodz, Żeligowskiego 7/9,90-752 Łódź, Poland
[5]Laboratory of Cellular Immunology, Institute of Medical Biology Polish Academy of Sciences, Lodowa 106, 93-232 Łódź, Poland
[6]Pharmacokinetics and Preliminary Toxicological Analysis Laboratory, Center for the Development of Therapies for Civilization and Age-Related Diseases, Jagiellonian University Medical College in Kraków, Medyczna 7A, 30-688 Kraków, Poland
[7]Department of Molecular Genetics, Faculty of Biology and Environmental Protection, University of Lodz, Pomorska 141/143, 90-236 Łódź, Poland

Correspondence: dorota.lazewska@uj.edu.pl (D.Ł.); anna.stasiak@umed.lodz.pl (A.St.)

Table of contents

Graphs showing the data that were used to calculate the IC_{50} values in the case of MAO B ………………………………………………………………………………………… **3-6**

Data from functional characterisation in cAMP accumulation assay of compounds **12** and **15** …..…………..……………………………………………………………… **7**

Figures S1-S7. Metabolic stability evaluation ………………………..……………..…**8-12**

Tables S1-S4. Views of microscope images taken for compounds **12** and **15** at various concentrations on the tested cell lines ……………………….………………………..**13-18**

Figures S8-S16. ^{1}H NMR spectra of compounds **1-9** ……………………………………**19-24**

Figures S17-S34. ^{1}H and ^{13}C NMR spectra of compounds **10-18** ……………………..…**25-33**

Figures S35-S52. Purity assessment of compounds **1 -18** determined by LC-MS……….**34-51**

Graphs showing the data that were used to calculate the IC_{50} values in the case of MAO B.

compound	curve
1	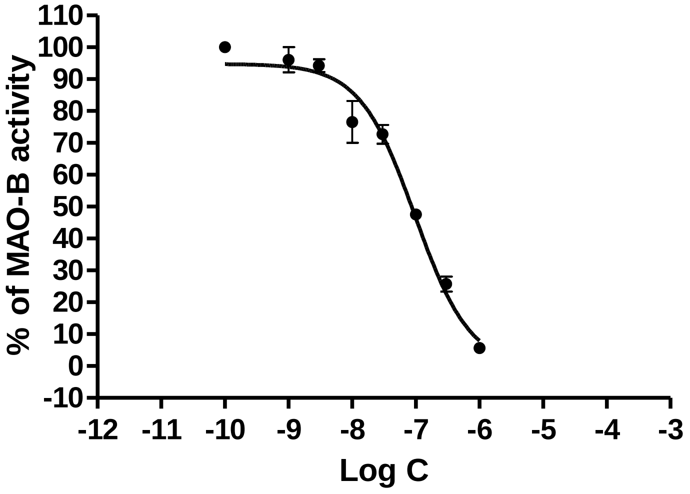
2	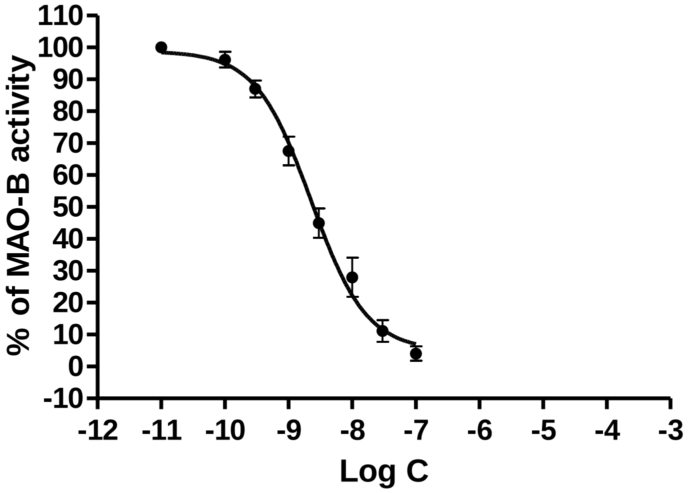
3	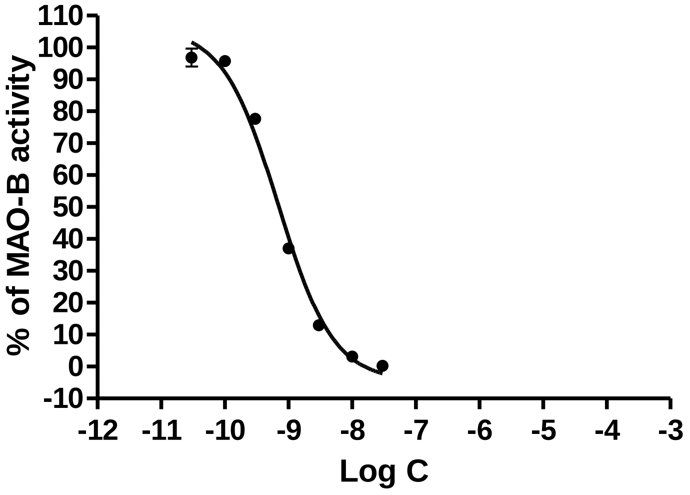

4	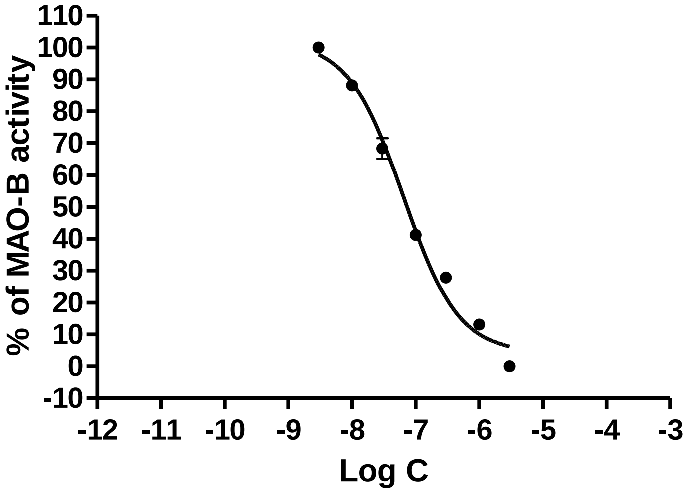% of MAO-B activity<br>110<br>100<br>90<br>80<br>70<br>60<br>50<br>40<br>30<br>20<br>10<br>0<br>-10<br>-12<br>-11<br>-10<br>-9<br>-8<br>-7<br>-6<br>-5<br>-4<br>-3<br>Log C
5	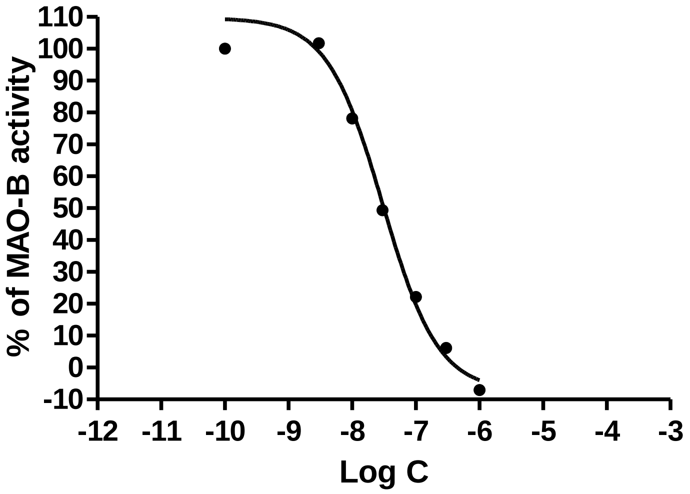% of MAO-B activity<br>110<br>100<br>90<br>80<br>70<br>60<br>50<br>40<br>30<br>20<br>10<br>0<br>-10<br>-12<br>-11<br>-10<br>-9<br>-8<br>-7<br>-6<br>-5<br>-4<br>-3<br>Log C
6	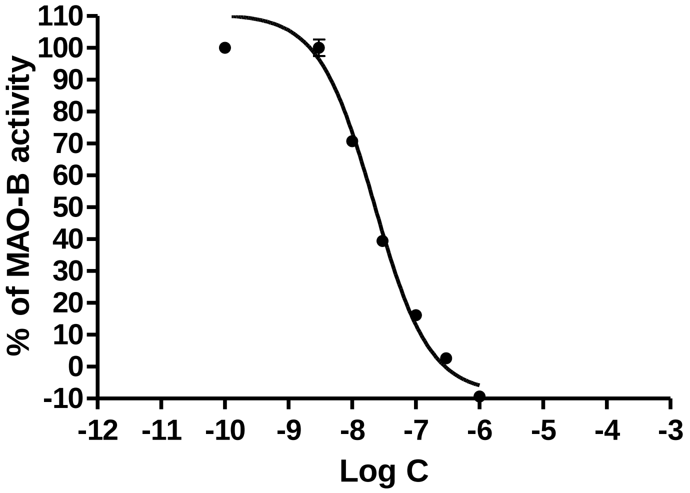% of MAO-B activity<br>110<br>100<br>90<br>80<br>70<br>60<br>50<br>40<br>30<br>20<br>10<br>0<br>-10<br>-12<br>-11<br>-10<br>-9<br>-8<br>-7<br>-6<br>-5<br>-4<br>-3<br>Log C

7	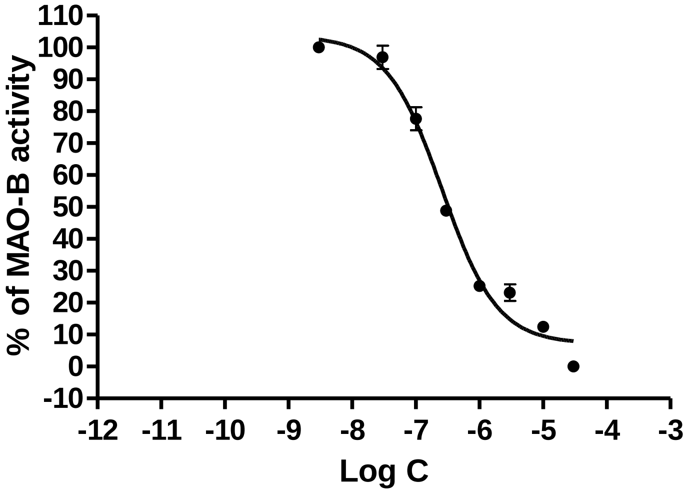% of MAO-B activity<br>Log C<br>110<br>100<br>90<br>80<br>70<br>60<br>50<br>40<br>30<br>20<br>10<br>0<br>-10<br>-12<br>-11<br>-10<br>-9<br>-8<br>-7<br>-6<br>-5<br>-4<br>-3
8	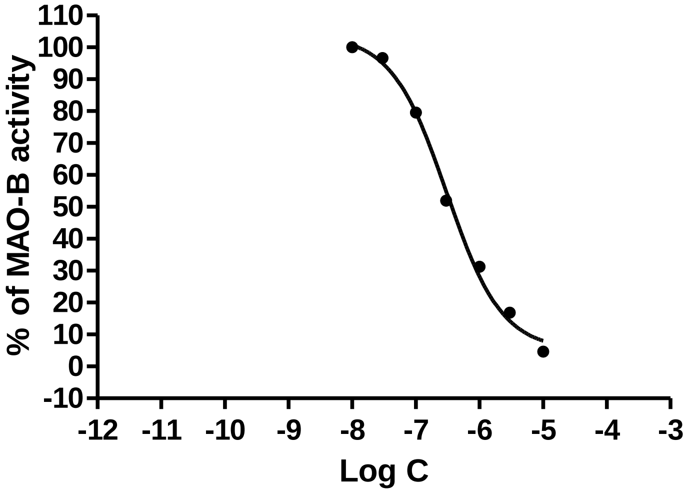% of MAO-B activity<br>Log C<br>110<br>100<br>90<br>80<br>70<br>60<br>50<br>40<br>30<br>20<br>10<br>0<br>-10<br>-12<br>-11<br>-10<br>-9<br>-8<br>-7<br>-6<br>-5<br>-4<br>-3
9	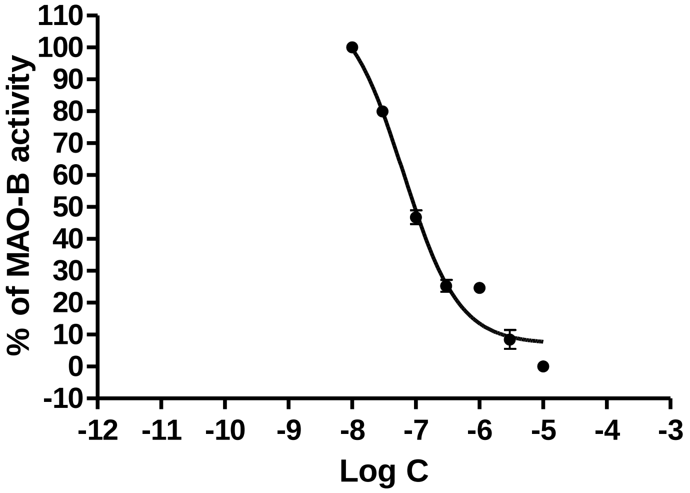% of MAO-B activity<br>Log C<br>110<br>100<br>90<br>80<br>70<br>60<br>50<br>40<br>30<br>20<br>10<br>0<br>-10<br>-12<br>-11<br>-10<br>-9<br>-8<br>-7<br>-6<br>-5<br>-4<br>-3

<table>
<tr><td>12</td><td>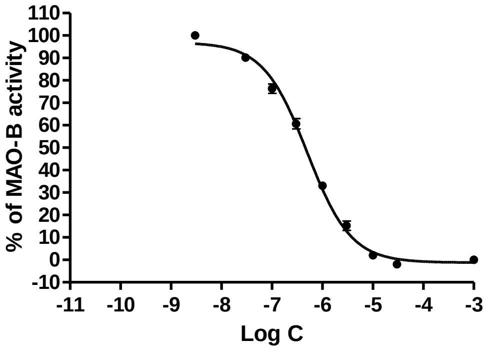
</td></tr>
<tr><td>15</td><td>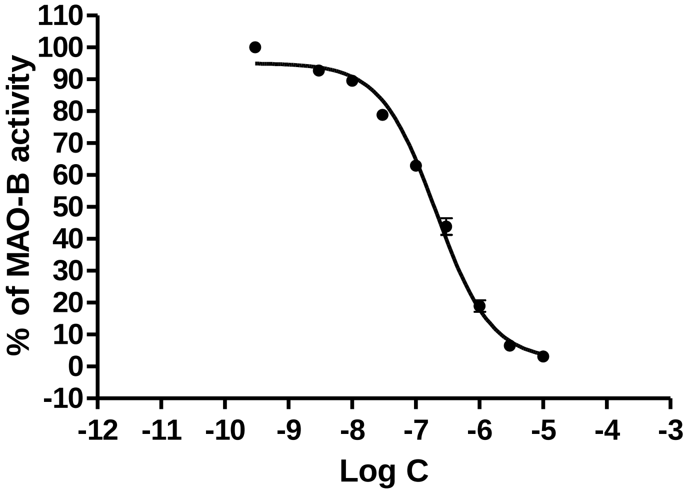
</td></tr>
<tr><td>17</td><td>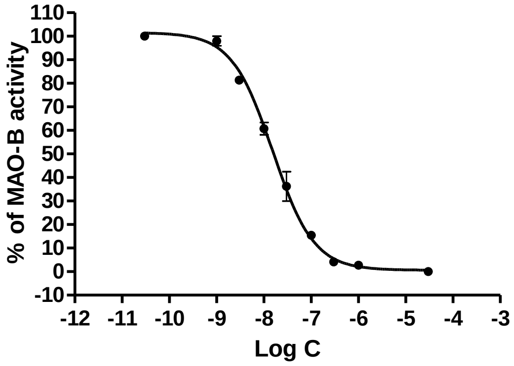
</td></tr>
<tr><td>18</td><td>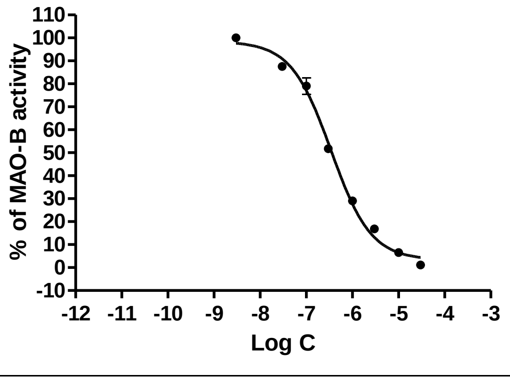
</td></tr>
</table>

Data from functional characterisation in cAMP accumulation assay of compounds 12 and 15[1]

cAMP H_3R													
Agonist mode*					Antagonist mode**								
Compound	E max %	EC_{50}	pEC_{50}	SD	Compound	E max %	IC_{50}	IC_{50}	pIC_{50}	K_b	K_b	SD	R2
		M		pEC_{50}			M	nM		M	nM	Kb [nM]	Kb
**α-Methylhistamine**	100	5.93E-09	8.23	0.873	**Thioperamide**	100	1.11E-07	110	6.96	4.14E-08	41.4	2.81	0.931
**12**	0	n.c.	n.c.	n.c.	**AR-15**	109	1.05E-08	10.5	7.98	3.94E-09	3.94	0.85	0.974
**15**	3	n.c.	n.c.	n.c.	**AR-35**	105	6.30E-08	63	7.20	3.42E-08	34.2	2.78	0.953

[1]Compounds were tested in duplicate in two separate experiments *Results were normalized as percentage of maximal agonist response (α-Methylhistamine10^{-5} M); **Results were normalised as a percentage of reference antagonist (Thioperamide 10^{-5} M); E_{max} is the maximum possible effect; n.c. - not calculable;

Metabolic stability evaluation.

1. ***In silico* studies**

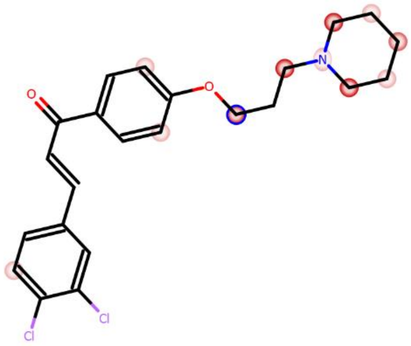

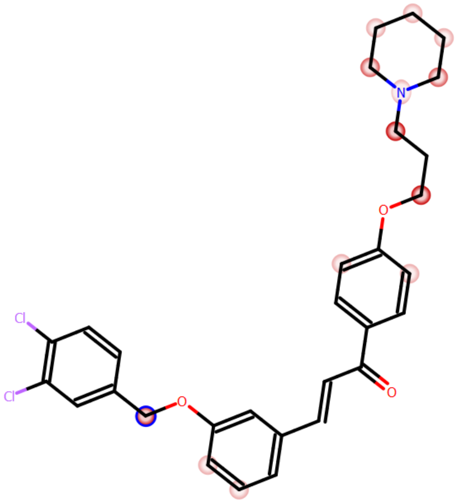

Figure S1. In silico prediction of the sites of metabolism by MetaSite 6.01 for compounds: **12** (left) and **15** (right). The darker red color - the higher probability to be involved in the metabolism pathway. The blue circle marked the site of compound with the highest probability of metabolic bioconversion.

2. ***In vitro* – determination of metabolic pathways in rat liver microsomes (RLMs)**

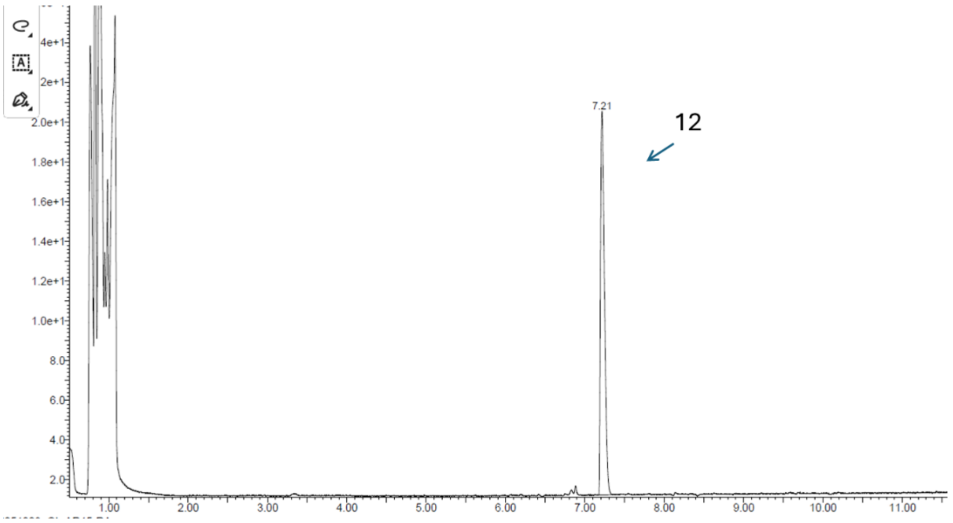

Figure S2. UPLC of control reaction of compound **12** (incubation for 120 min without microsomes)

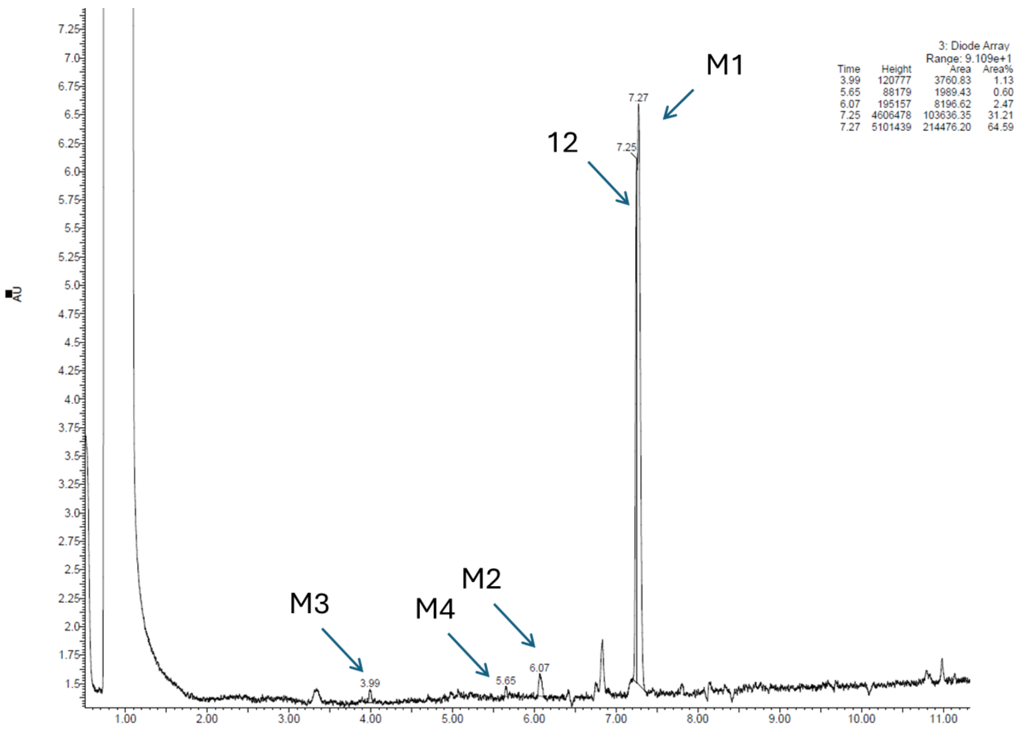

Figure S3. UPLC after incubation of **12** for 120 min with rat liver microsomes.

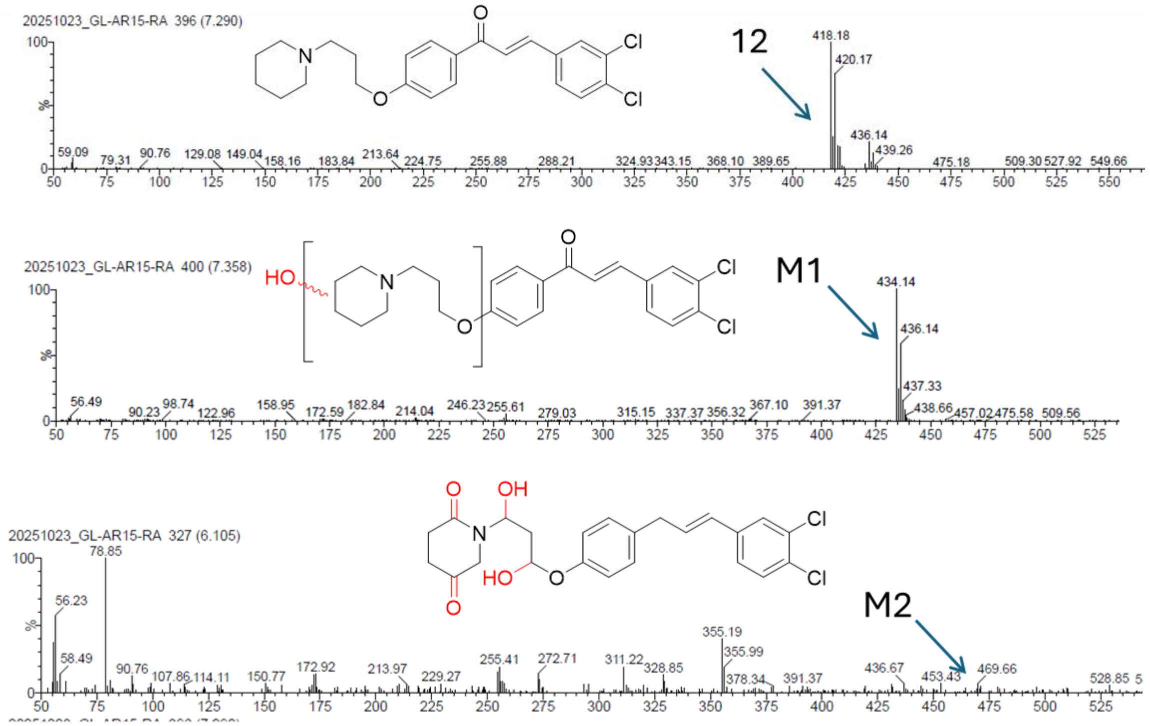

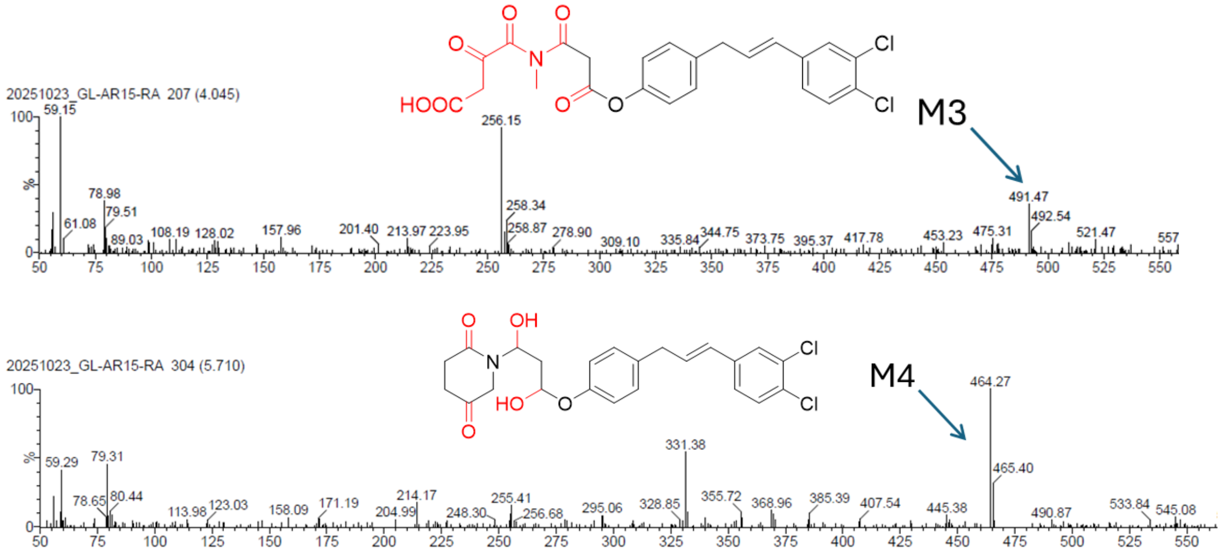

Figure S4. MS spectra of **12** and metabolites after incubation for 120 min with rat liver microsomes.

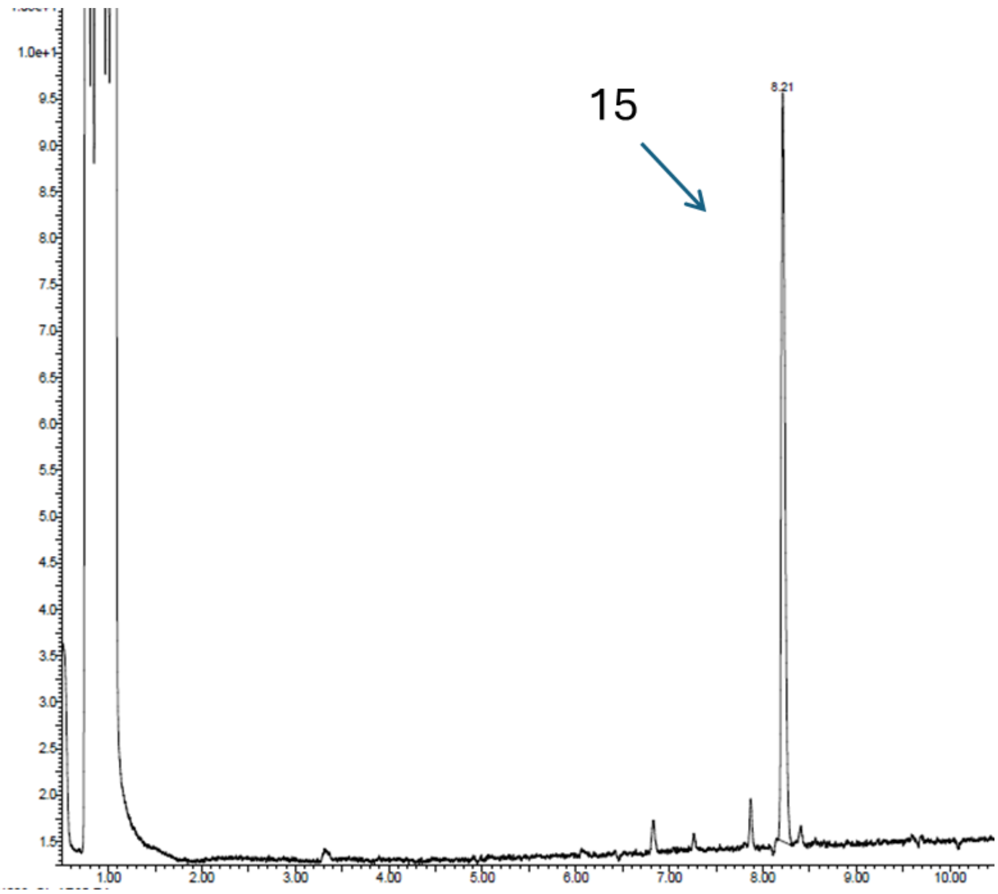

Figure S5. UPLC of control reaction of compound **15** (incubation for 120 min without microsomes)

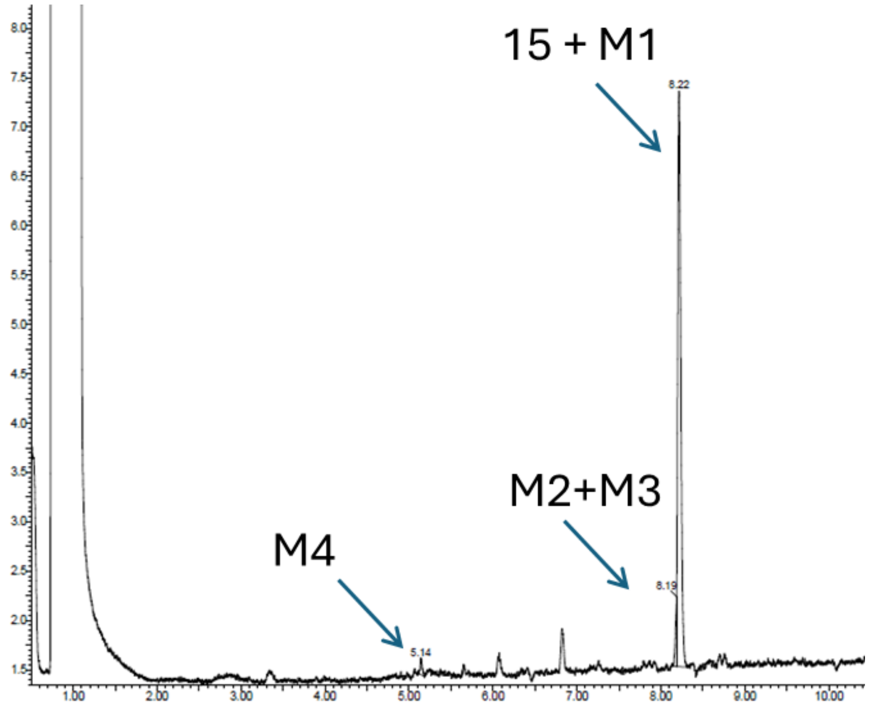

Figure S6. UPLC after incubation of **15** for 120 min with rat liver microsomes.

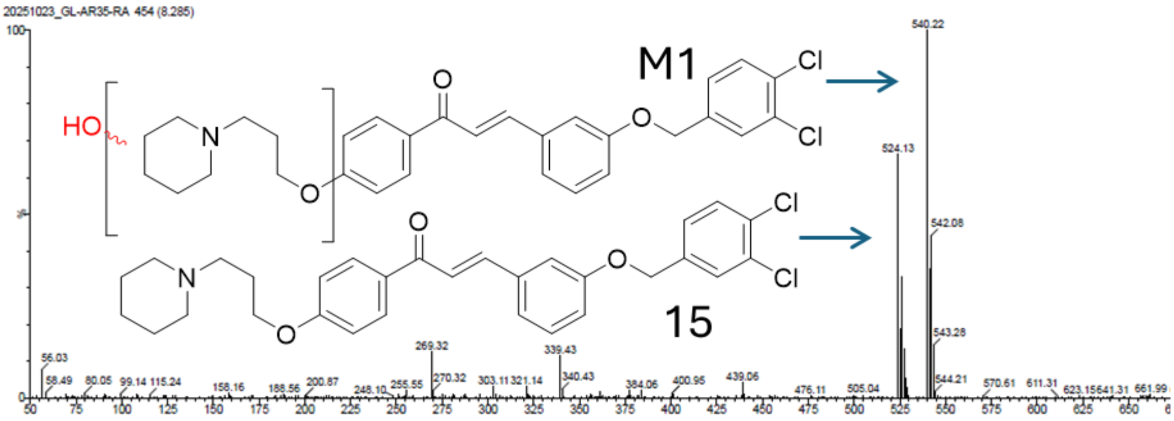

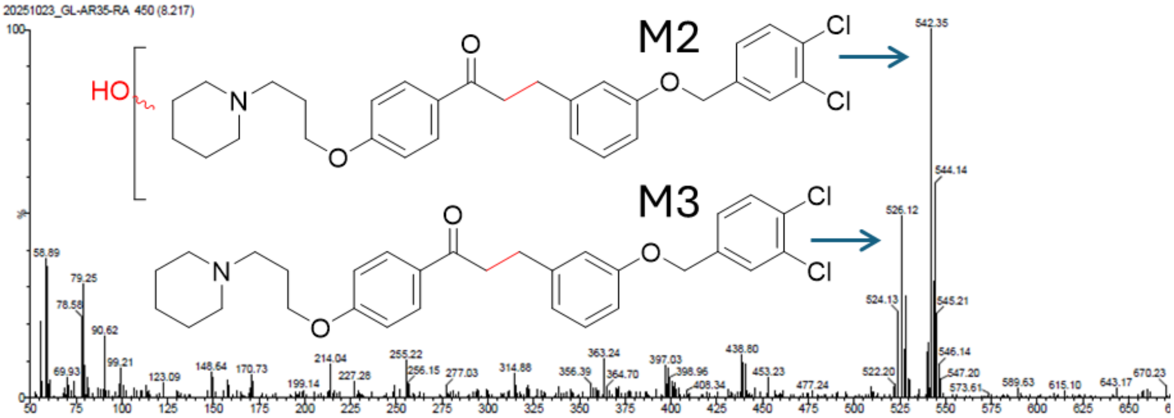

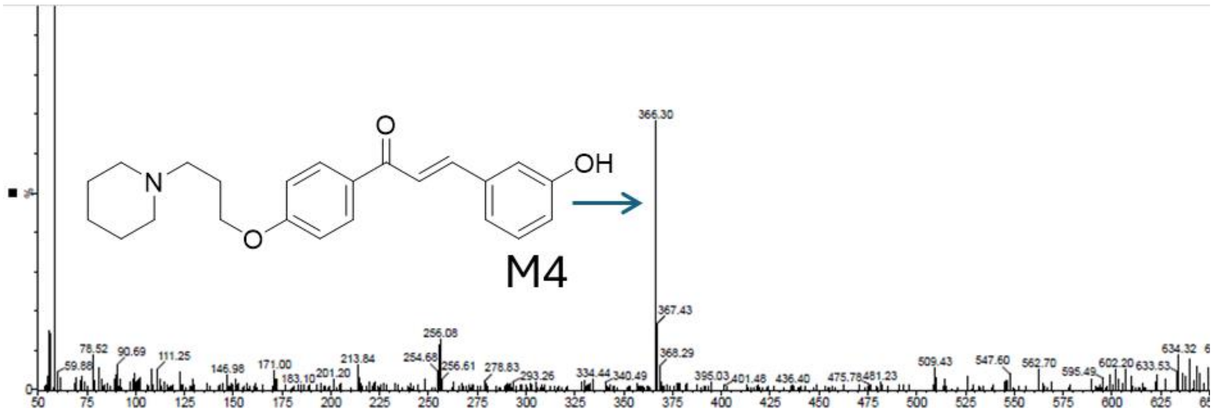

Figure S7. MS spectra of **15** and metabolites after incubation for 120 min with rat liver microsomes.

Views of microscope images taken for compounds 12 and 15 at various concentrations on the tested cell lines. The photomicrographs were done using Leica DMi8 inverted microscope (objective 10x) of the HepG2 and SH-SY5Y cells after 72h of incubation in the presence of compounds **12** and **15** (0.1-100 μM).

Table .S1 The microscope images of SH-SY5Y cells after incubation with compound **12** at various concentrations. Compound precipitated at 25 -100 μM.

**Compound 12**	
0.1 μM	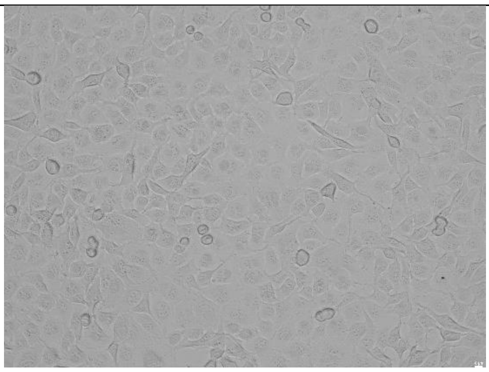 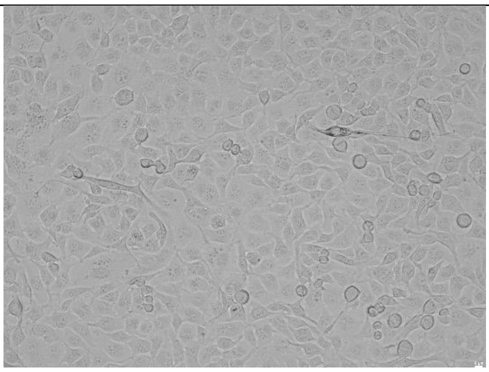
1 μM	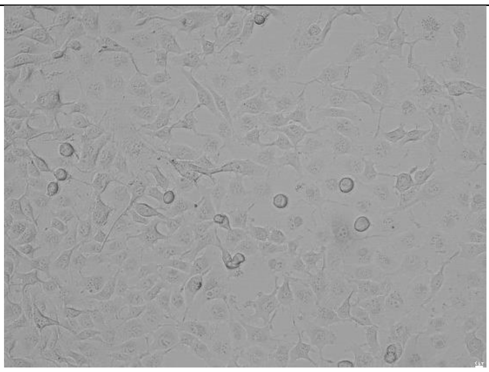 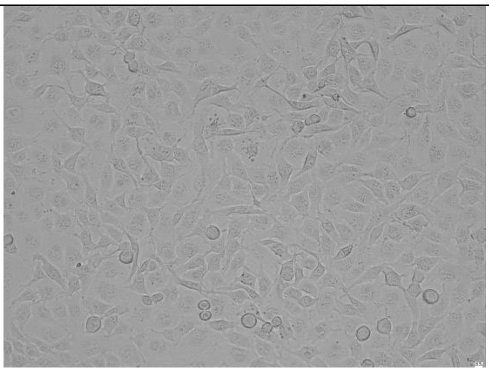
5 μM	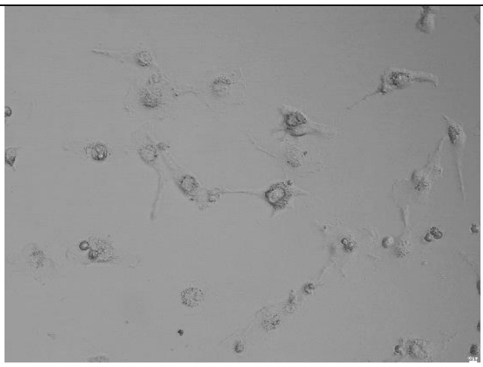 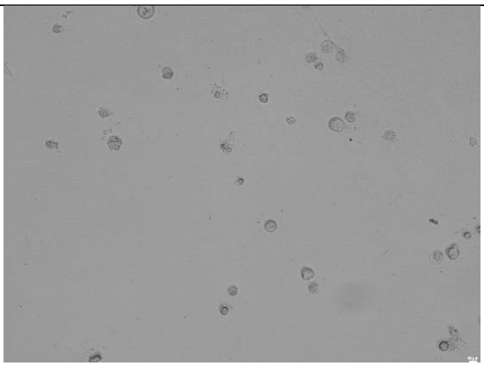

10 µM	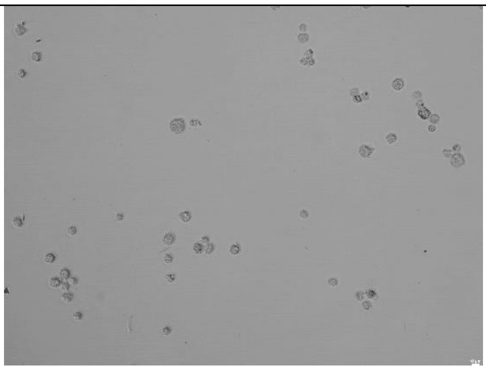 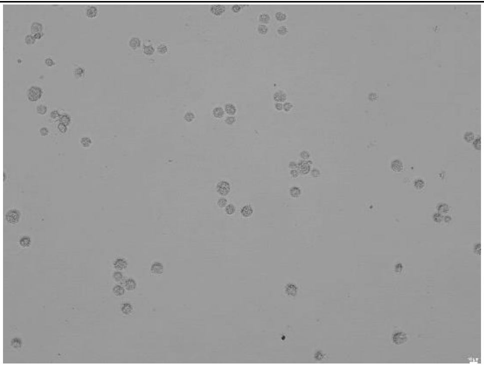
25 µM	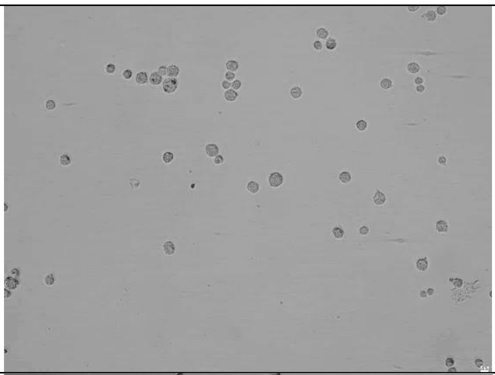 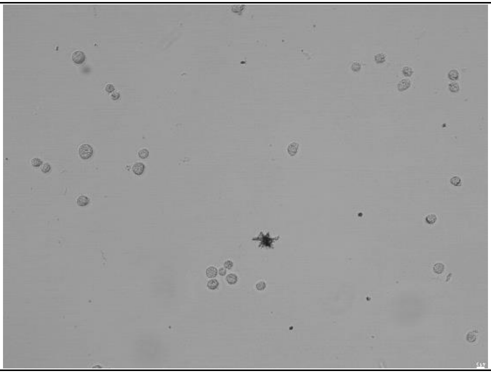
50 µM	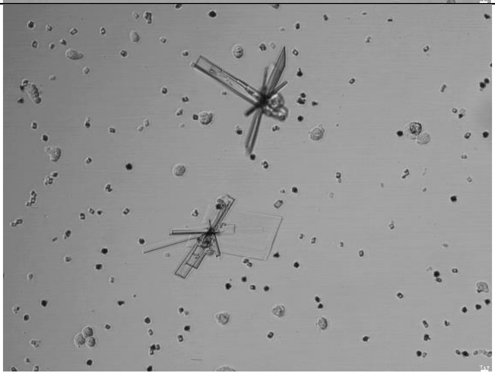 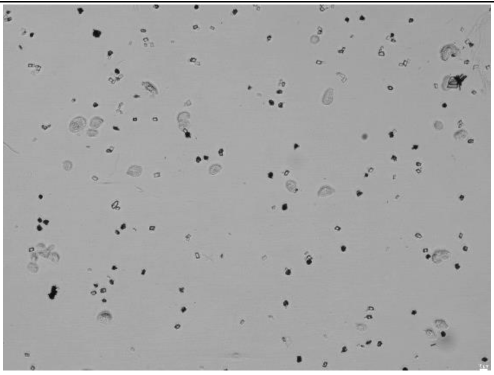
100 µM	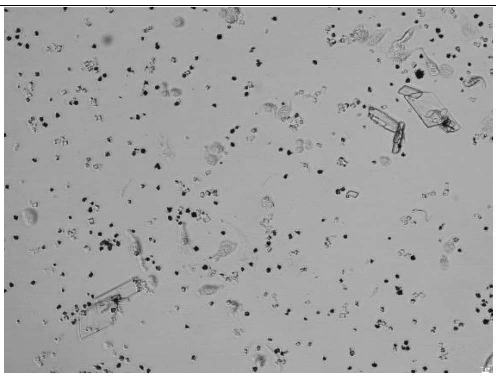 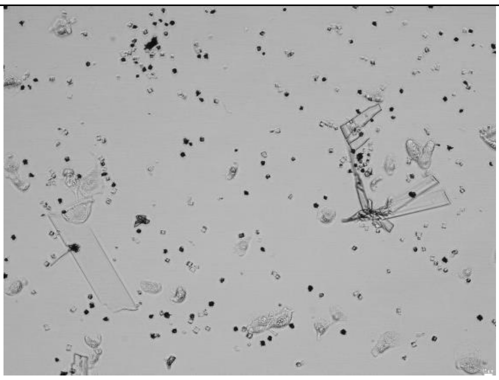

kontrola	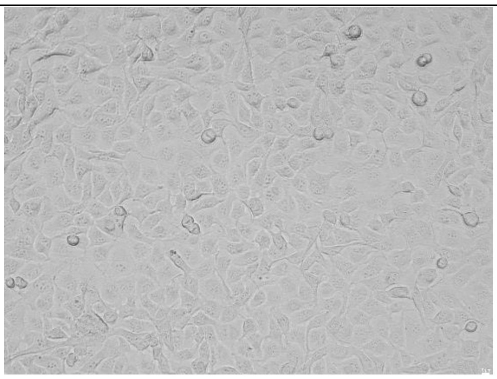 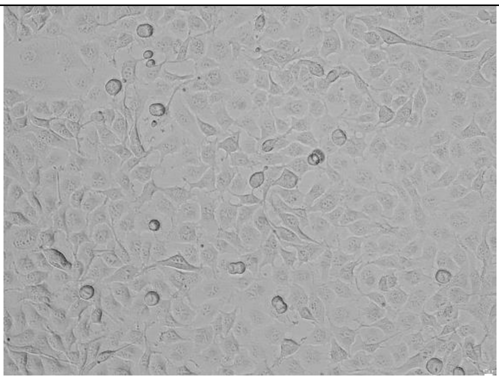

Table S2. The microscope images of SH-SY5Y cells after incubation with compound **15** at various concentrations. Compound precipitated at 50 and 100 µM.

Compound **15**	
0,1 µM	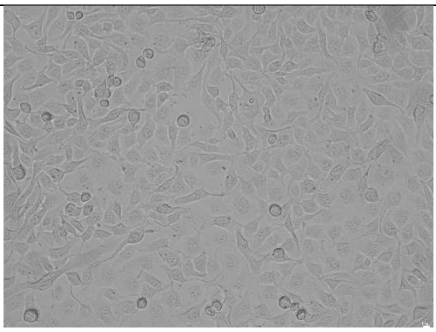 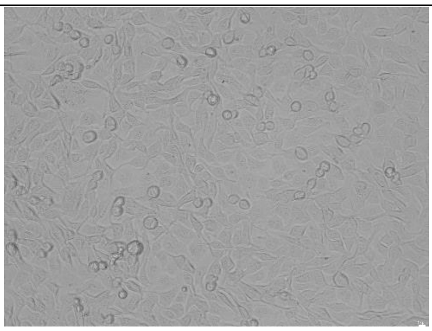
1 µM	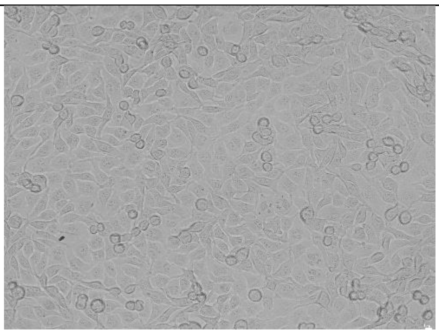 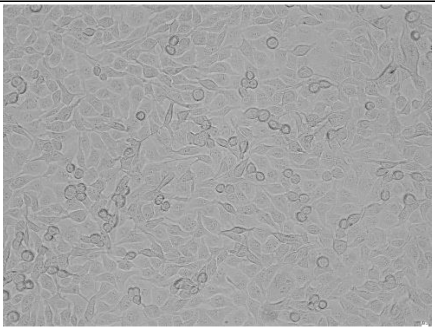
10 µM	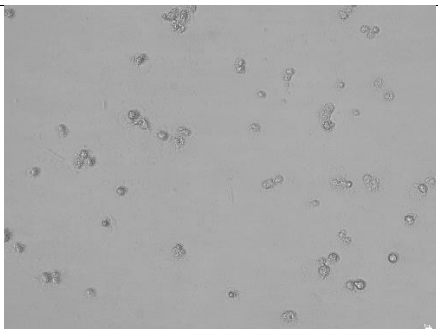 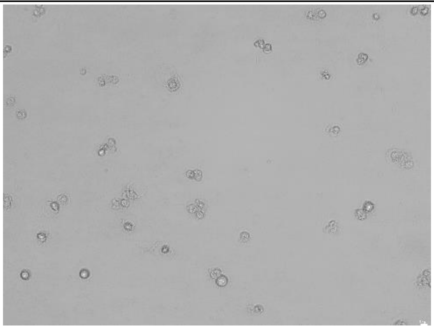

25 Mm	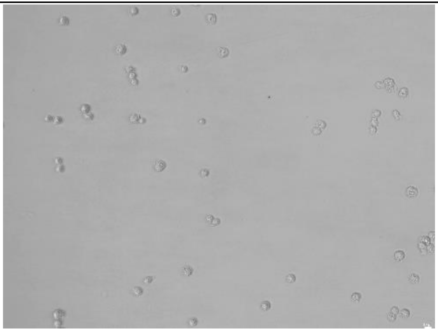 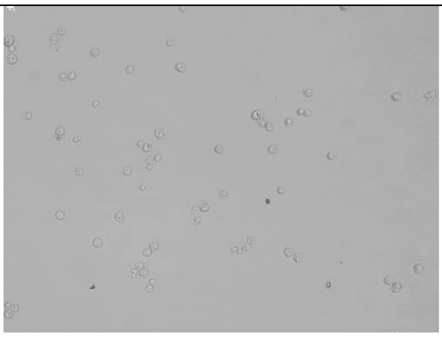
50 µM	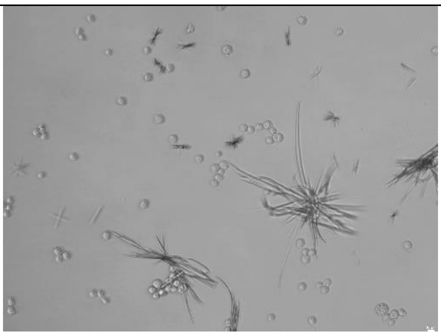 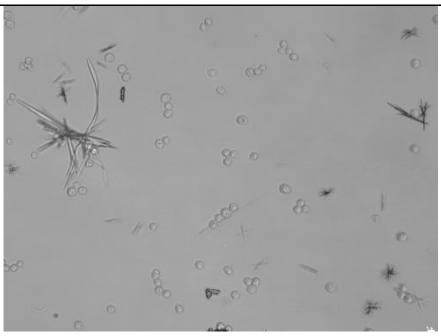
100 µM	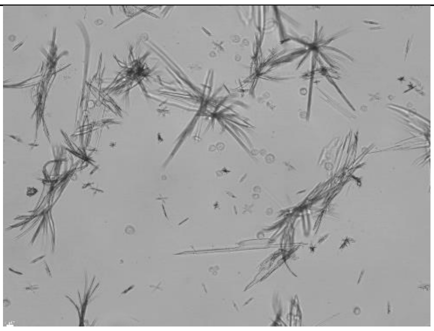 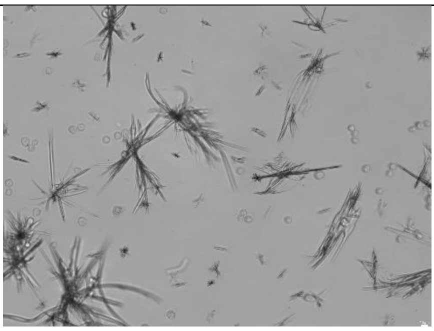
kontrola	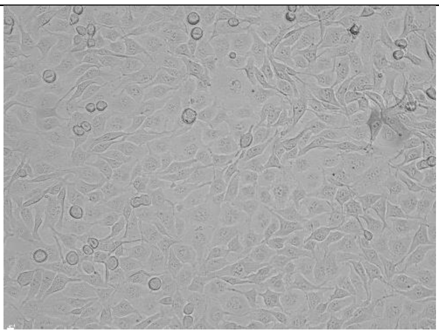 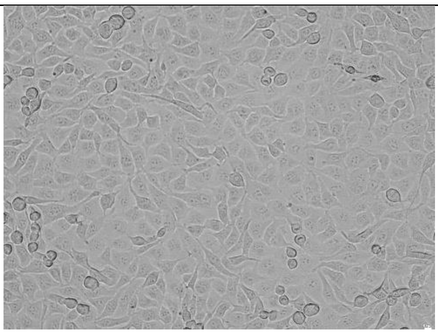

Table S3. The microscope images of HepG2 cells after incubation with compound **12** at various concentrations. Compound precipitated at 25 -100 µM.

Compound **12**	
0,1 µM	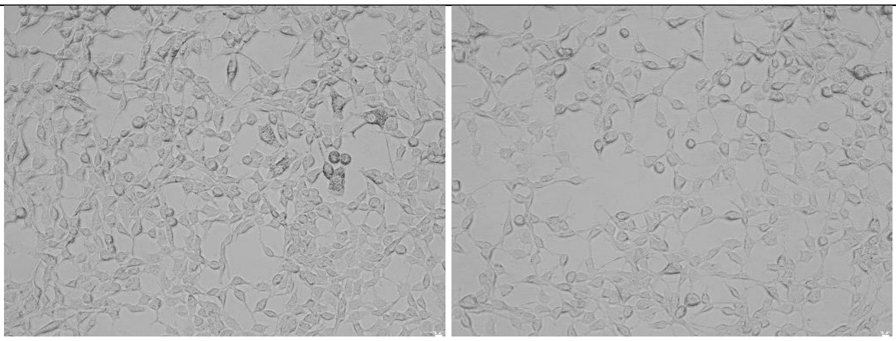
1 µM	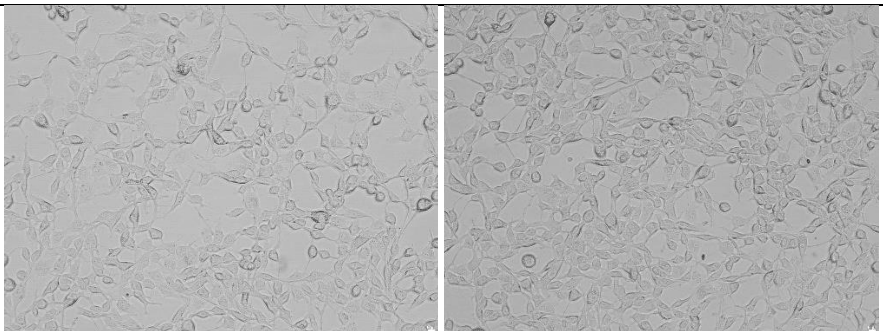
10 µM	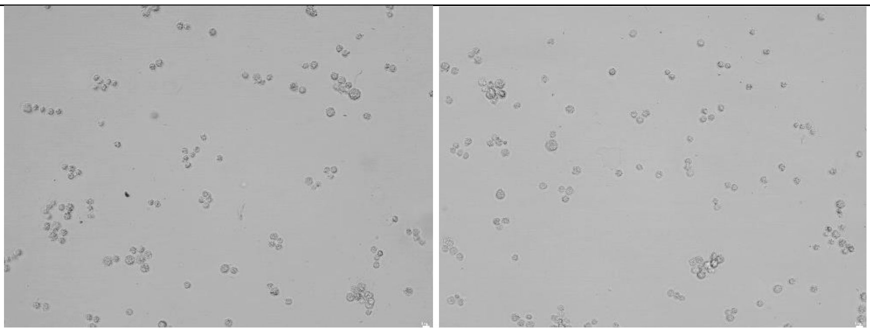
25 µM	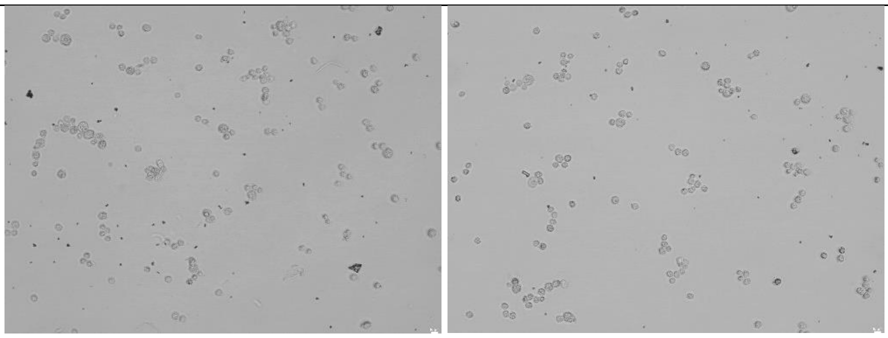

50 µM	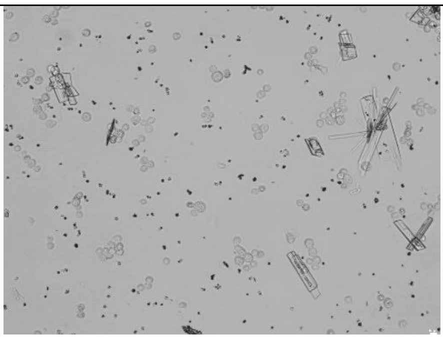 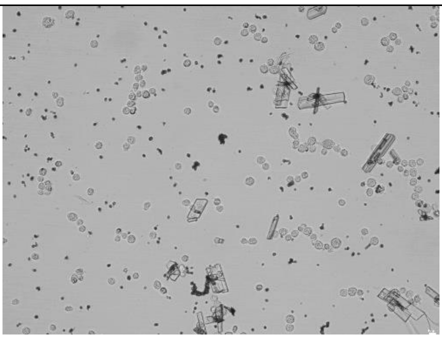
100 µM	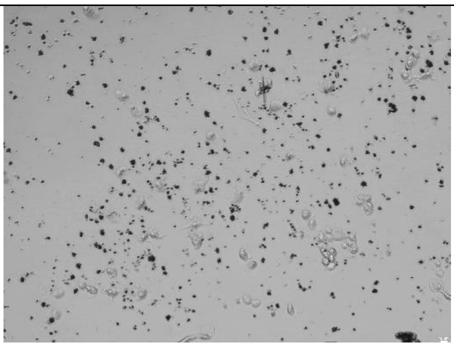 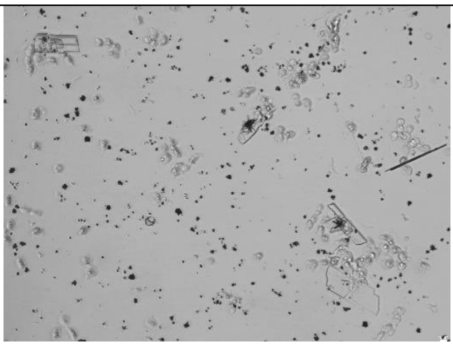
kontrola	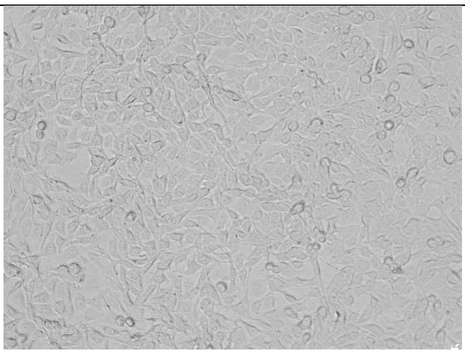 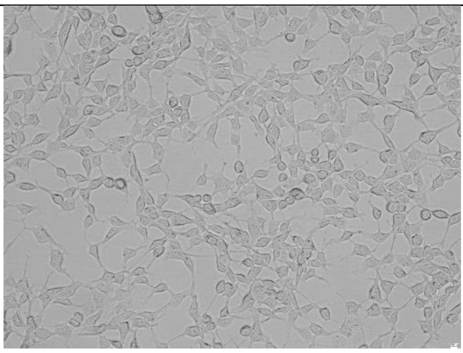

Table S4. The microscope images of HepG2 cells after incubation with compound **15** at various concentrations. Compound precipitated at 50 and 100 µM.

Compound **15**	
0,1 µM	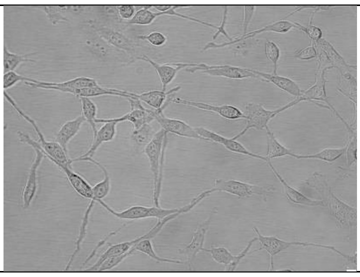

1 µM	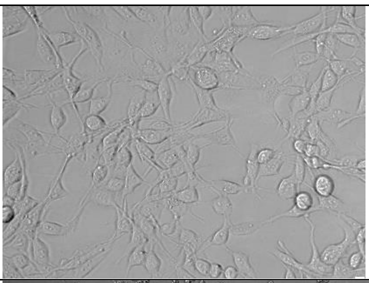
10 µM	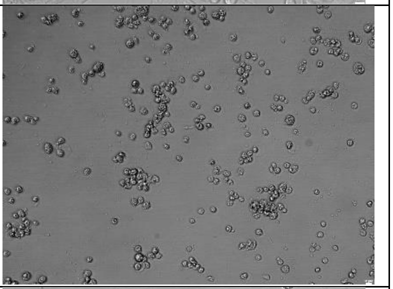
25 µM	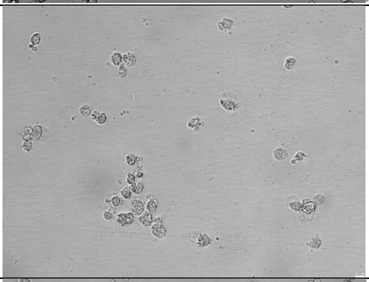
50 µM	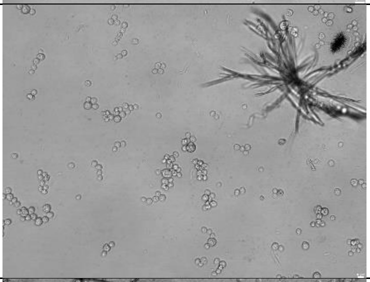
100 µM	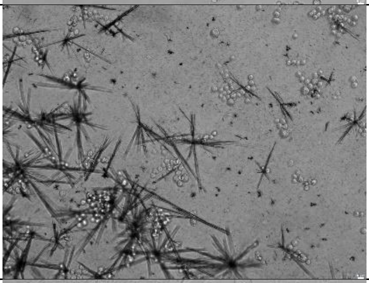
Control	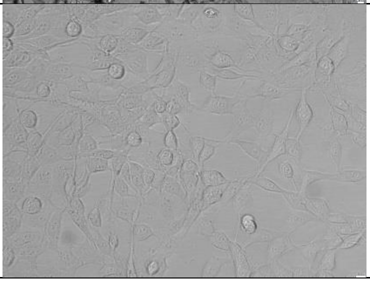

^{1}H NMR spectra of compounds 1-9.

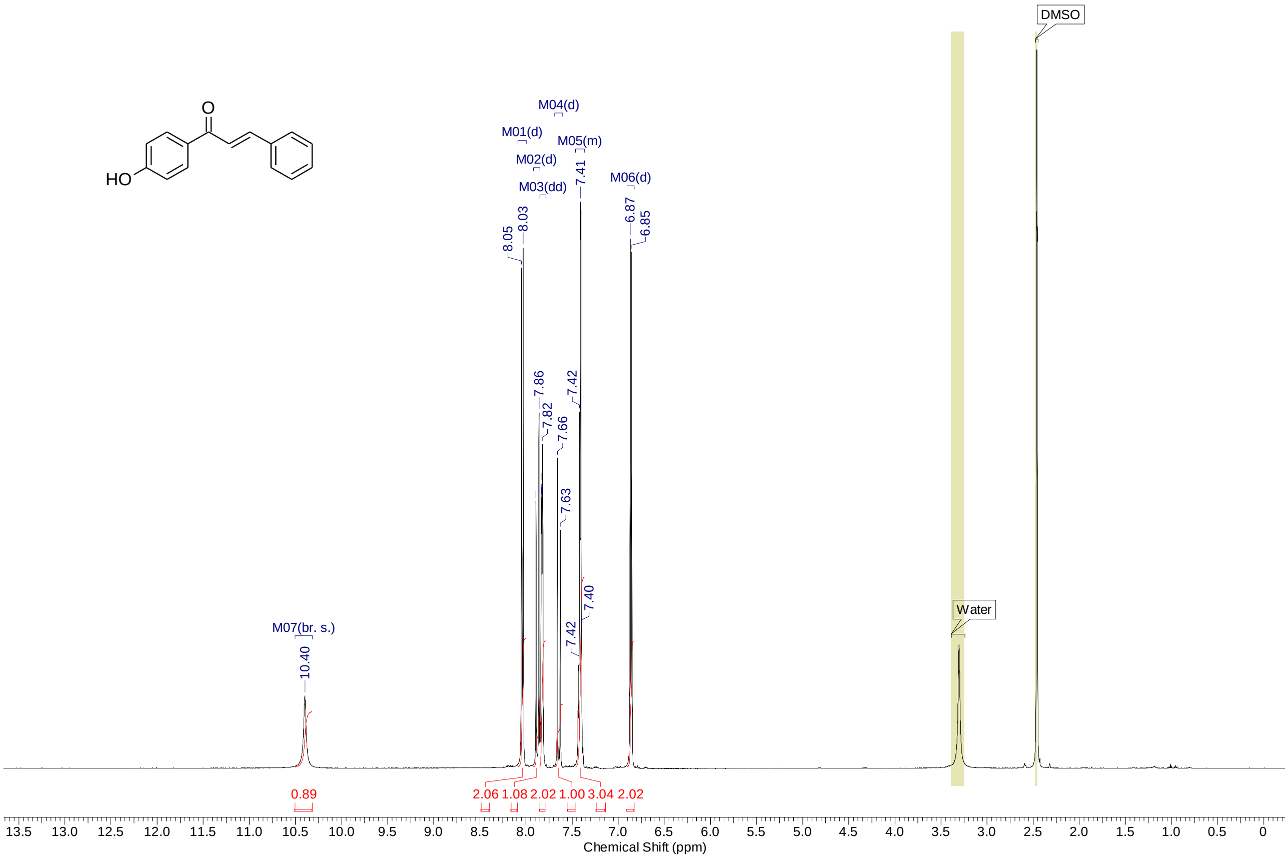

Figure S8. ^{1}H NMR spectrum of compound **1**.

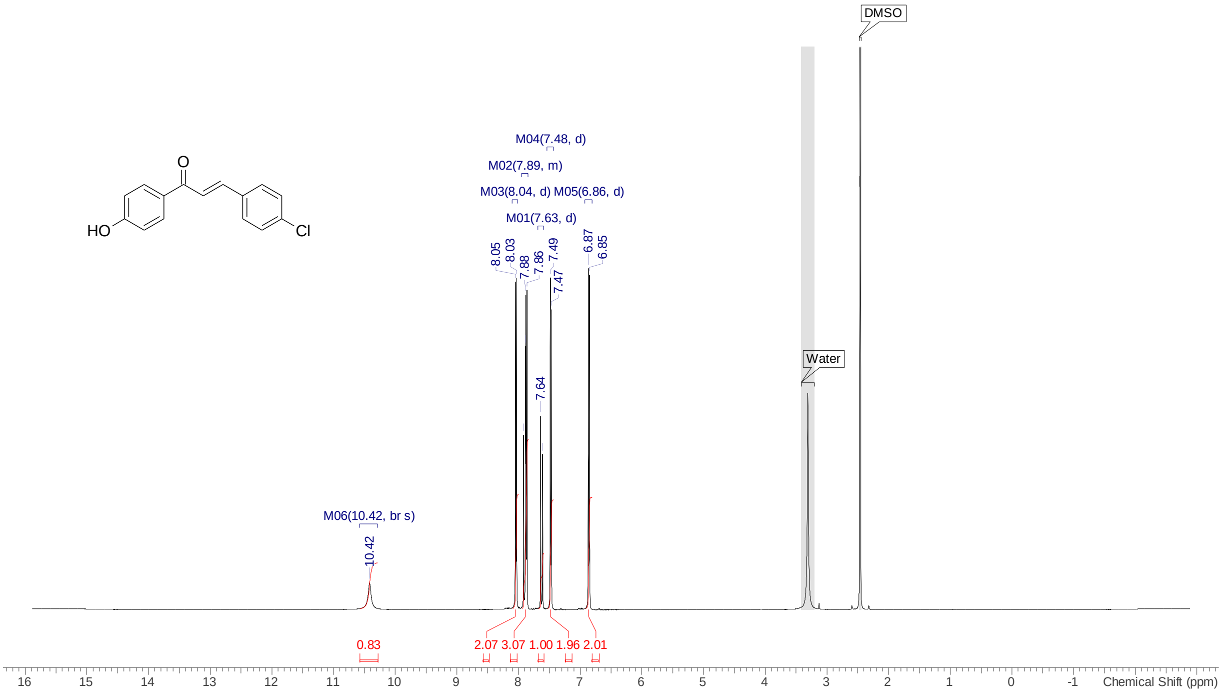

Figure S9. ^{1}H NMR spectrum of compound **2**.

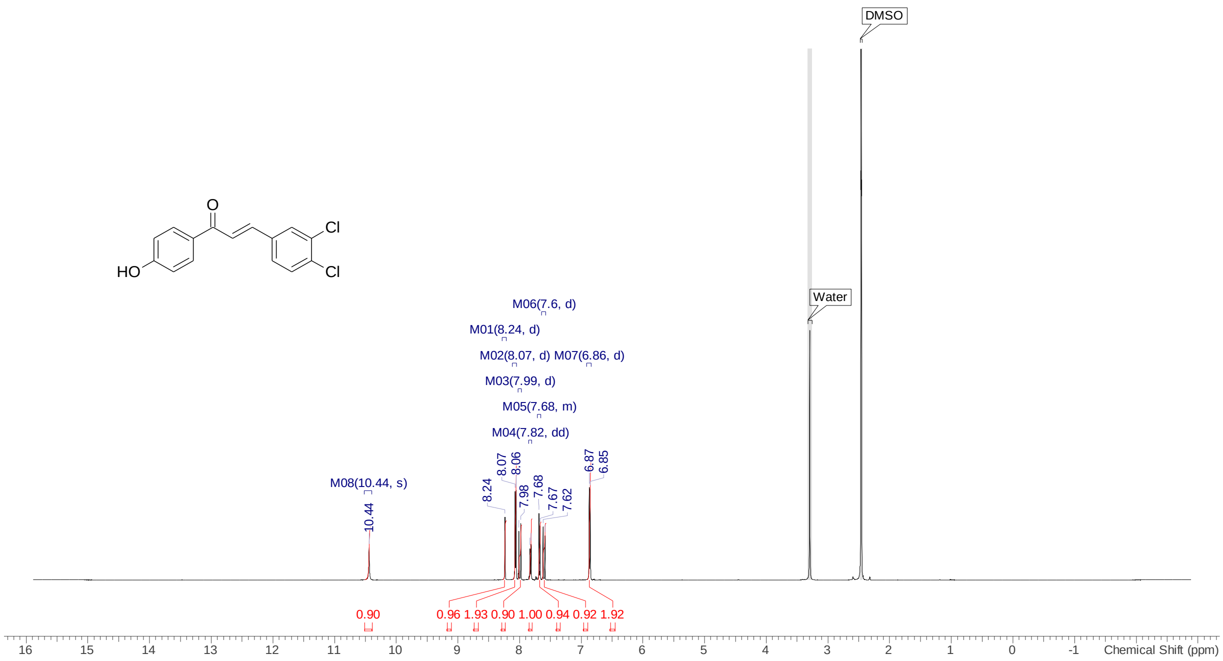

Figure S10. ^{1}H NMR spectrum of compound **3**.

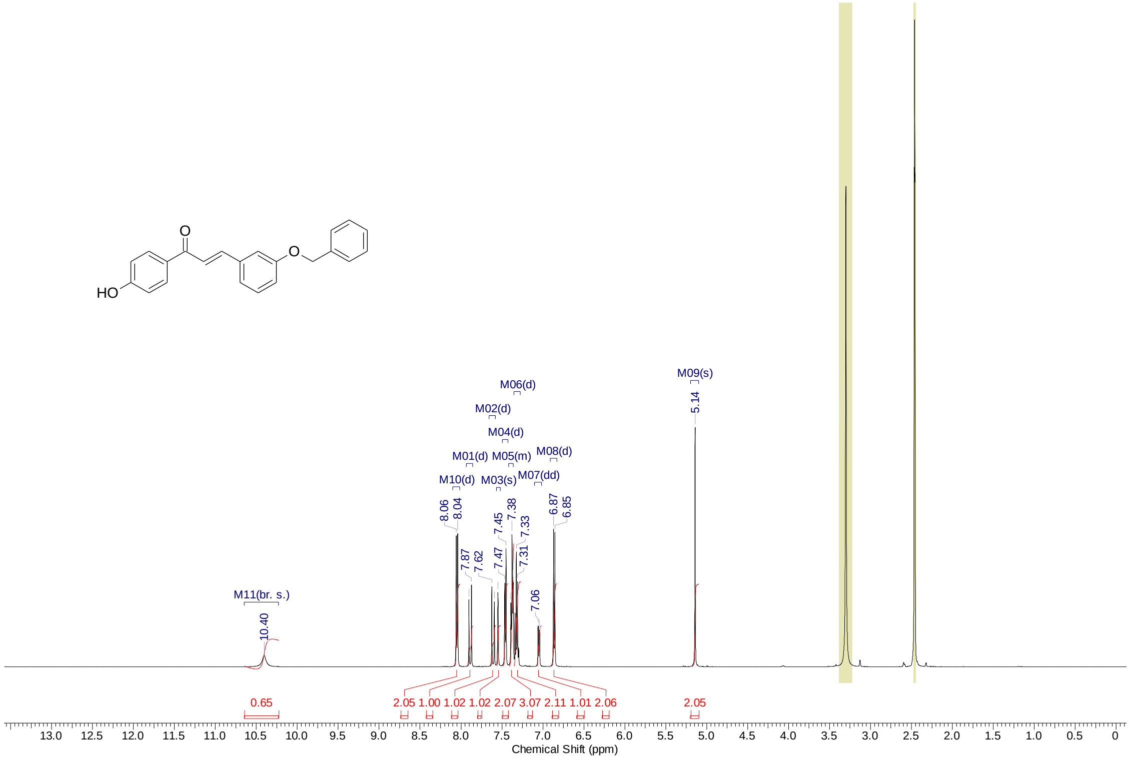

Figure S11. ^{1}H NMR spectrum of compound **4**.

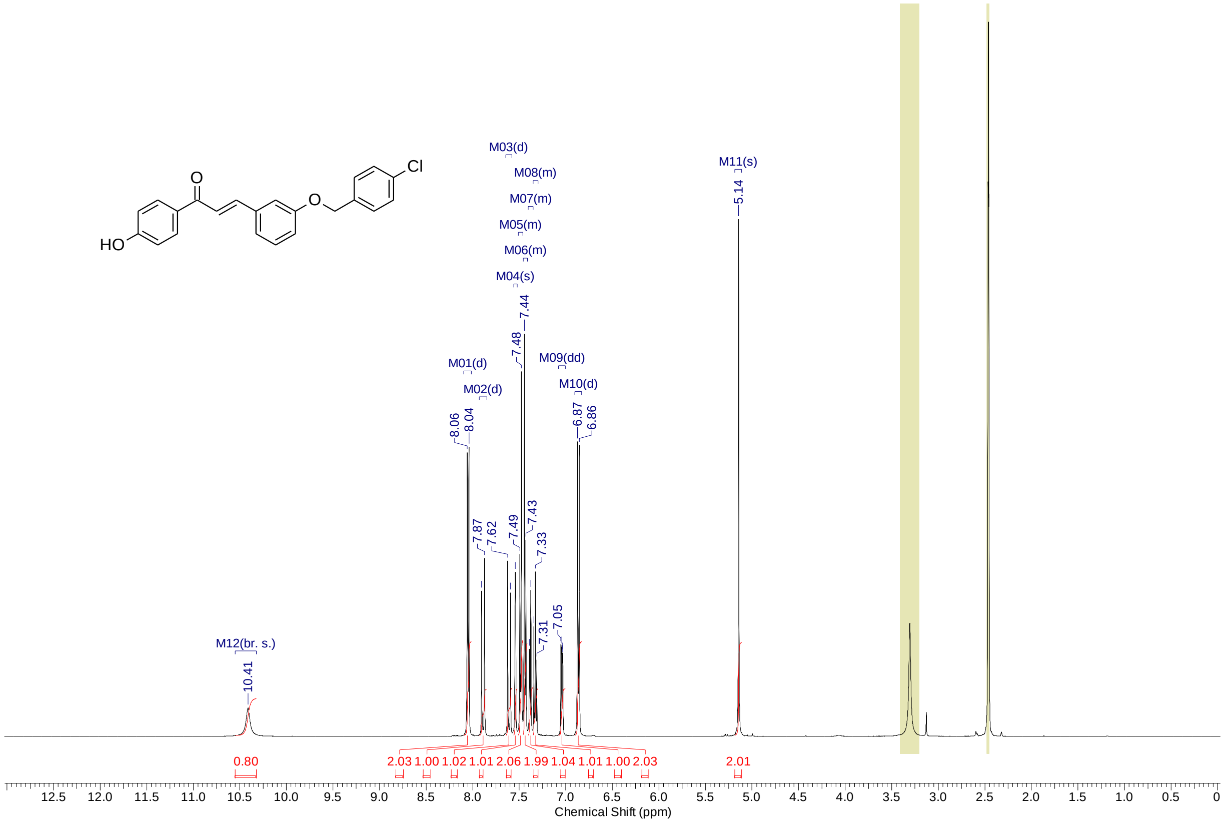

Figure S12. ^{1}H NMR spectrum of compound **5**.

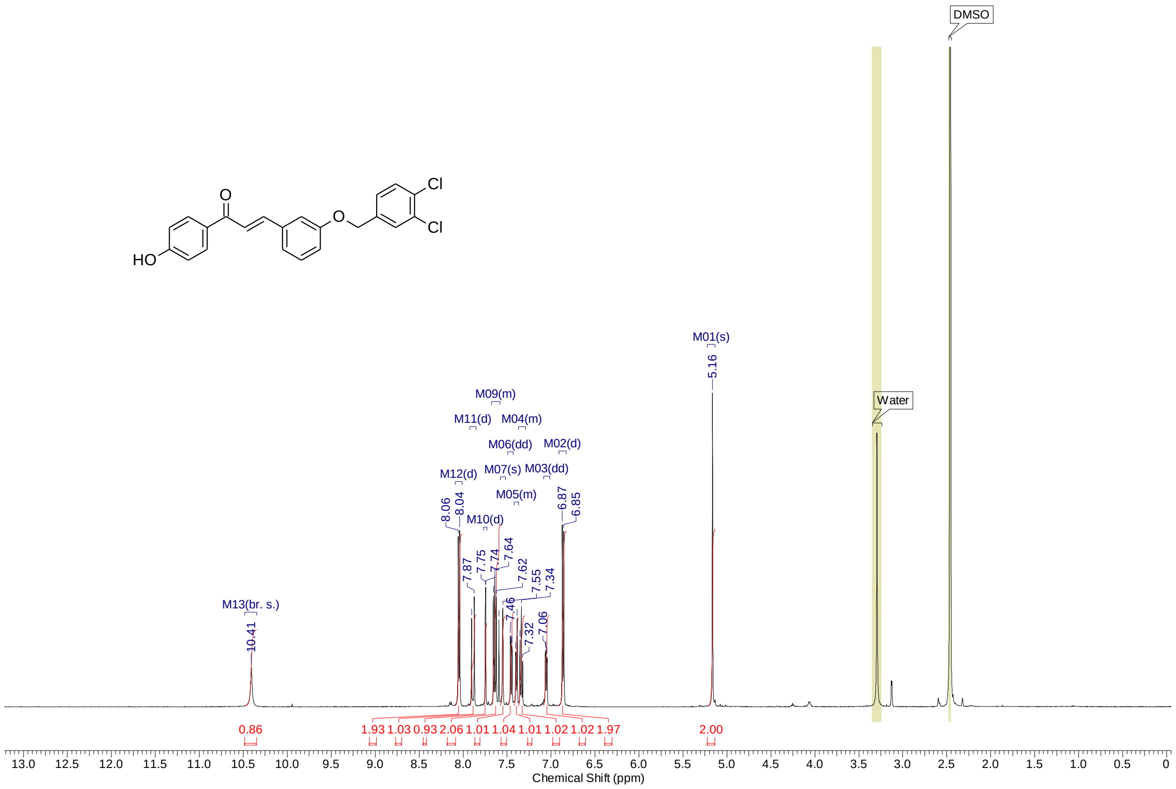

Figure S13. ^{1}H NMR spectrum of compound **6**.

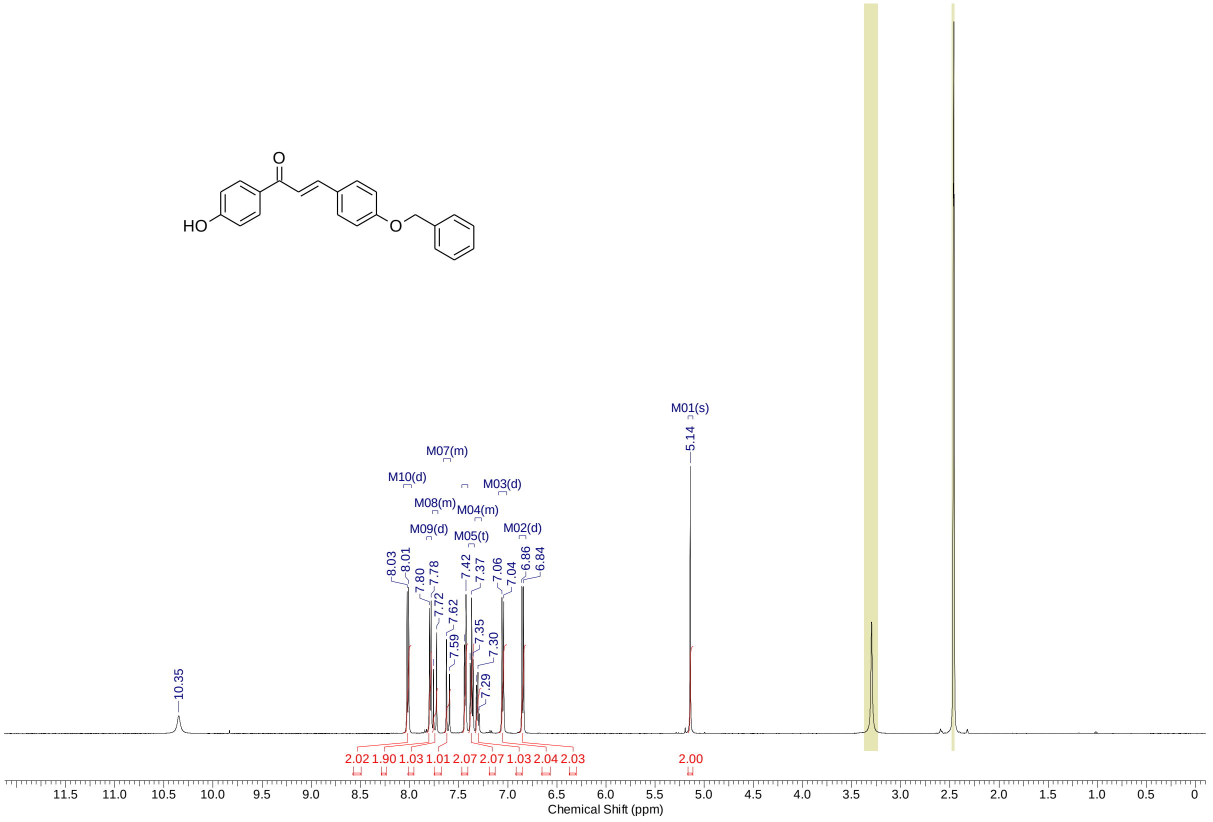

Figure S14. ^{1}H NMR spectrum of compound **7**.

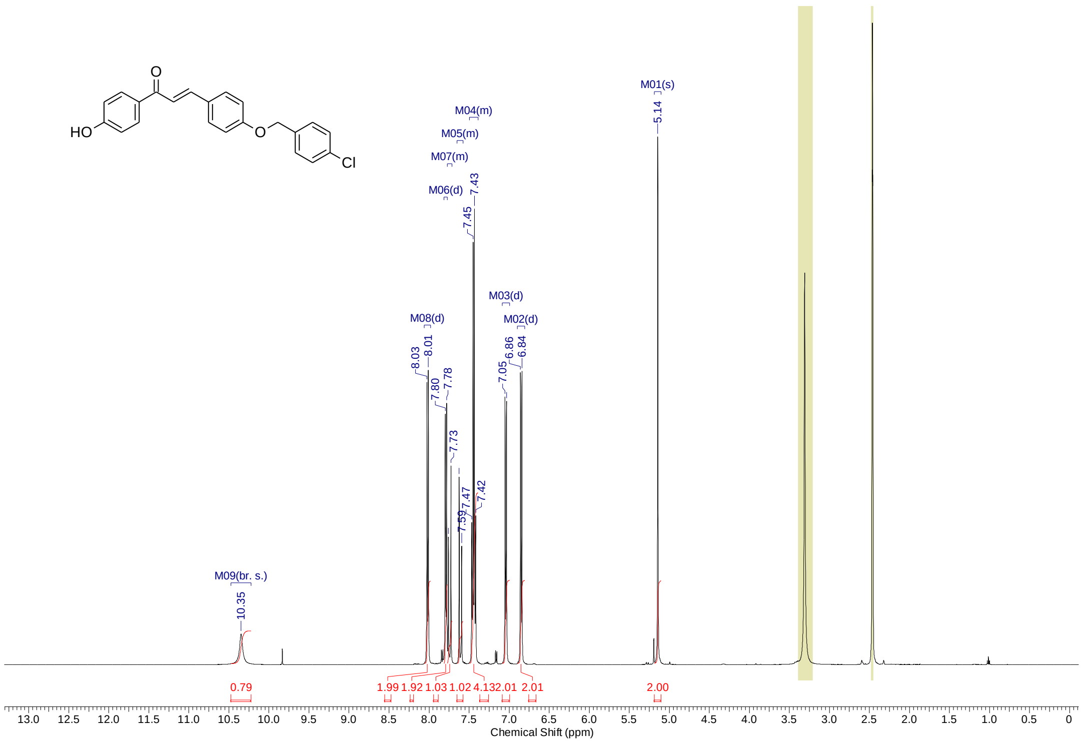

Figure S15. ^{1}H NMR spectrum of compound **8**.

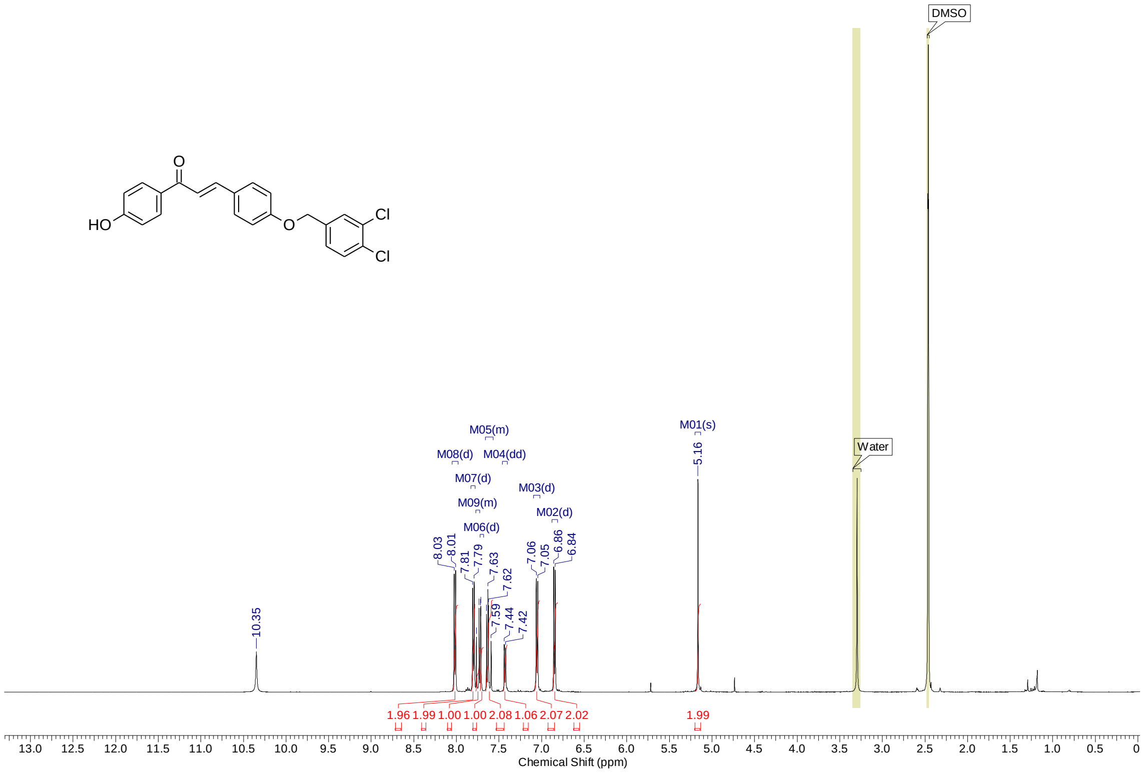

Figure S16. ^{1}H NMR spectrum of compound **9**.

^{1}H and ^{13}C NMR spectra of compounds 10-18

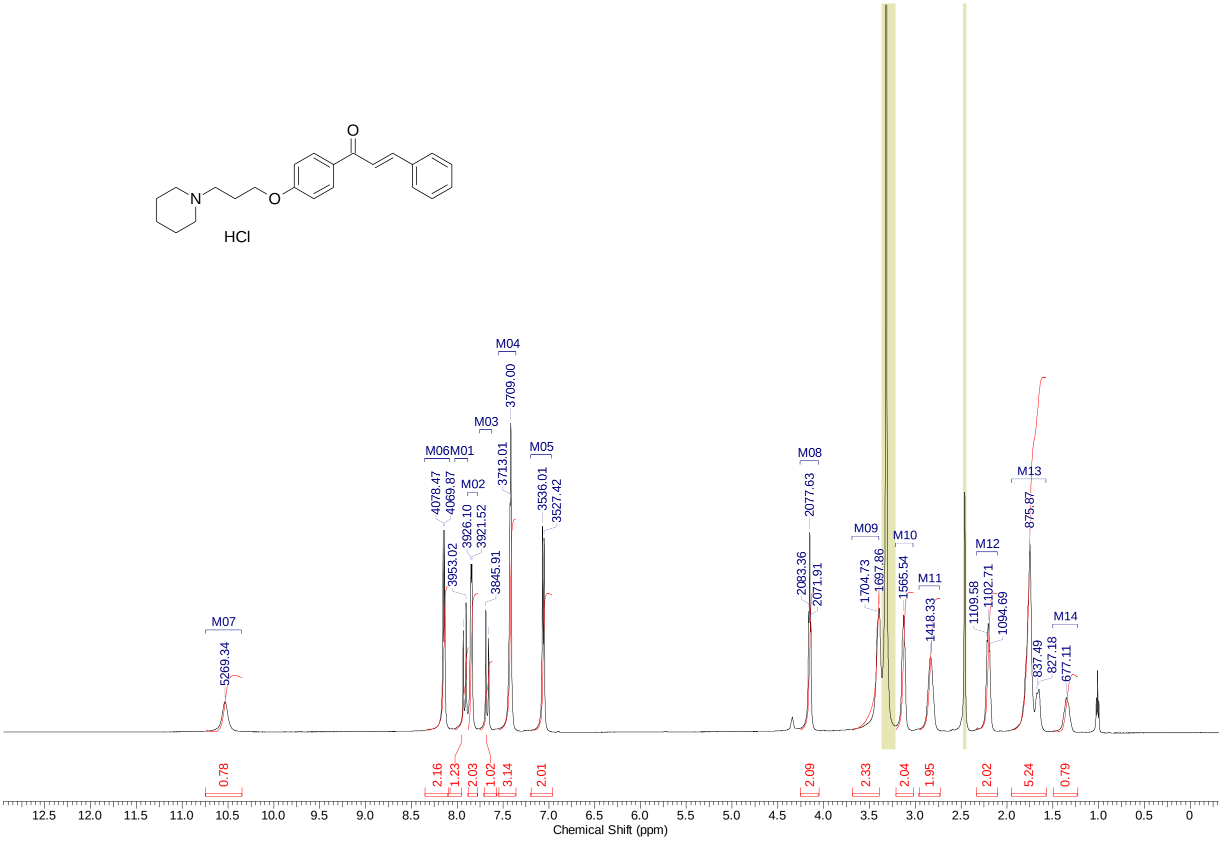

Figure S17. ^{1}H NMR spectrum of compound **10**.

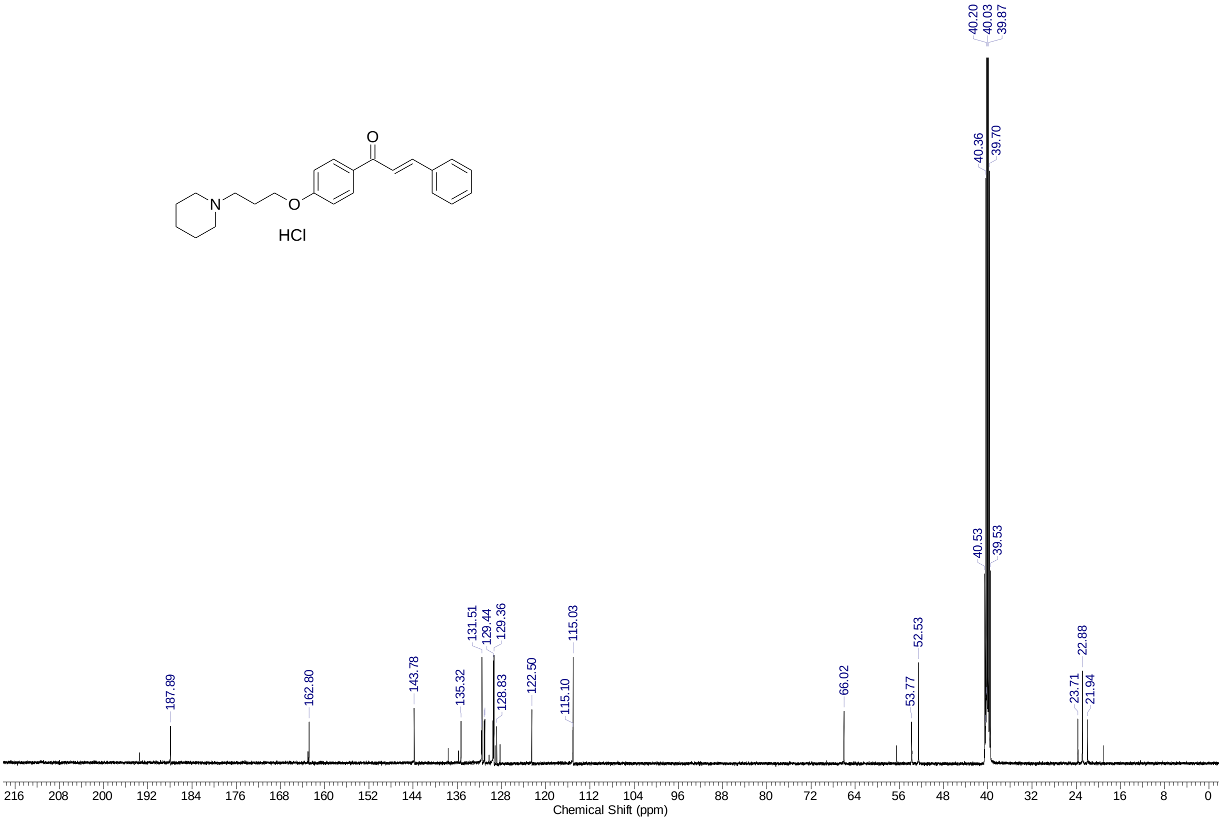

Figure S18. ^{13}C NMR spectrum of compound **10**.

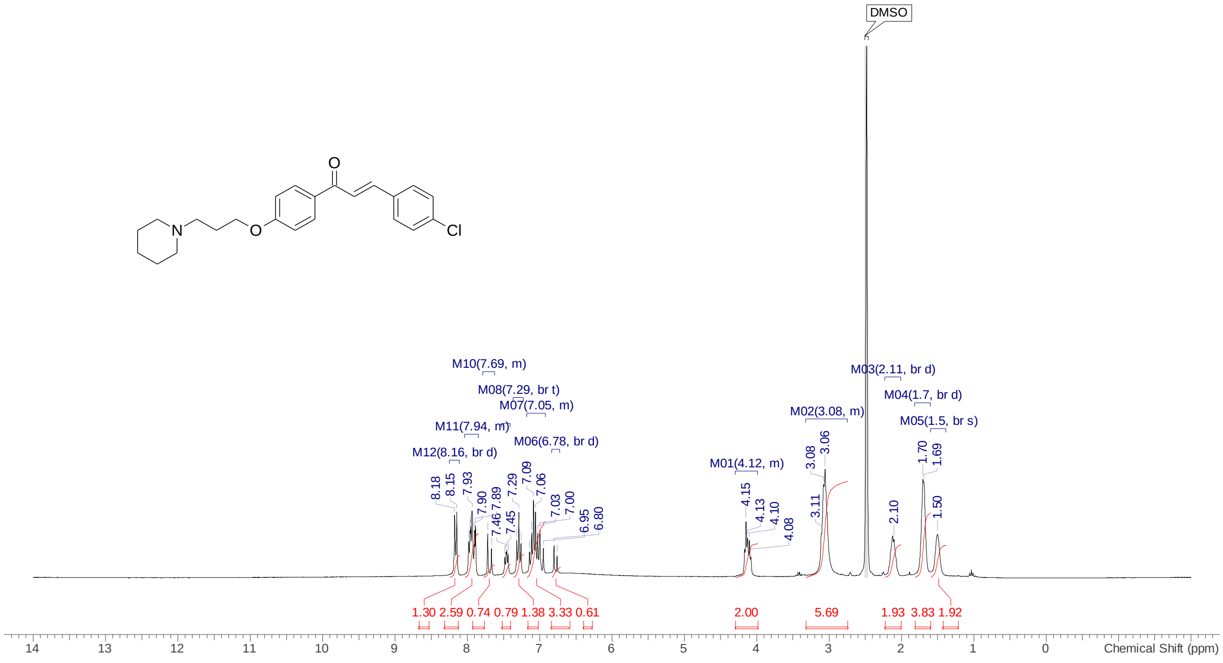

Figure S19. ^{1}H NMR spectrum of compound **11**.

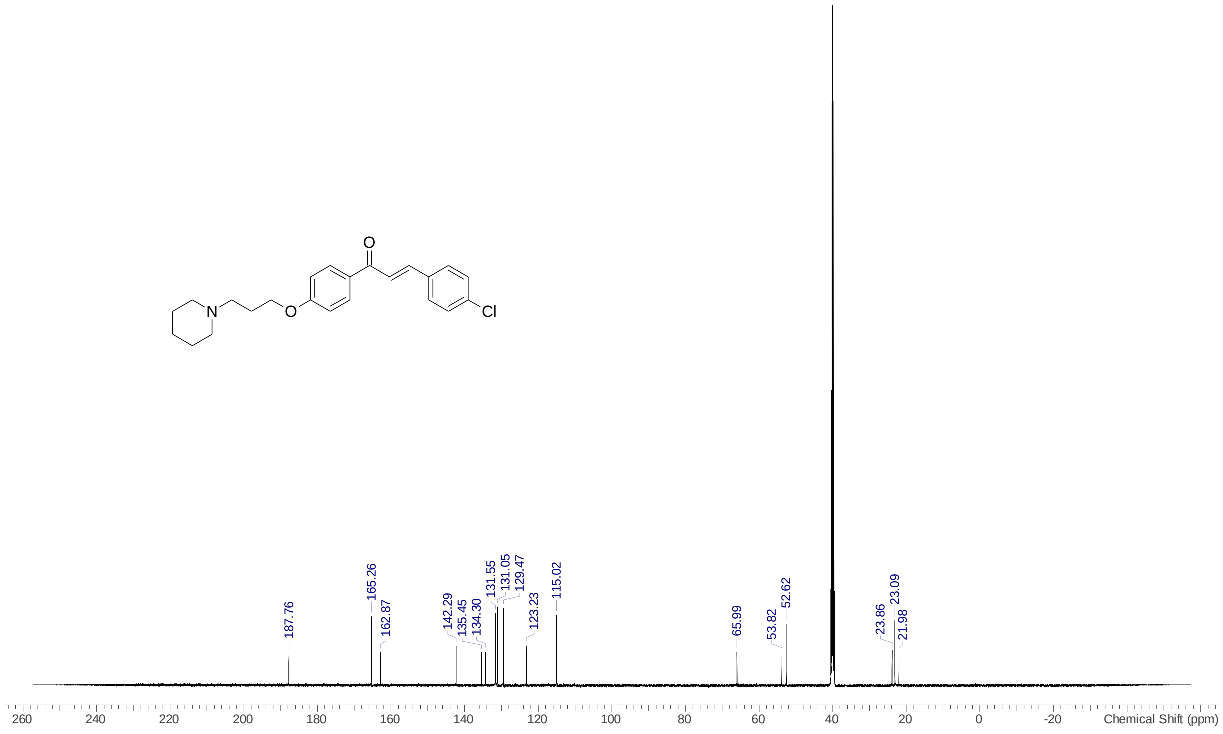

Figure S20. ^{13}C NMR spectrum of compound **11**.

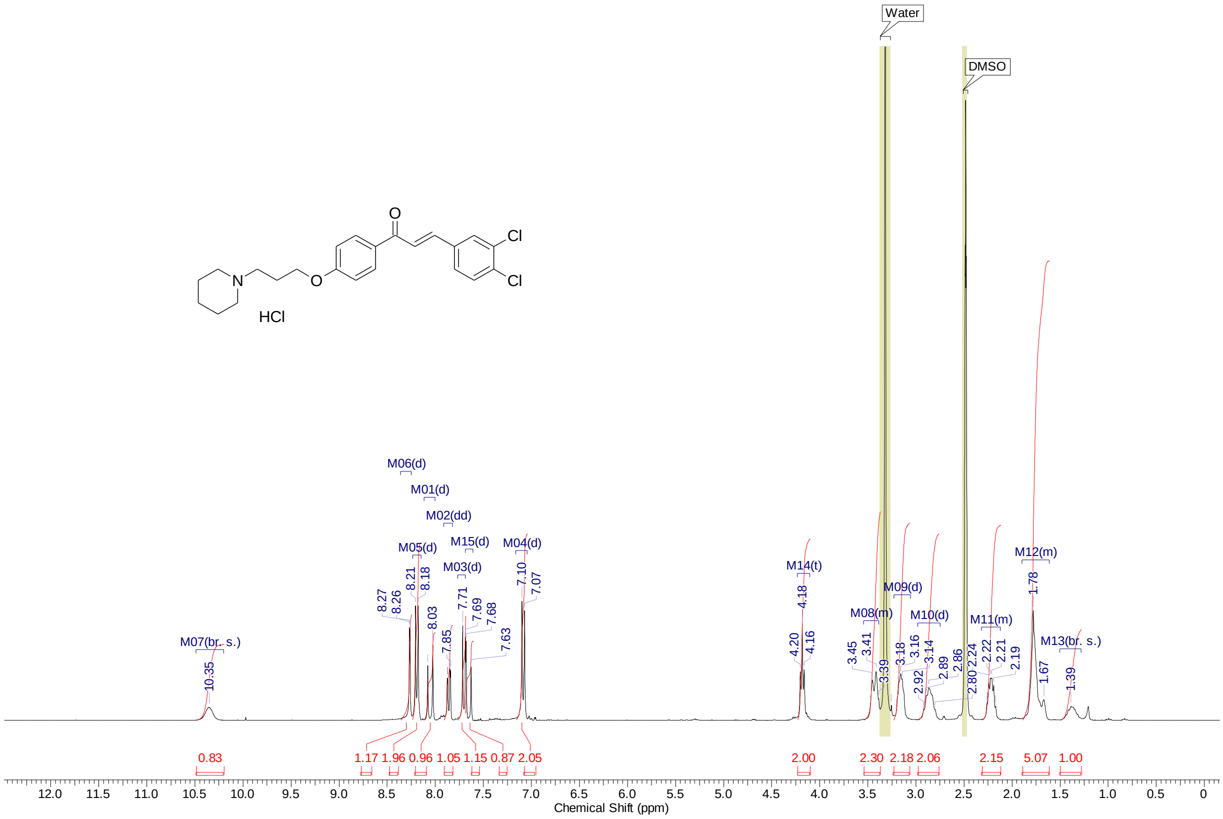

Figure S21. ^{1}H NMR spectrum of compound **12**.

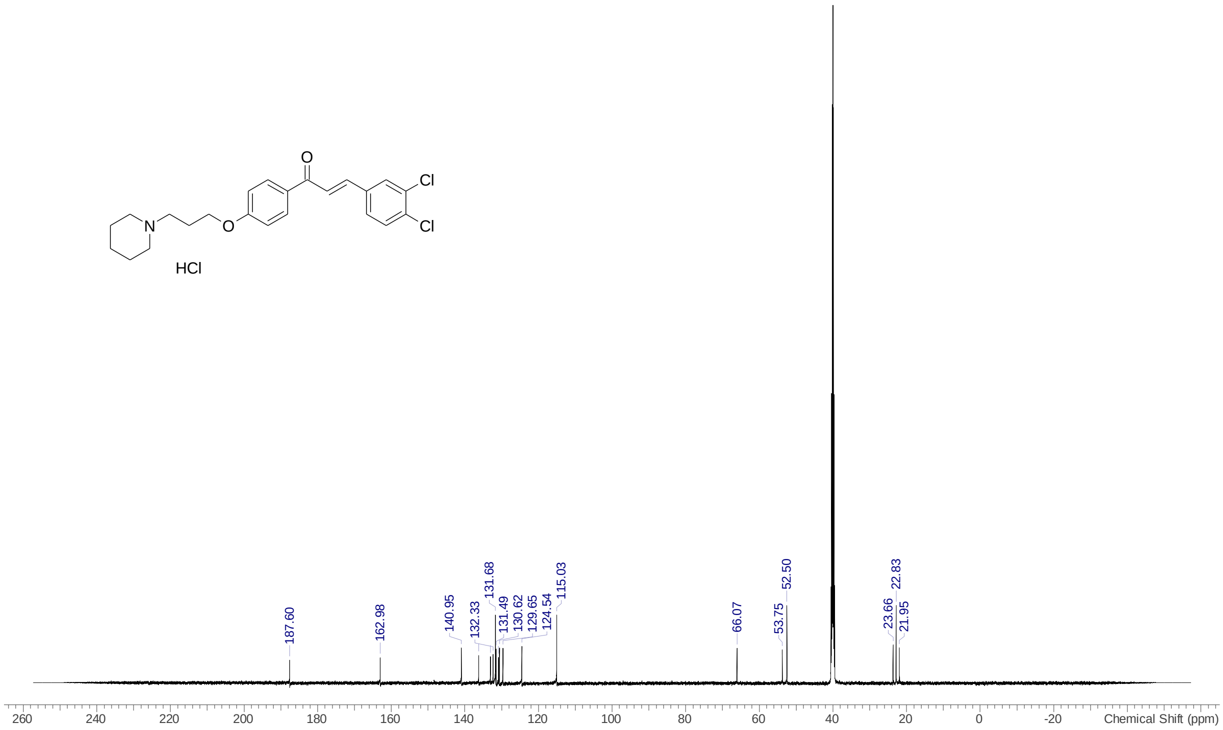

Figure S22. ^{13}C NMR spectrum of compound **12**.

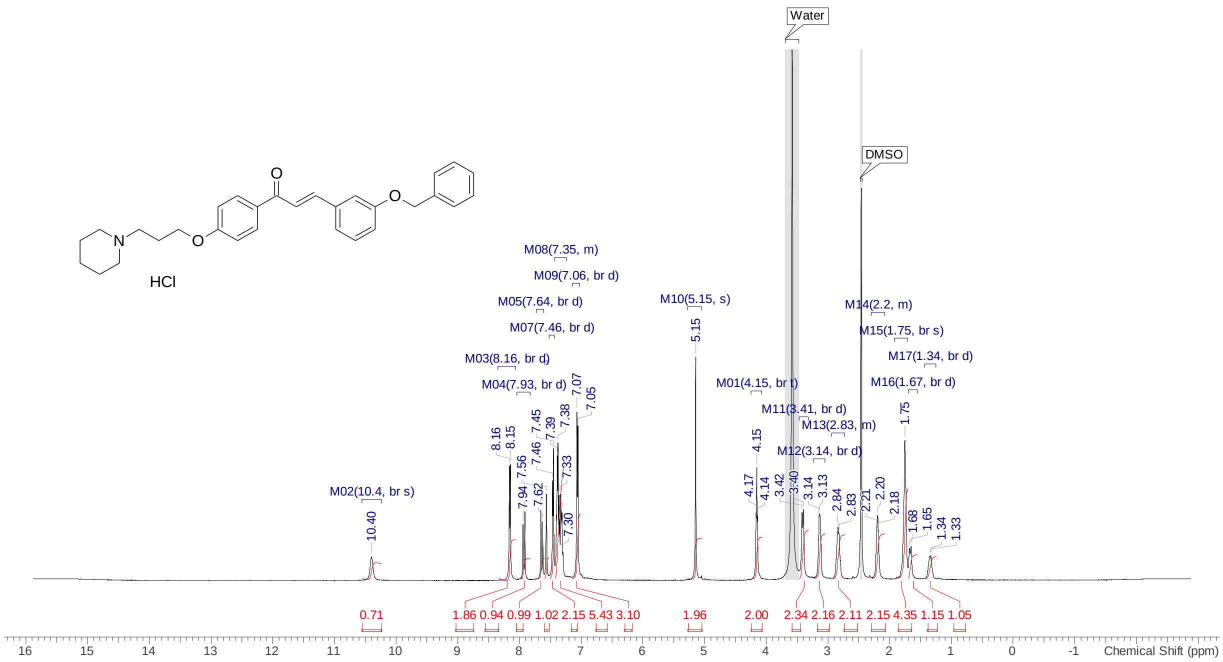

Figure S23. ^{1}H NMR spectrum of compound **13**.

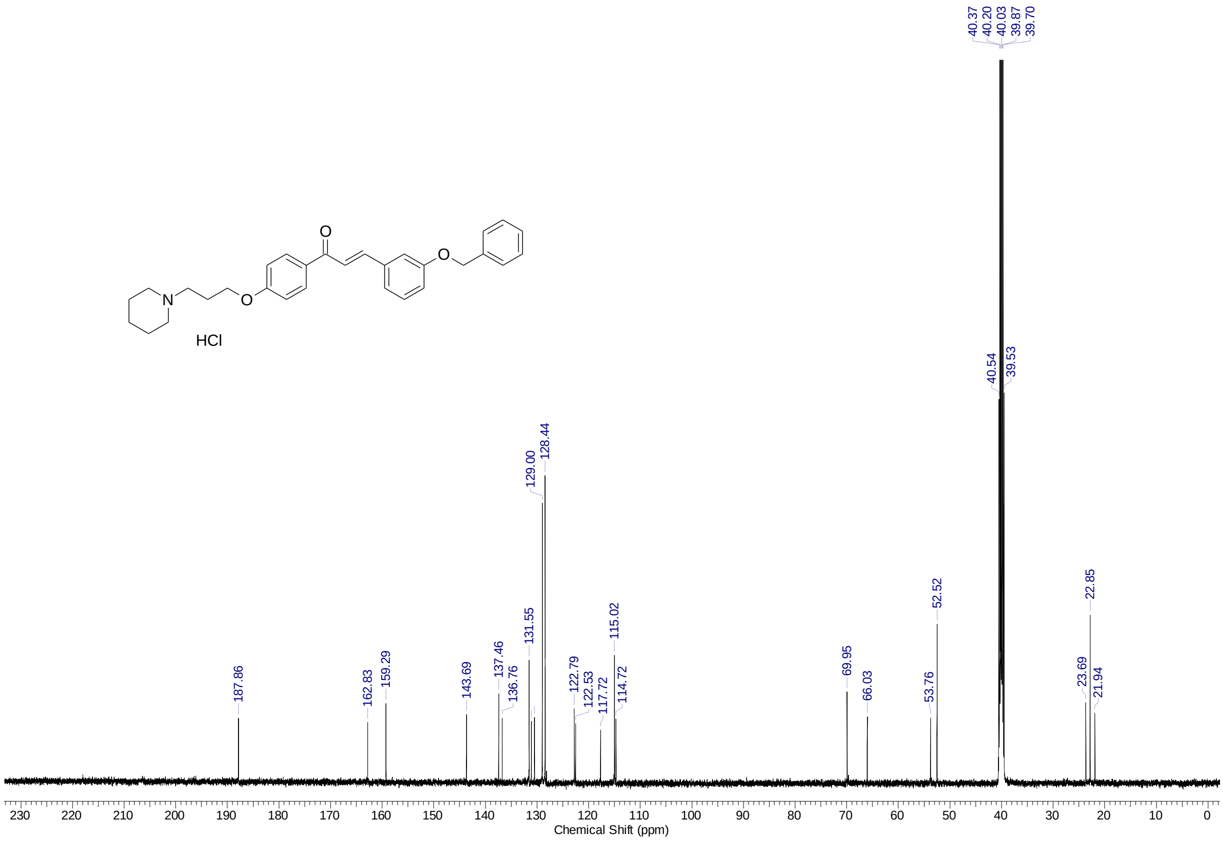

Figure S24. ^{13}C NMR spectrum of compound **13**.

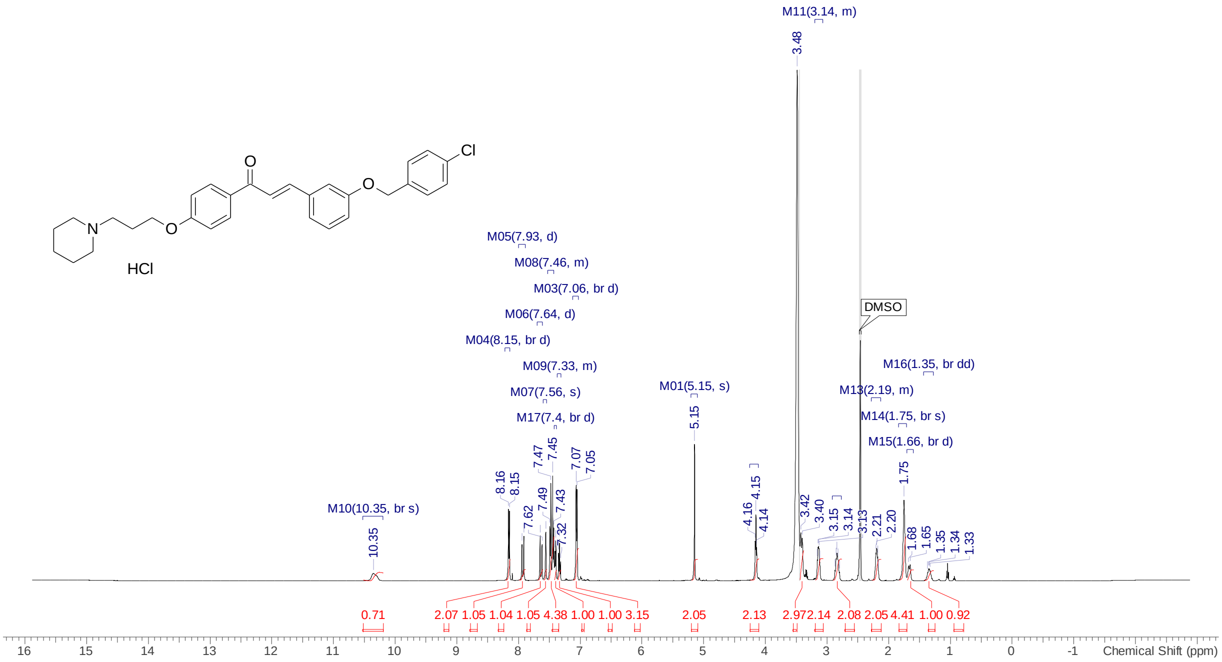

Figure S25. ^{1}H NMR spectrum of compound **14**.

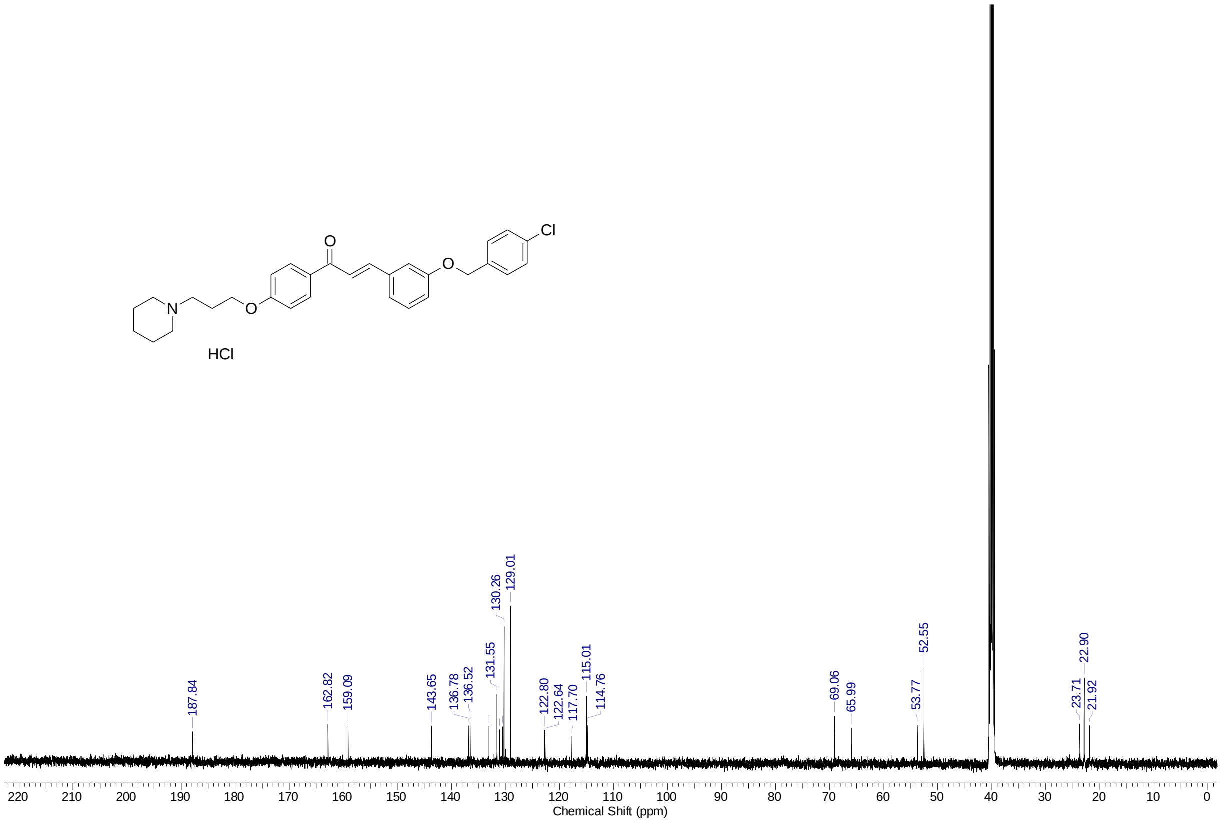

Figure S26. ^{13}C NMR spectrum of compound **14**.

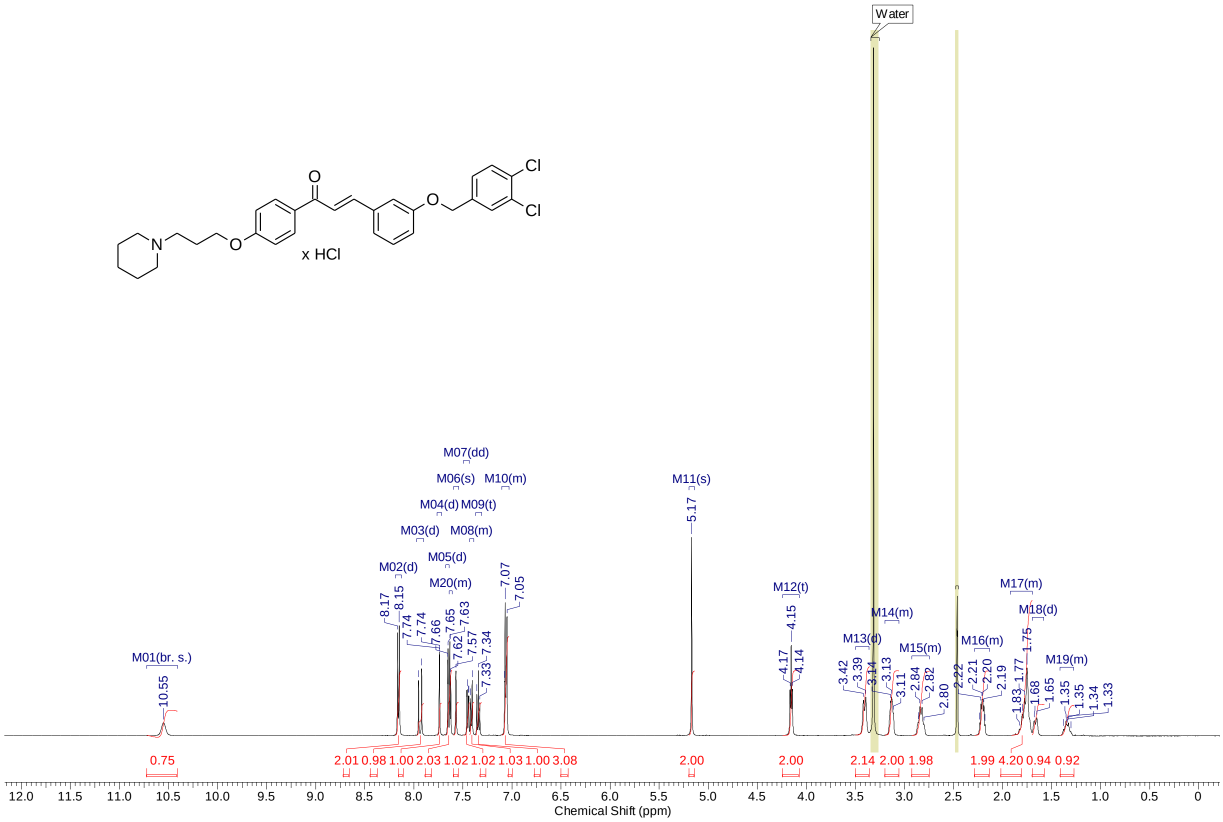

Figure S27. ^{1}H NMR spectrum of compound **15**.

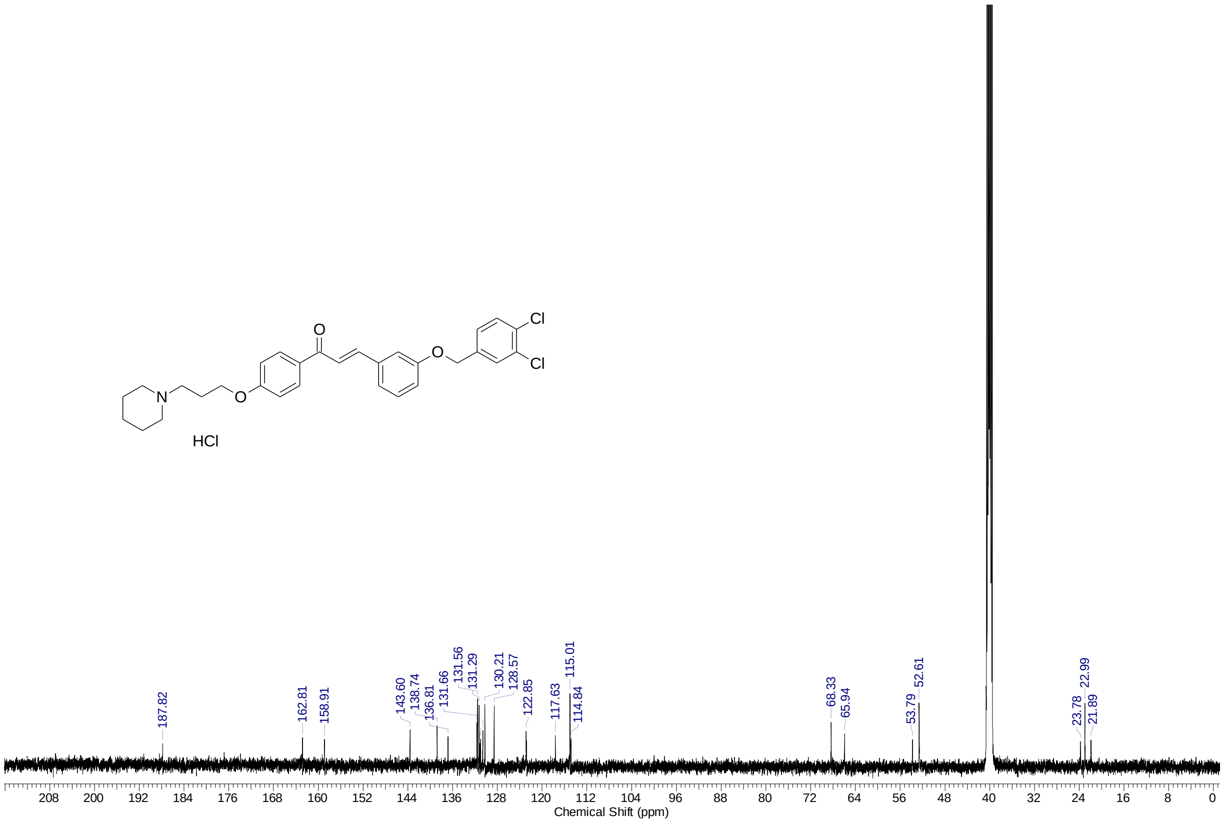

Figure S28. ^{13}C NMR spectrum of compound **15**.

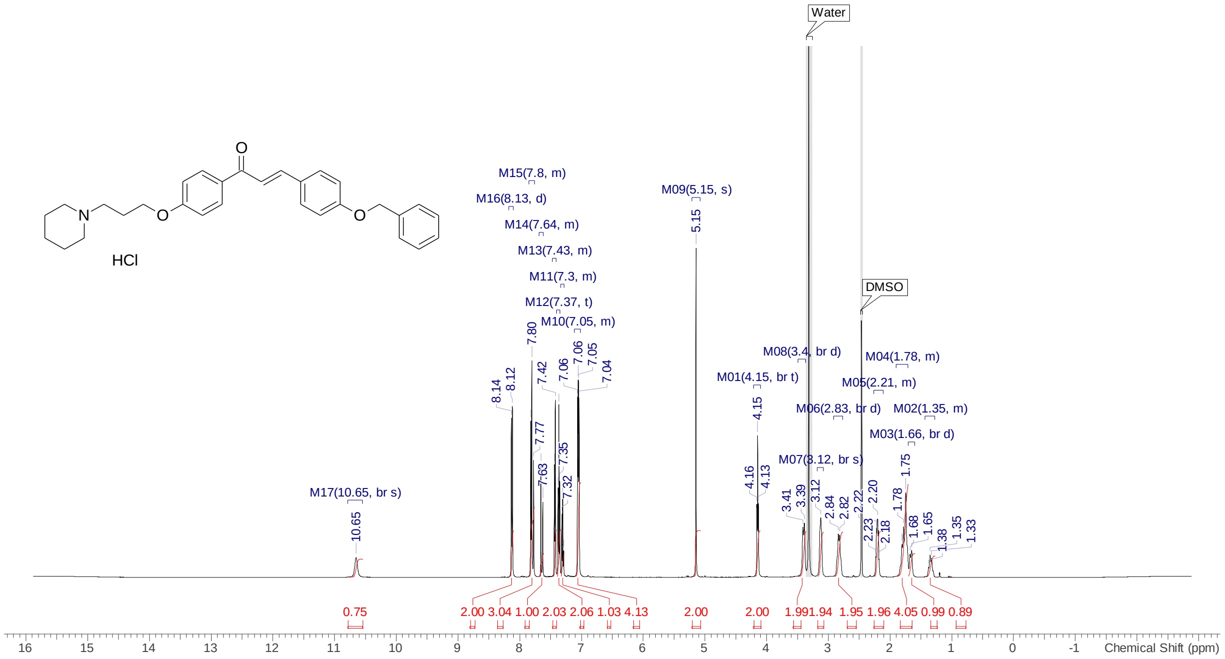

Figure S29. ^{1}H NMR spectrum of compound **16**.

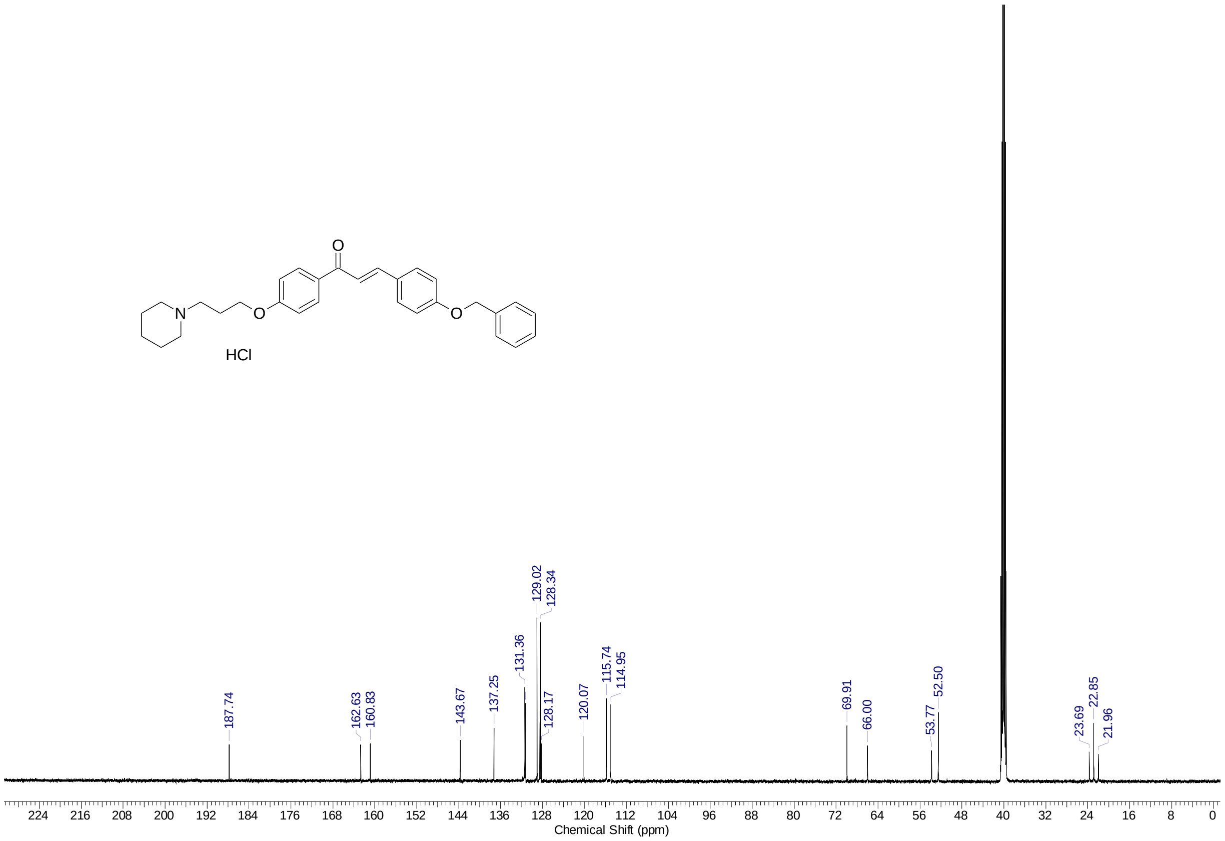

Figure S30. ^{13}C NMR spectrum of compound **16**.

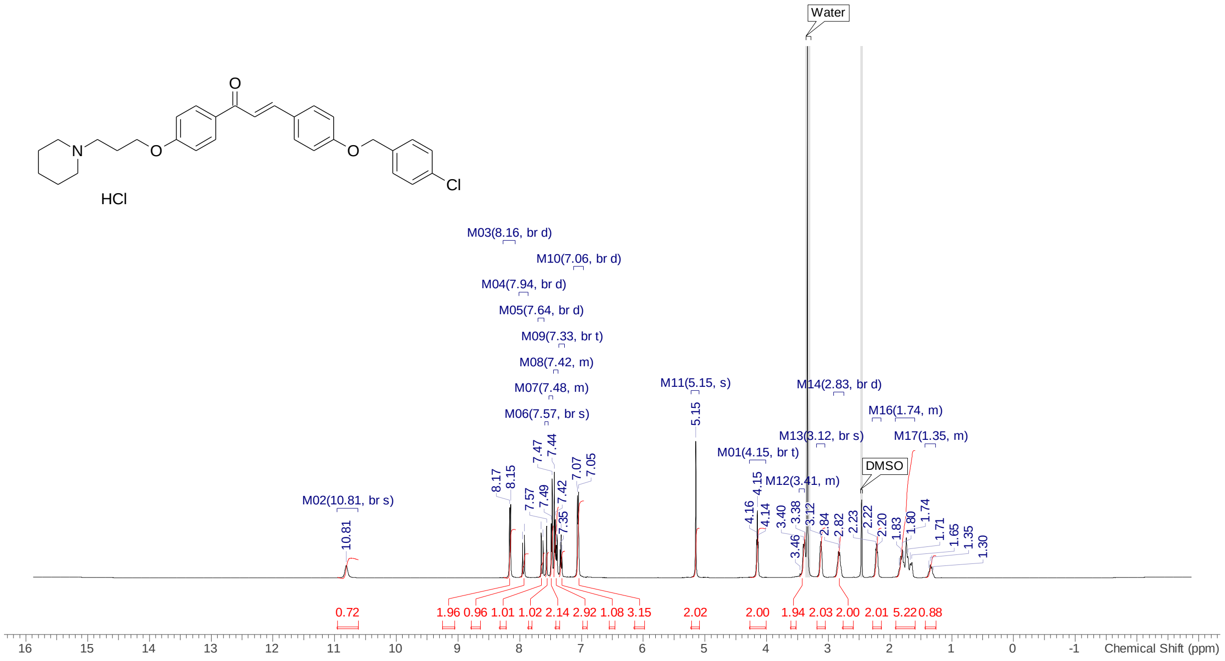

Figure S31. ^{1}H NMR spectrum of compound **17**.

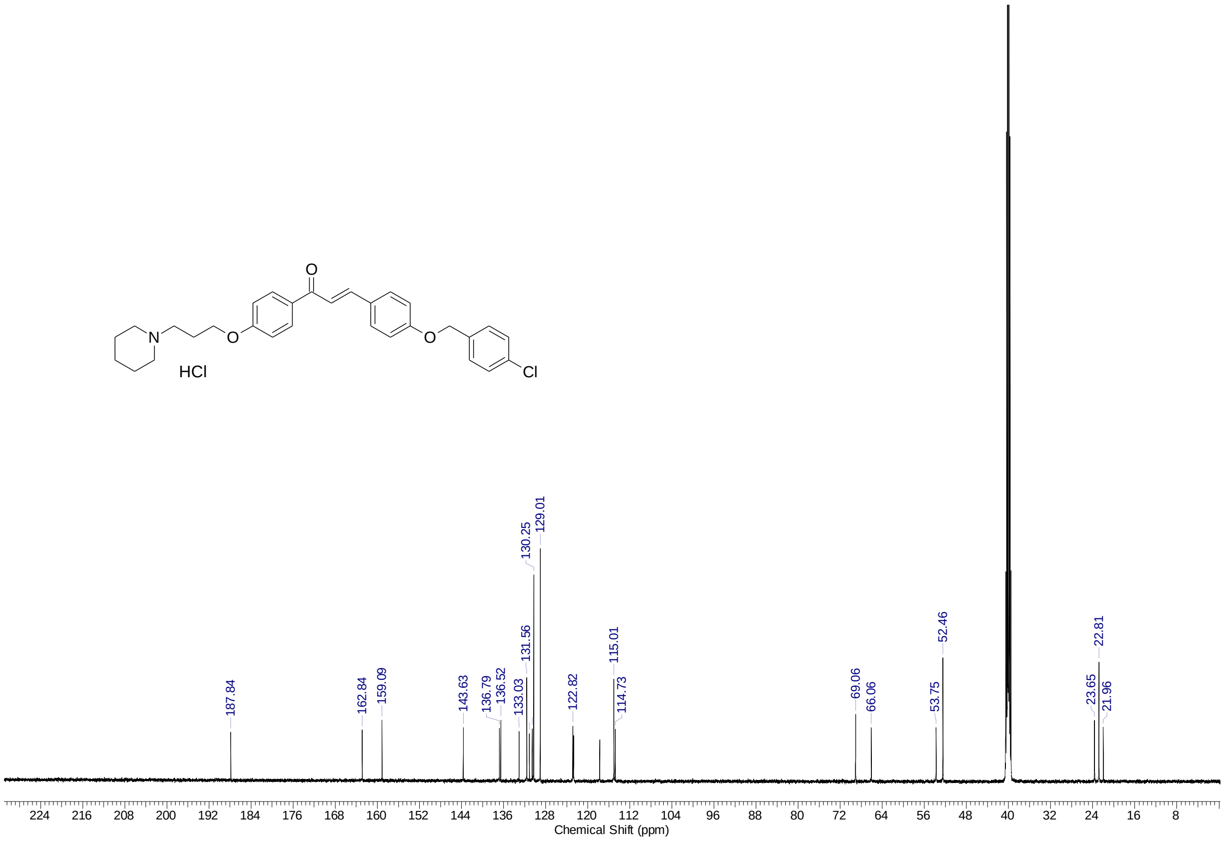

Figure S32. ^{13}C NMR spectrum of compound **17**.

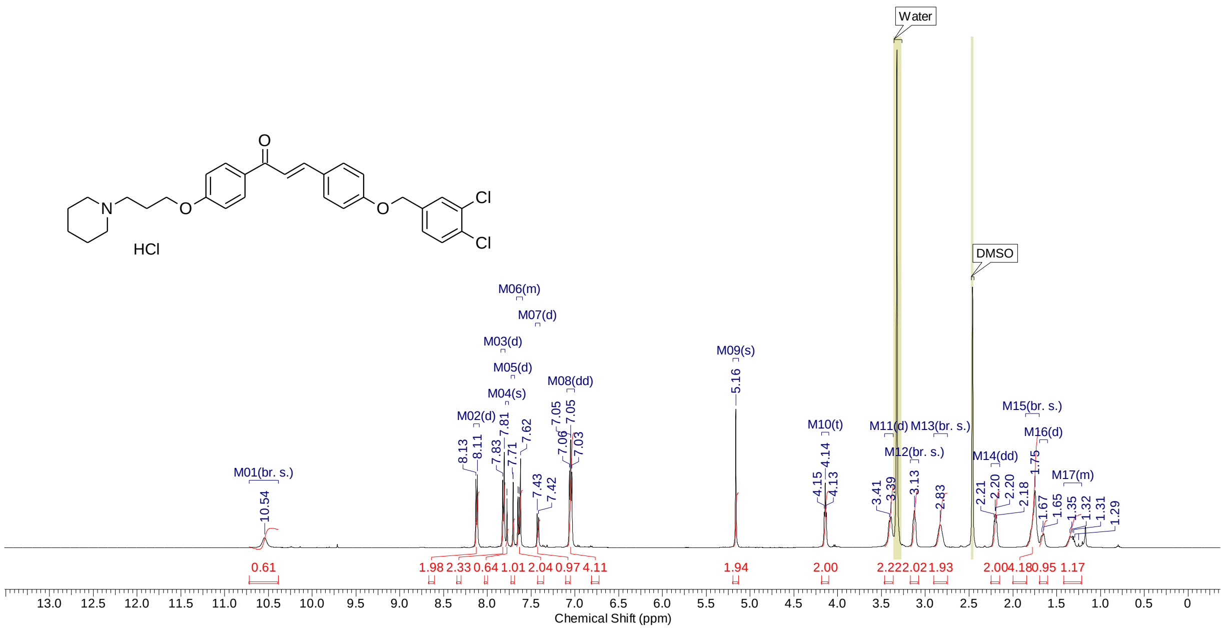

Figure S33. ^{1}H NMR spectrum of compound **18**.

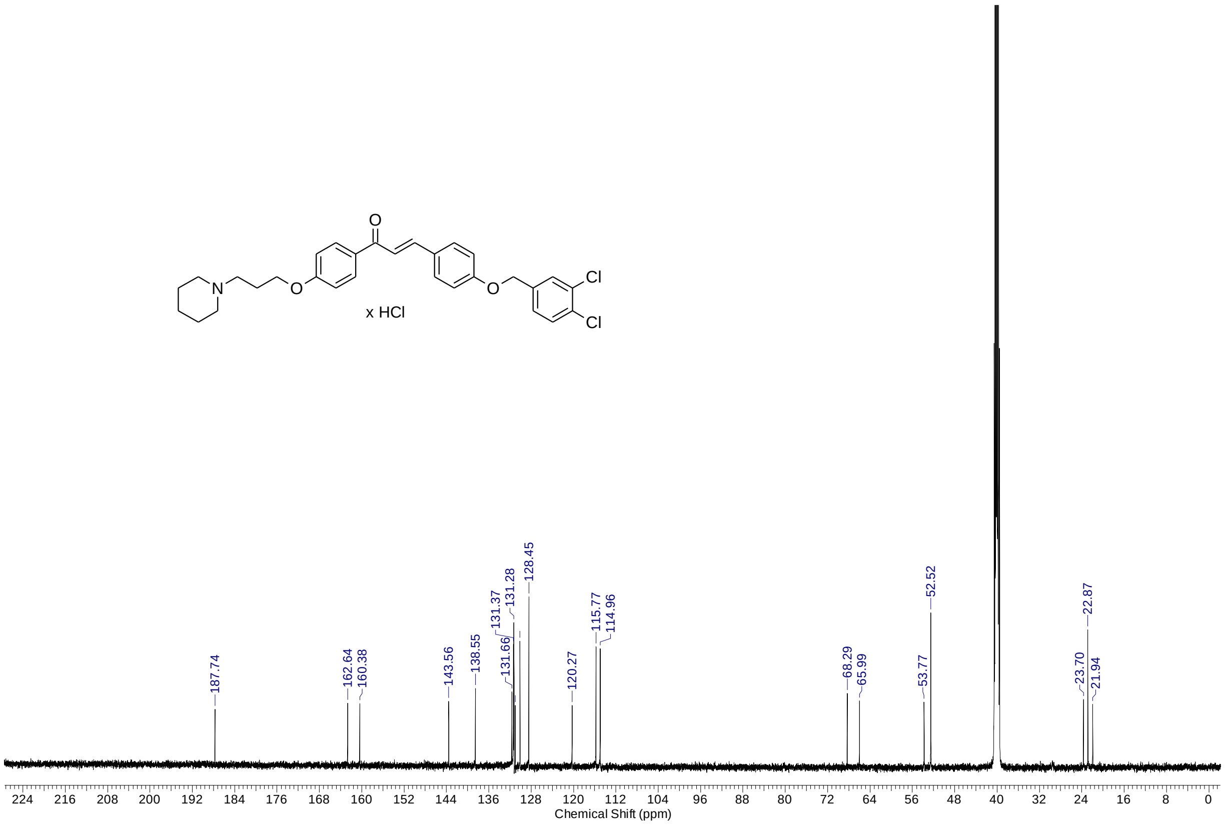

Figure S34. ^{13}C NMR spectrum of compound **18**.

Purity assessment of compounds 1-18 determined by LC-MS.

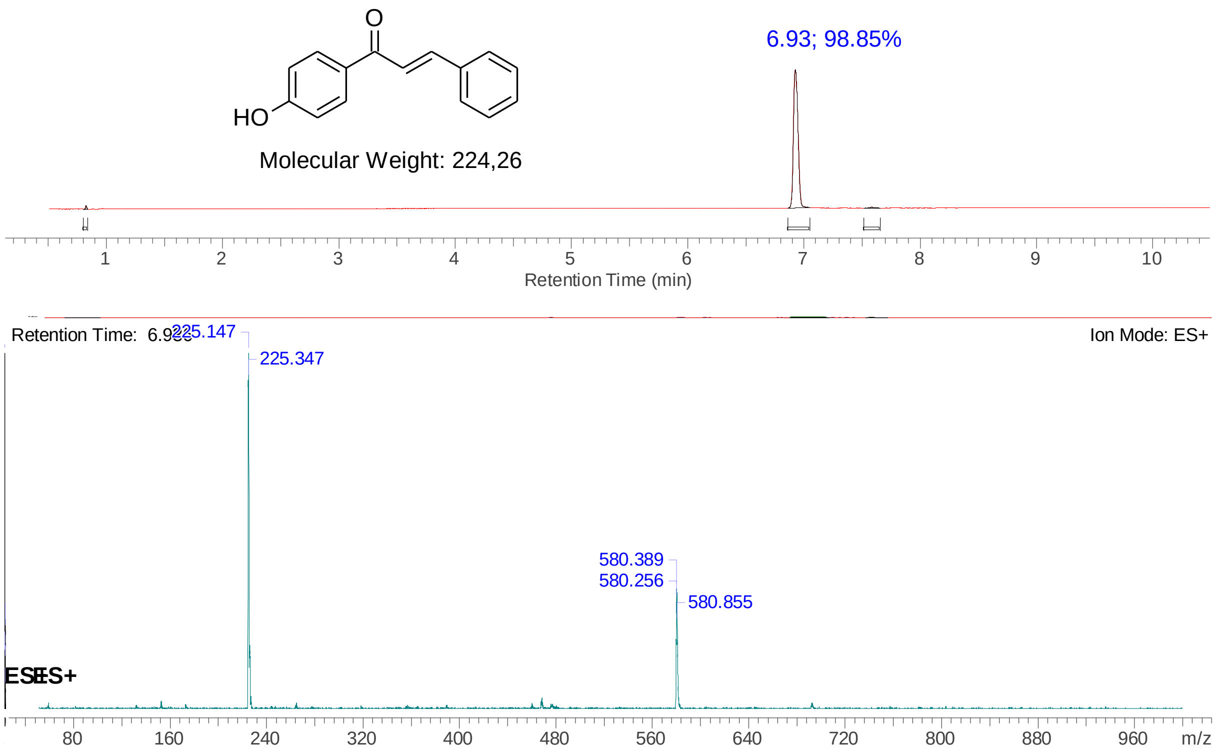

Figure S35. LC-MS assessment of the purity of compound **1**. The purity of compound **1** is 98.85% (retention time: 6.93 min).

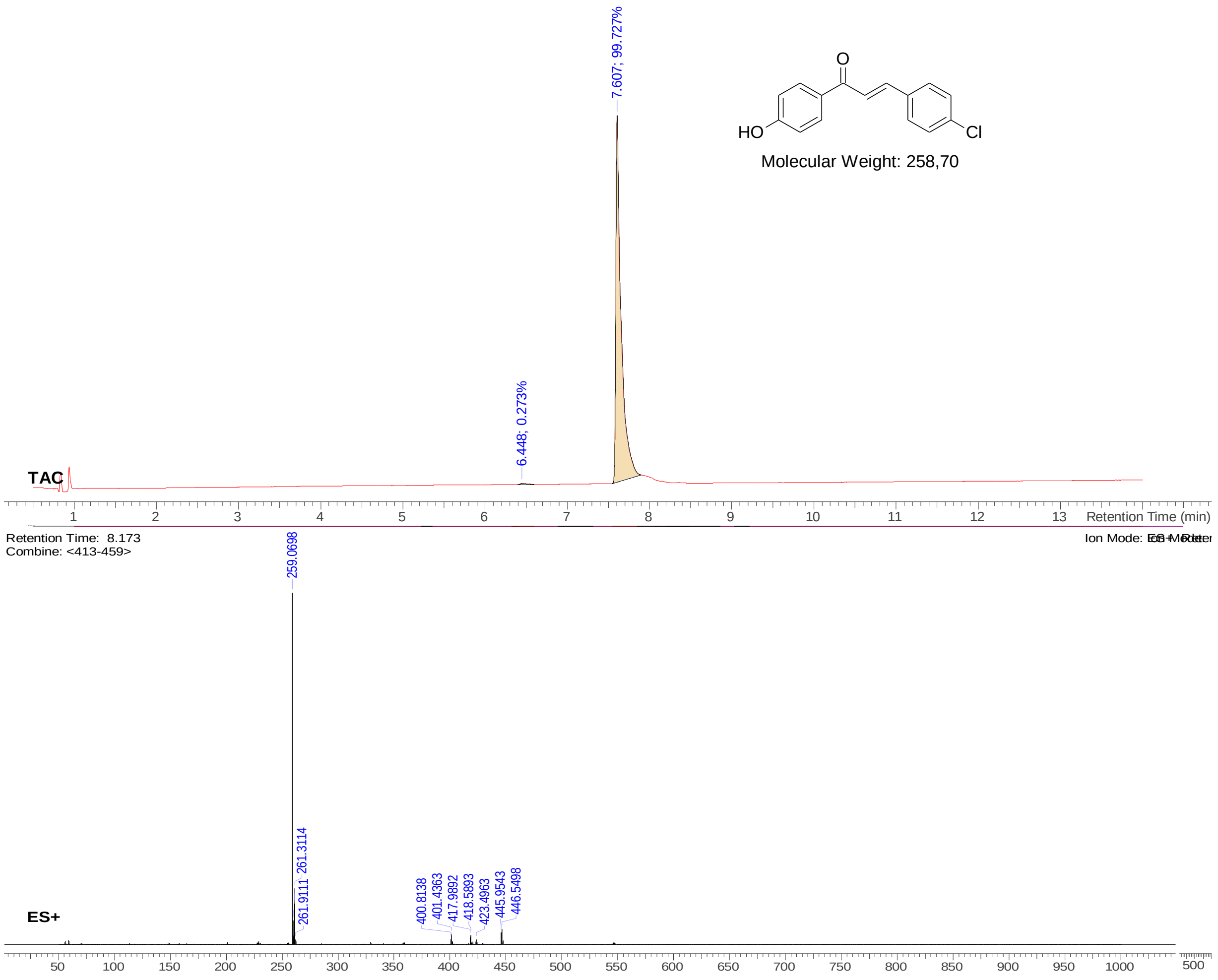

Figure S36. LC-MS assessment of the purity of compound **2**. The purity of compound **2** is 99.72% (retention time: 7.61 min).

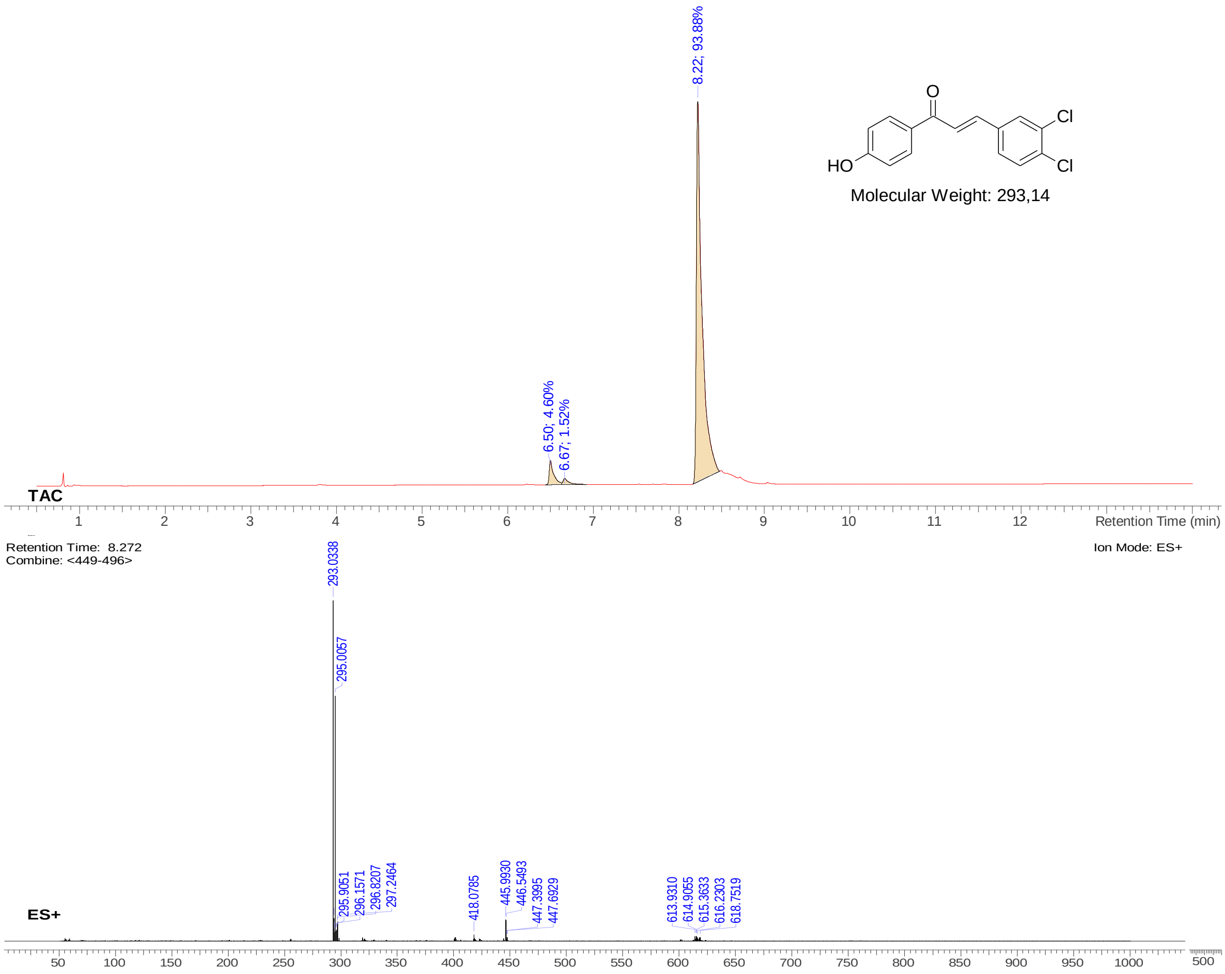

Figure S37. LC-MS assessment of the purity of compound **3**. The purity of compound **3** is 93.88% (retention time: 8.22 min).

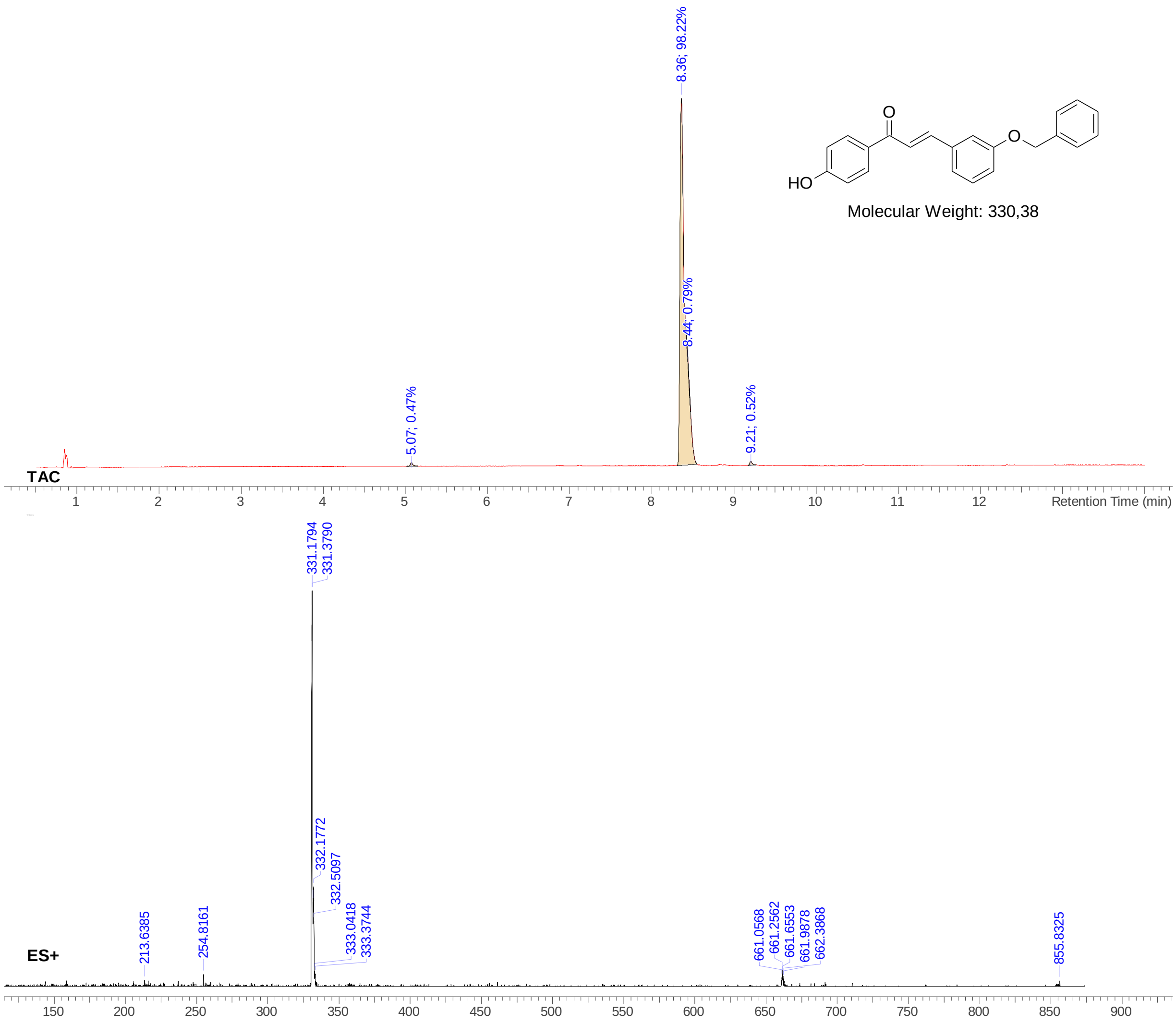

Figure S38. LC-MS assessment of the purity of compound **4**. The purity of compound **4** is 98.22% (retention time: 8.36 min).

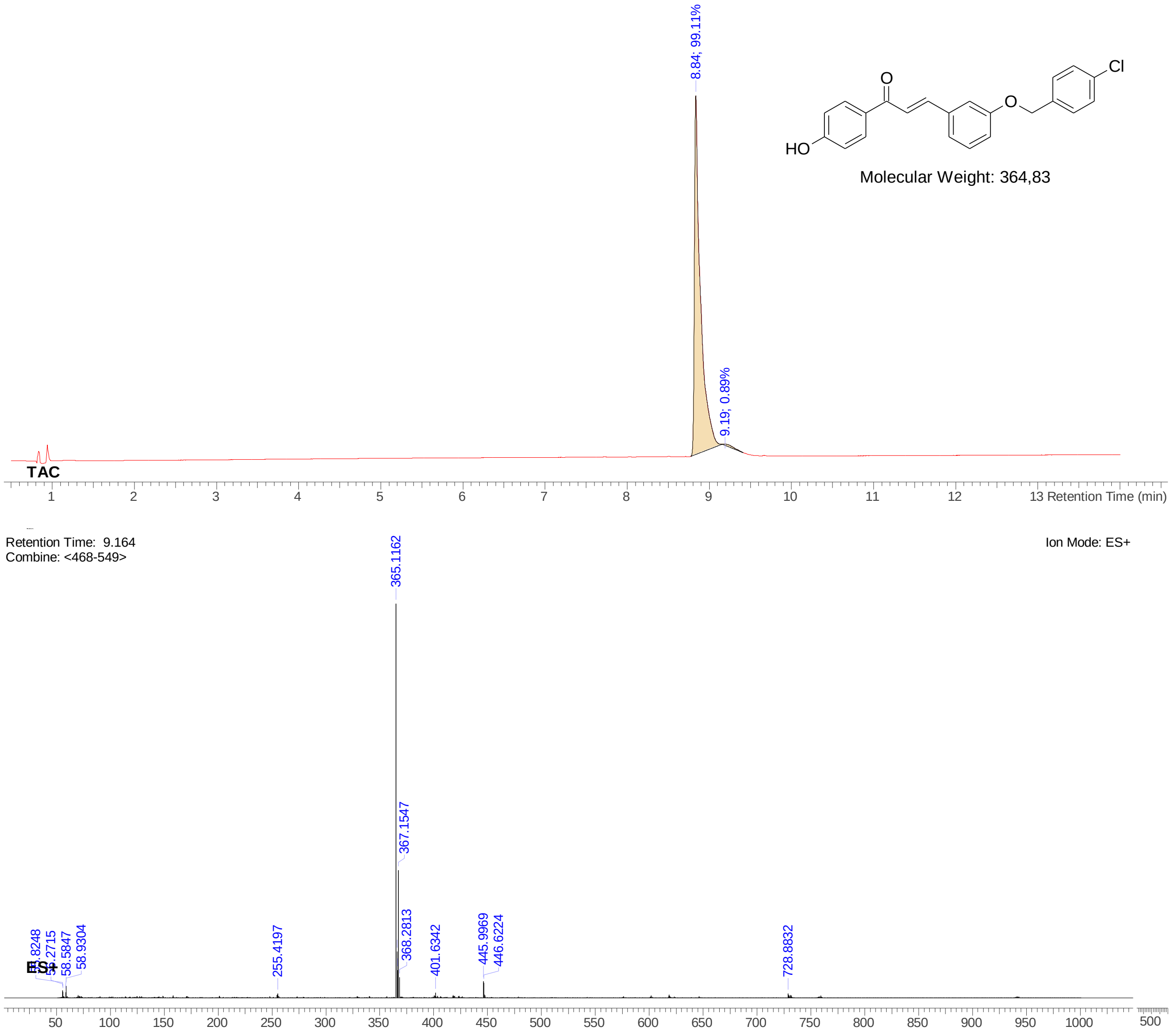

Figure S39. LC-MS assessment of the purity of compound **5**. The purity of compound **5** is 99.11% (retention time: 8.84 min).

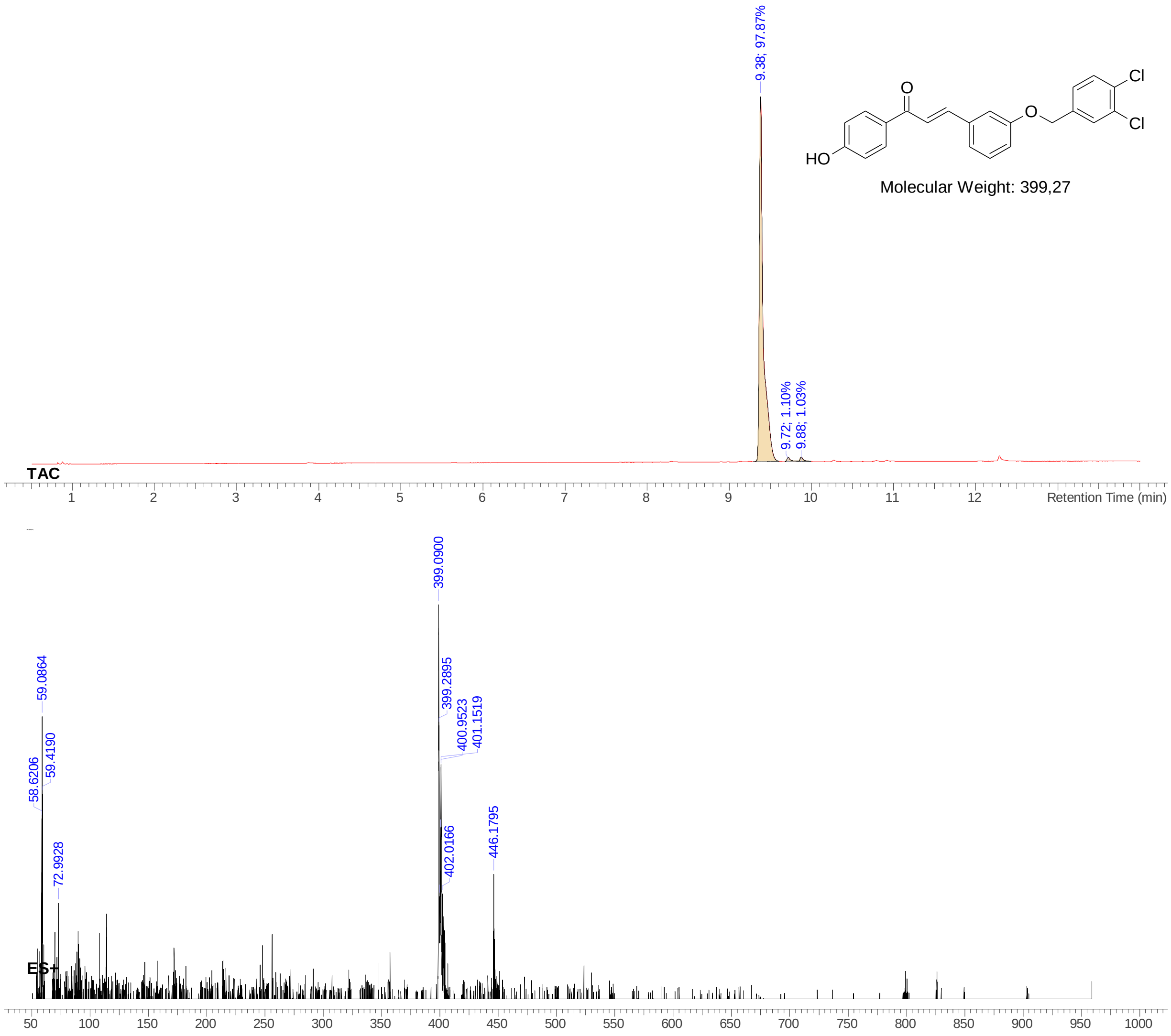

Figure S40. LC-MS assessment of the purity of compound **6**. The purity of compound **6** is 97.98% (retention time:9.38 min).

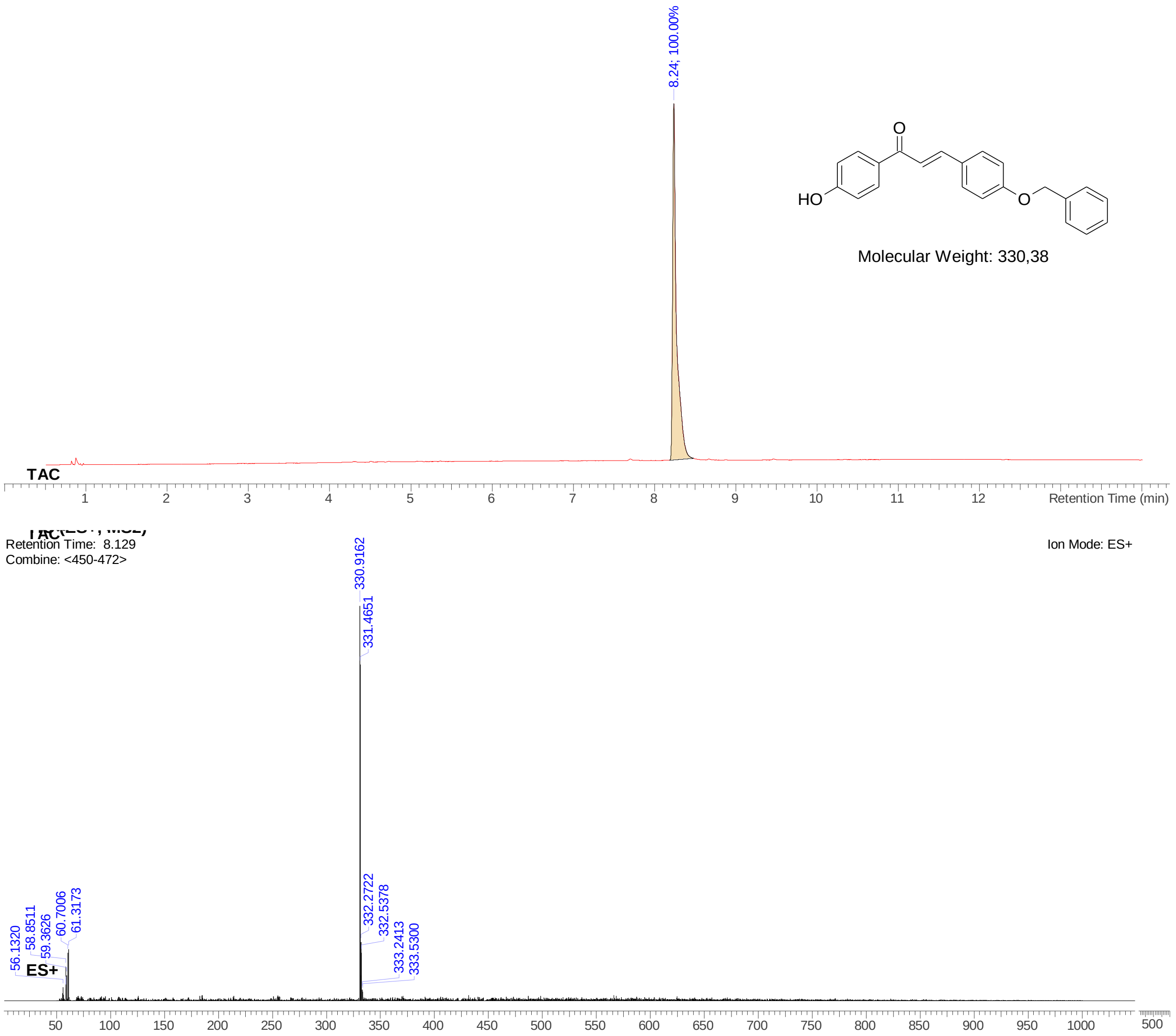

Figure S41. LC-MS assessment of the purity of compound **7**. The purity of compound **7** is 100% (retention time: 8.24 min).

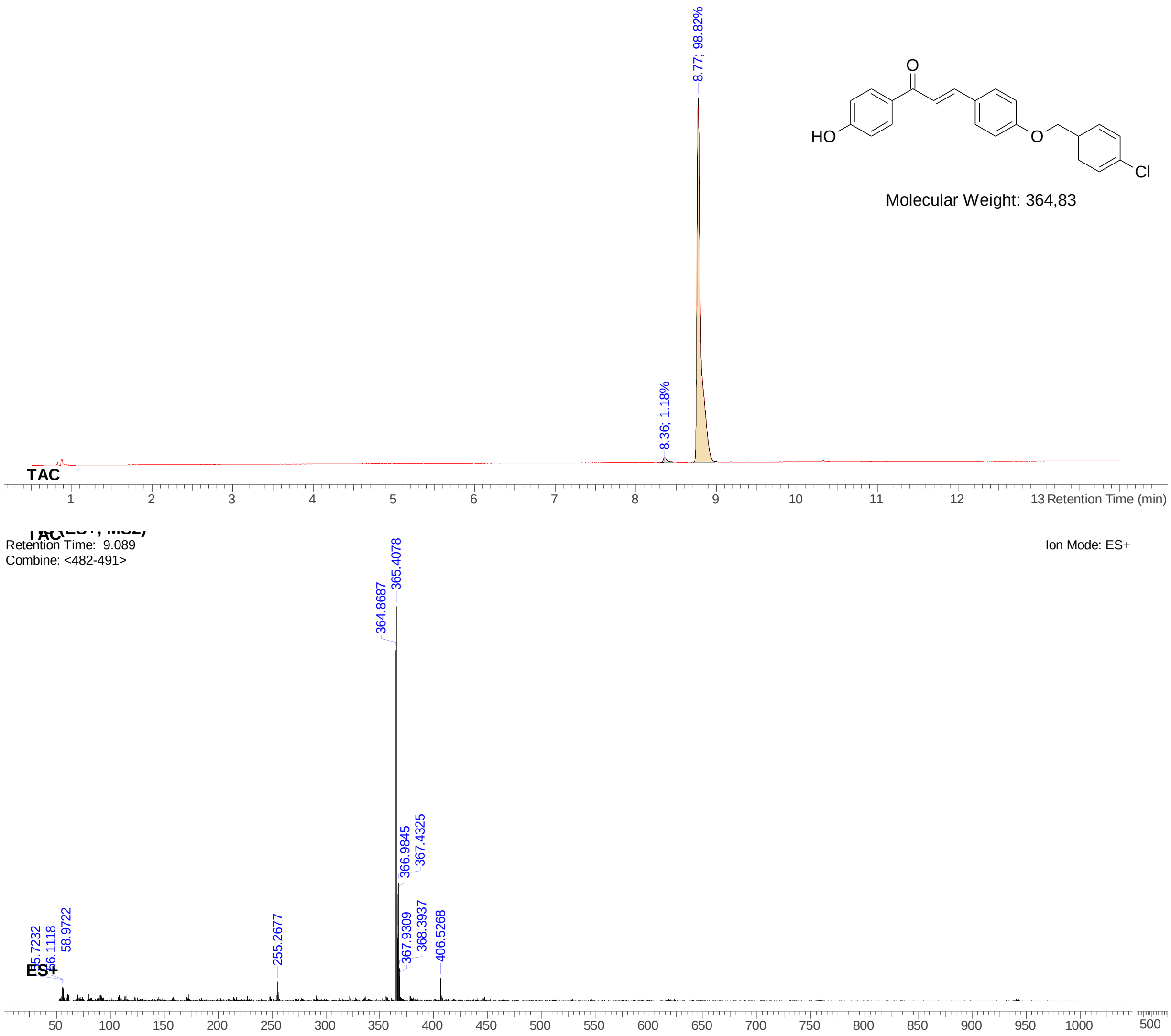

Figure S42. LC-MS assessment of the purity of compound **8**. The purity of compound **8** is 98.82% (retention time: 8.77 min).

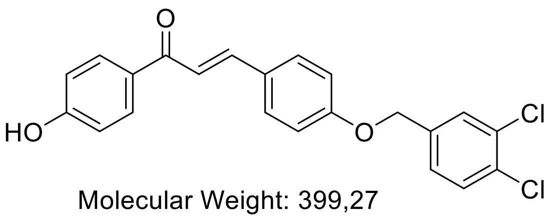

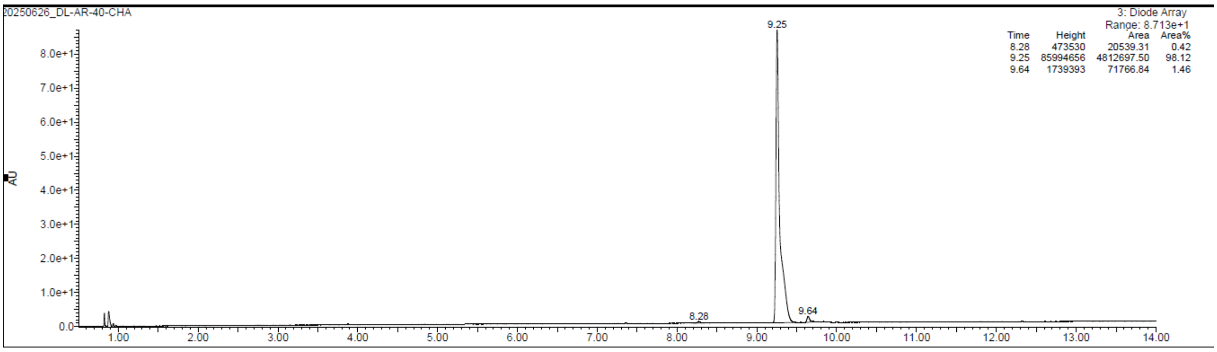

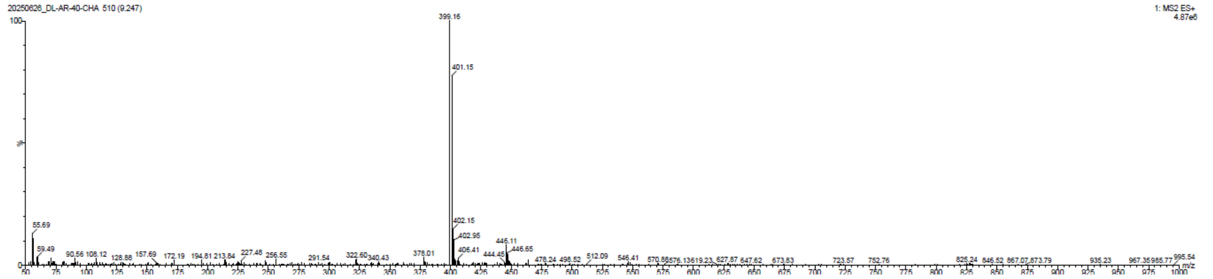

Figure S43. LC-MS assessment of the purity of compound **9**. The purity of compound **9** is 98.12% (retention time: 9.25 min).

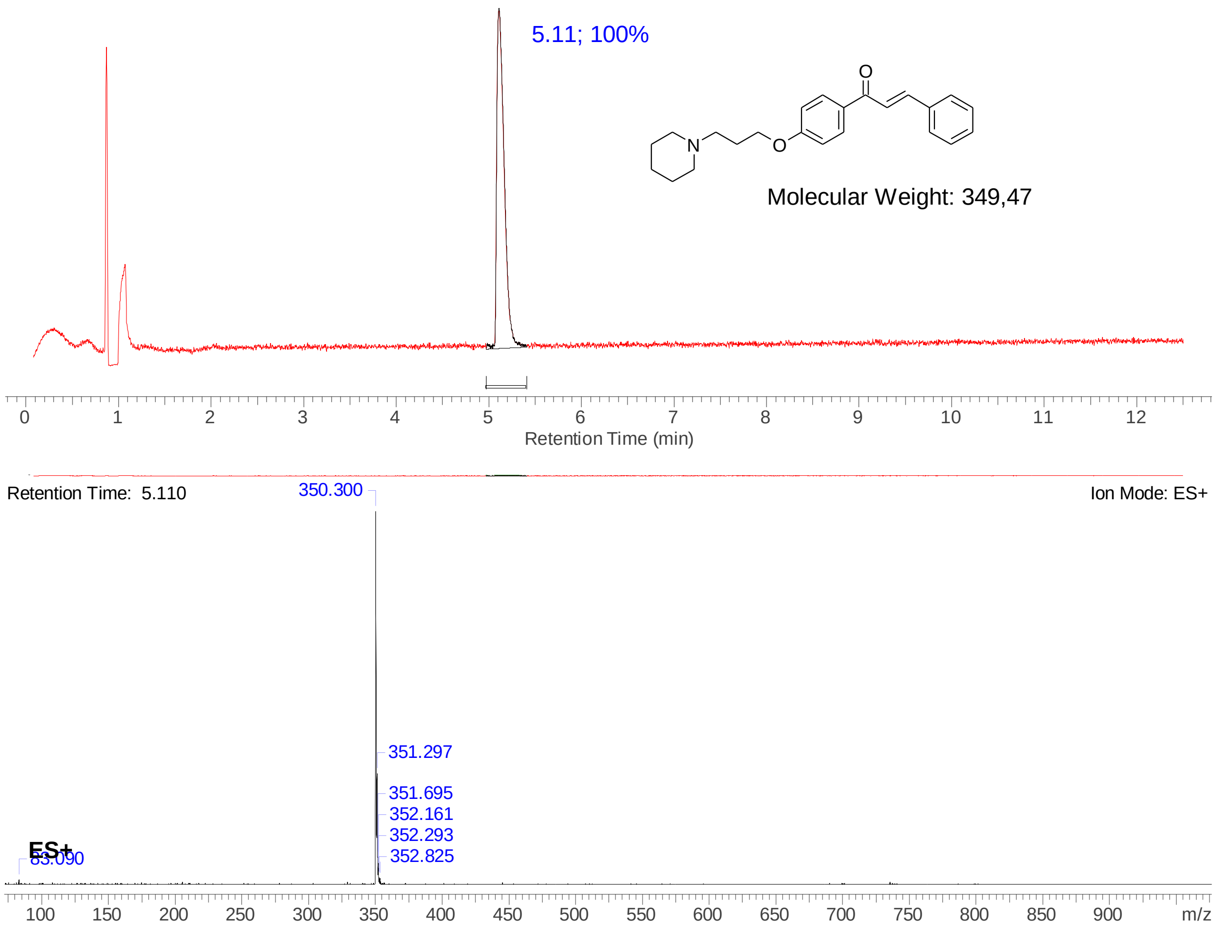

Figure S44. LC-MS assessment of the purity of compound **10**. The purity of compound **10** is 100% (retention time: 5.11 min).

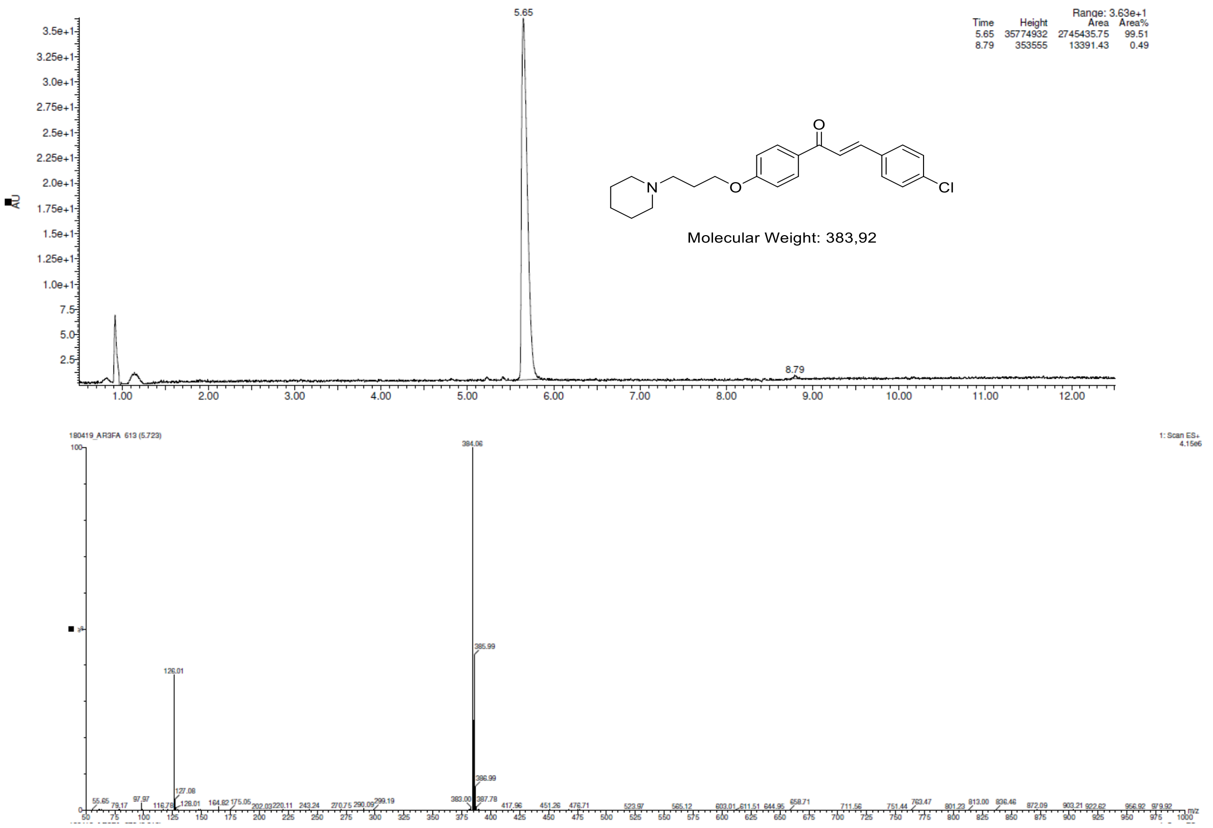

Figure S45. LC-MS assessment of the purity of compound **11**. The purity of compound **11** is 99.51% (retention time: 5.65 min).

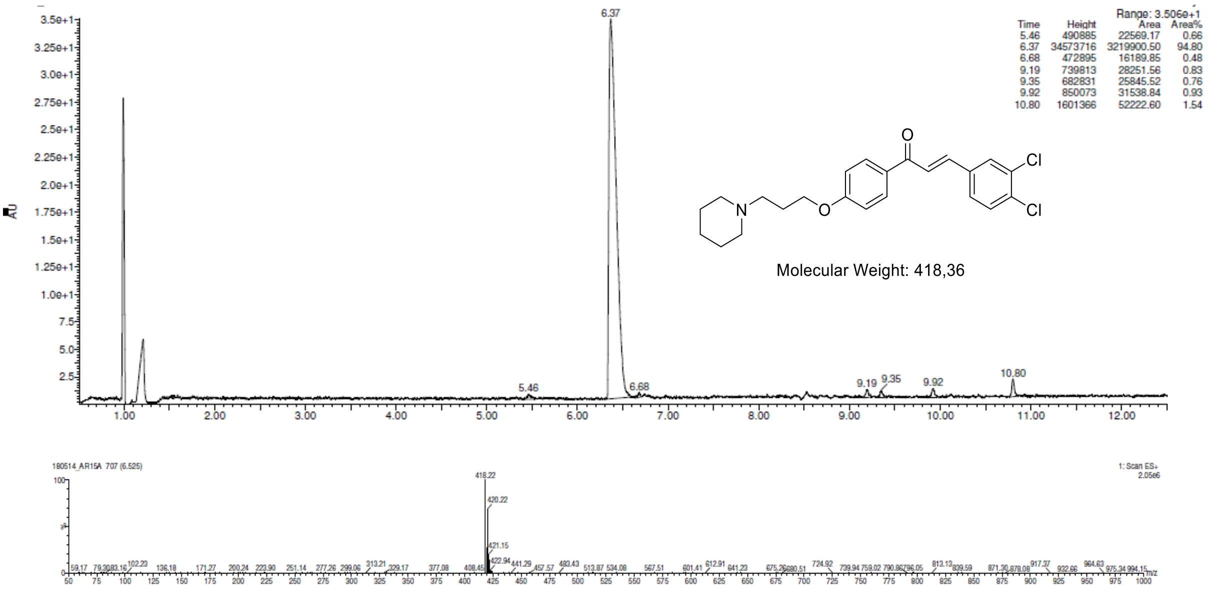

Figure S46. LC-MS assessment of the purity of compound **12**. The purity of compound **12** is 94.80% (retention time: 6.37 min).

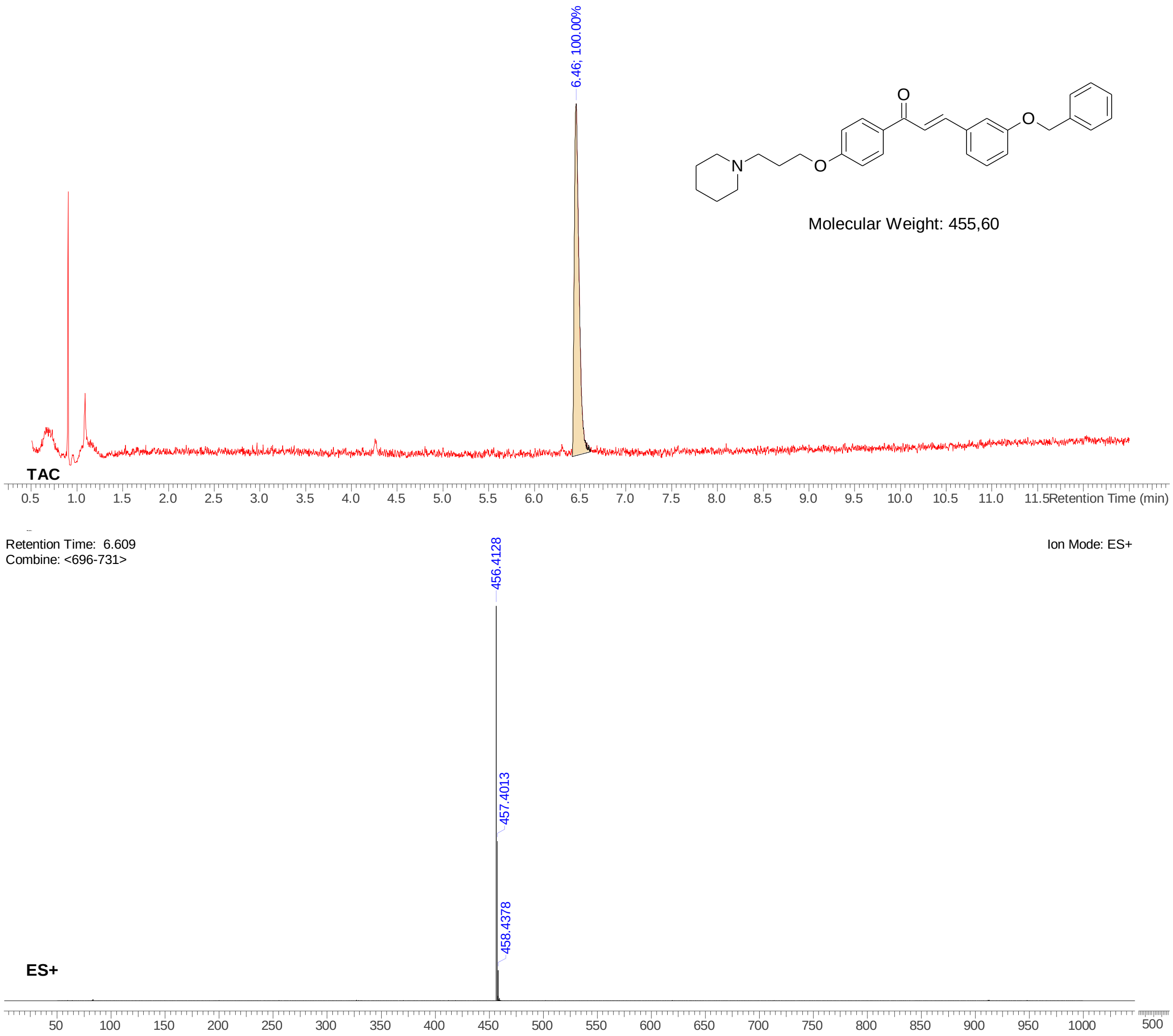

Figure S47. LC-MS assessment of the purity of compound **13**. The purity of compound **13** is 100% (retention time: 6.46 min).

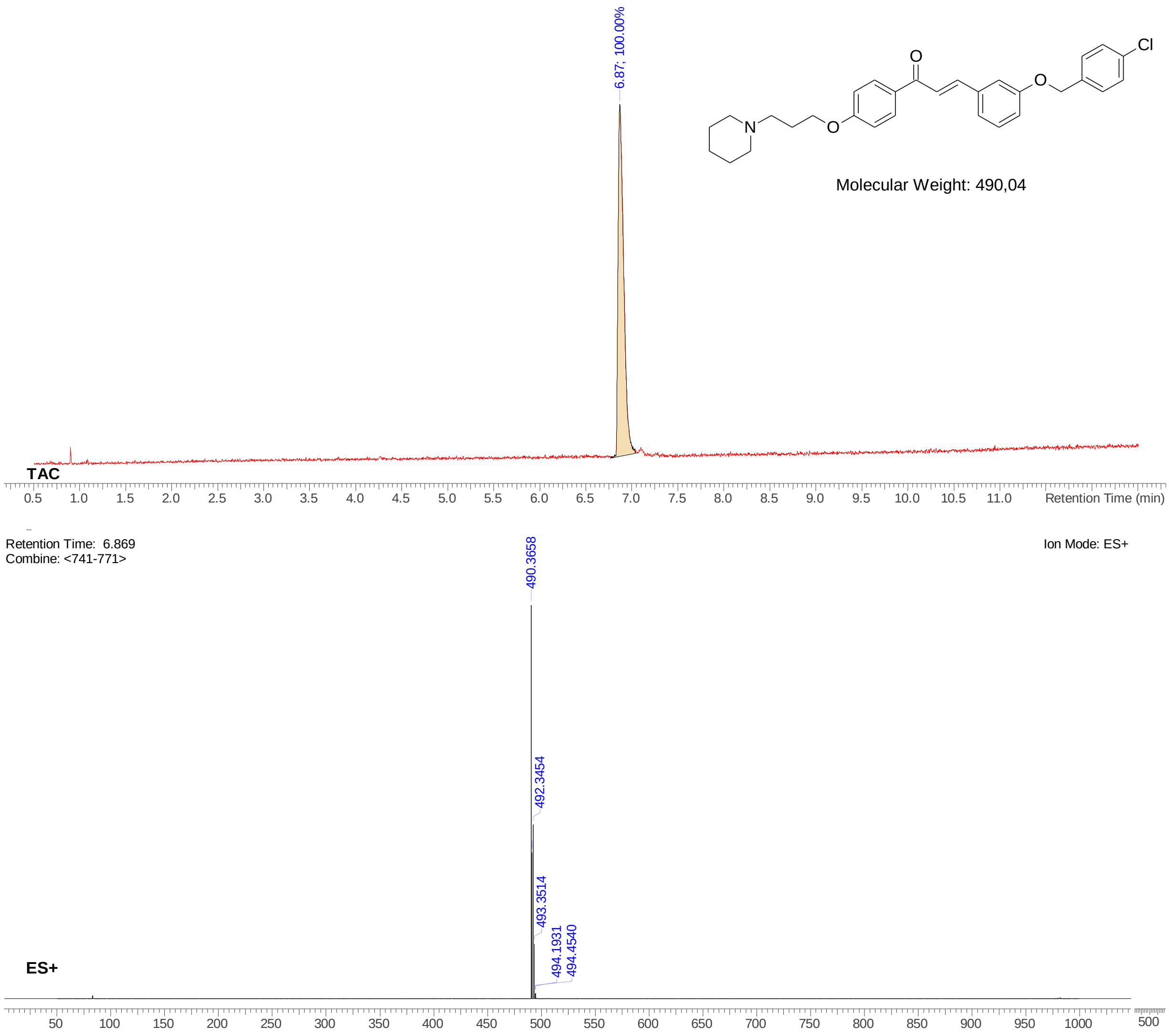

Figure S48. LC-MS assessment of the purity of compound **14**. The purity of compound **14** is 100% (retention time: 6.87 min).

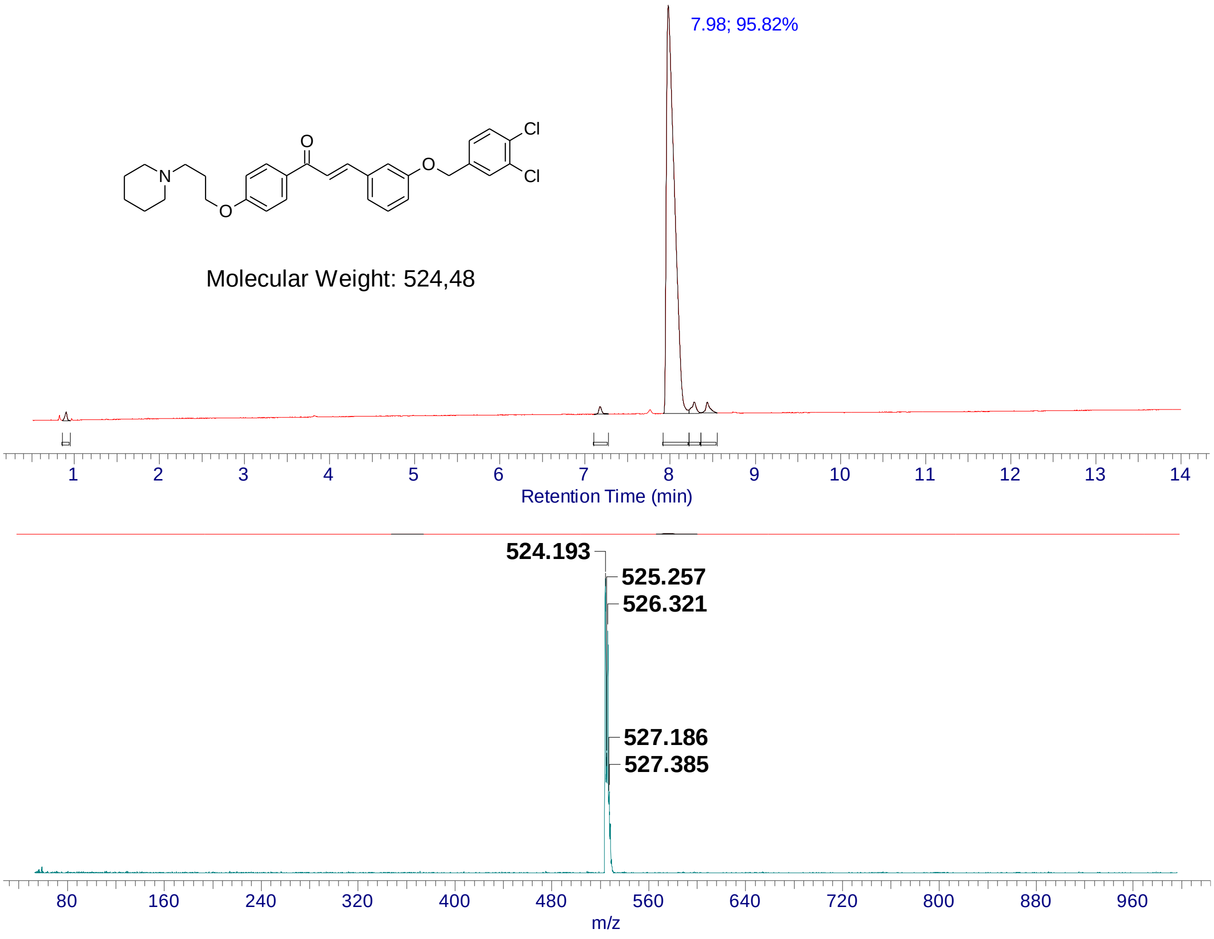

Figure S49. LC-MS assessment of the purity of compound **15**. The purity of compound **15** is 100% (retention time: 6.87 min).

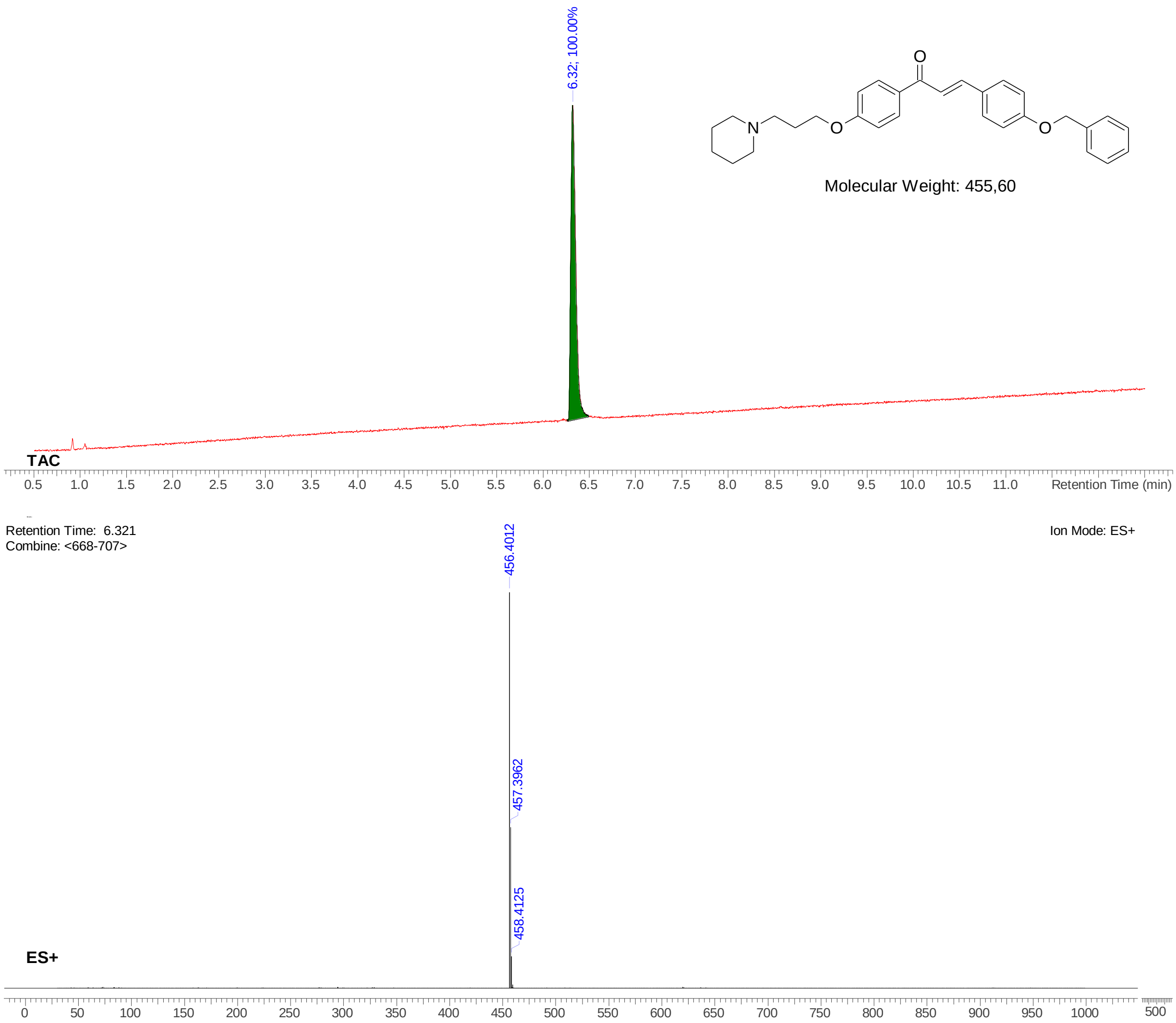

Figure S50. LC-MS assessment of the purity of compound **16**. The purity of compound **16** is 100% (retention time: 6.32 min).

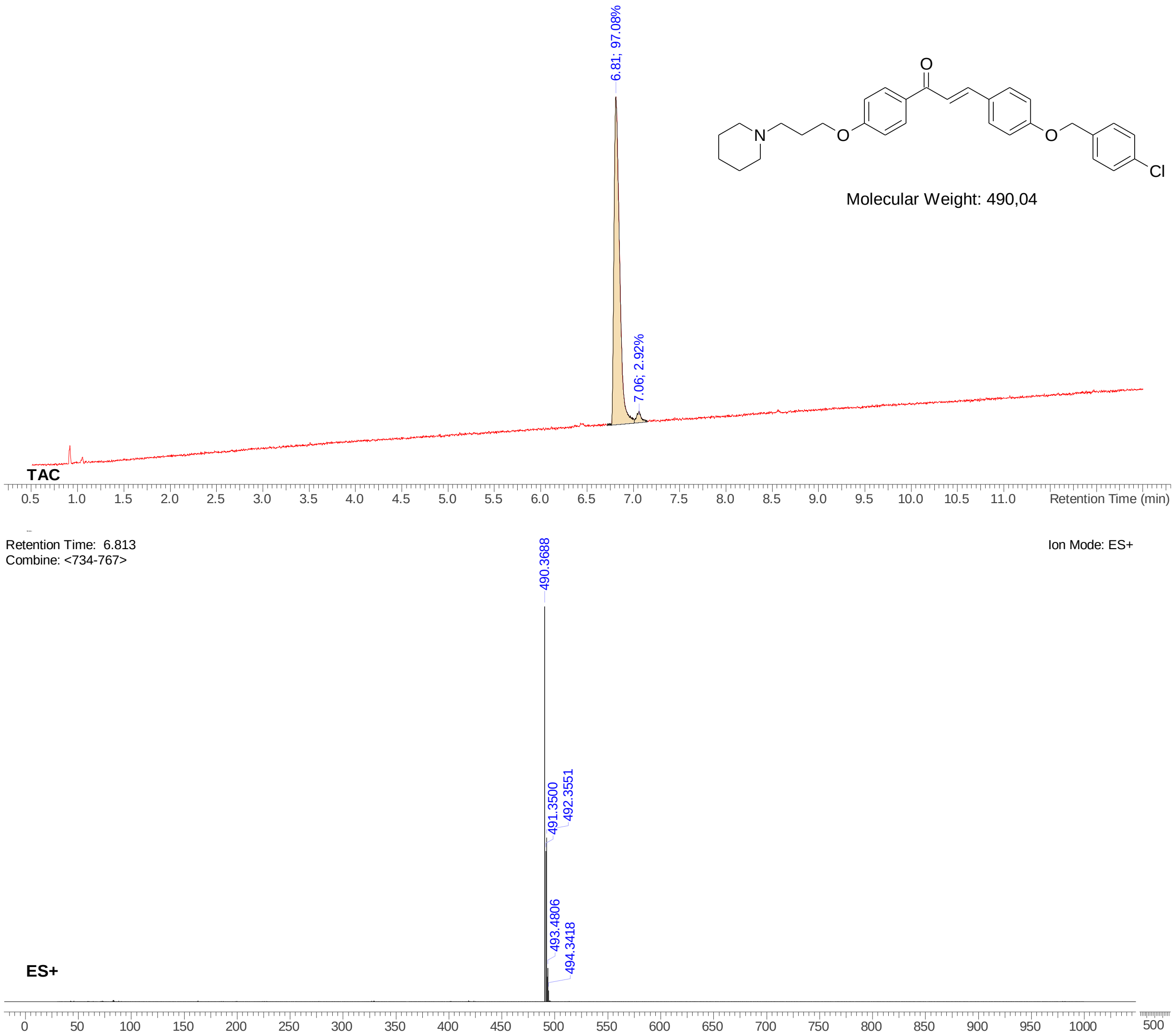

Figure S51. LC-MS assessment of the purity of compound **17**. The purity of compound **17** is 97.08% (retention time: 6.81 min).

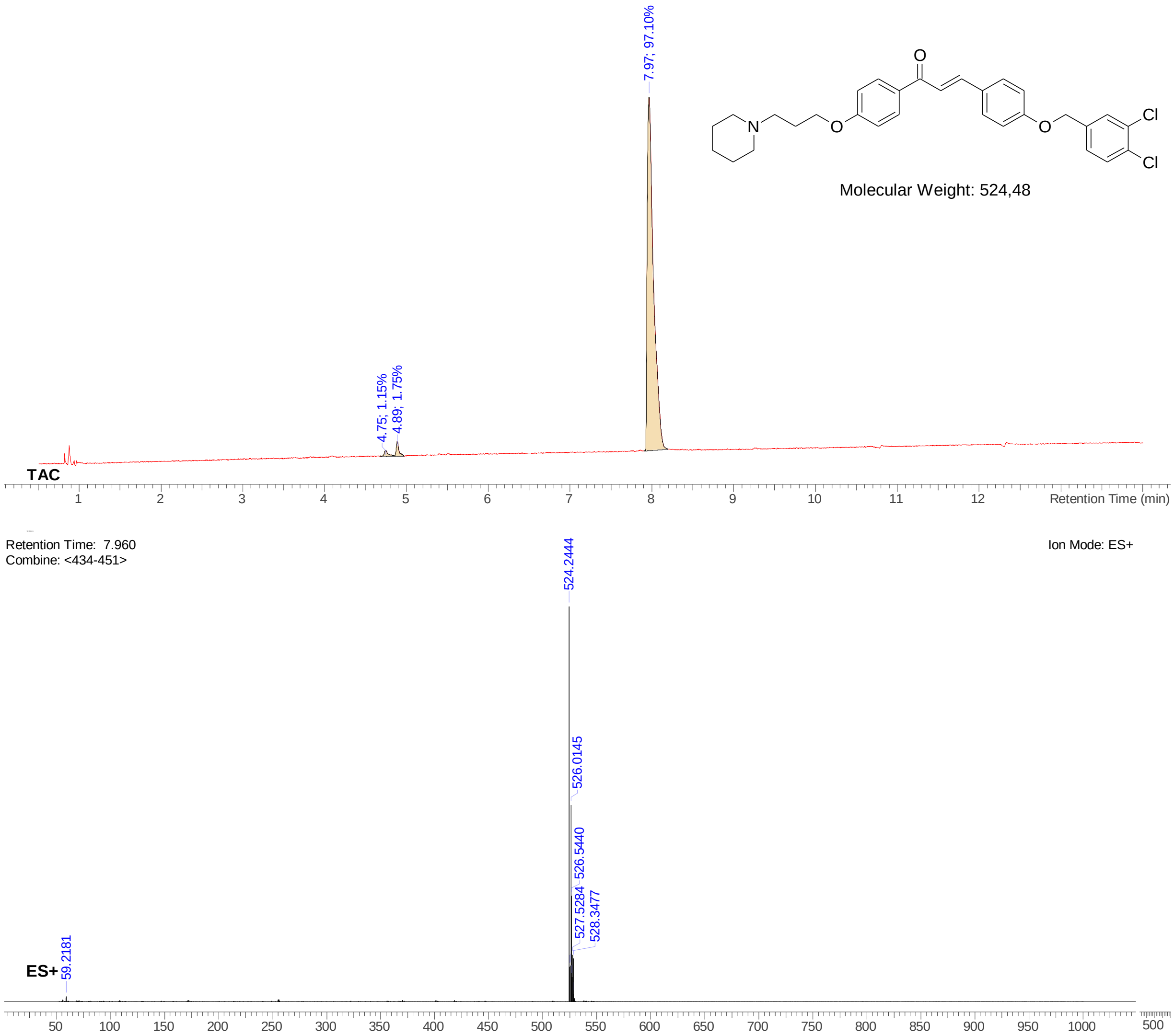

Figure S52. LC-MS assessment of the purity of compound **18**. The purity of compound **18** is 97.10% (retention time: 7.97 min).
